# Presence of Apis Rhabdovirus-1 in Populations of Pollinators and Their Parasites from Two Continents

**DOI:** 10.3389/fmicb.2017.02482

**Published:** 2017-12-12

**Authors:** Sofia Levin, David Galbraith, Noa Sela, Tal Erez, Christina M. Grozinger, Nor Chejanovsky

**Affiliations:** ^1^Department of Entomology, Institute of Plant Protection, Agricultural Research Organization, Rishon LeZion, Israel; ^2^Faculty of Agricultural, Food and the Environmental Quality Sciences, The Hebrew University of Jerusalem, Rehovot, Israel; ^3^Department of Entomology – Center for Pollinator Research – Huck Institutes of the Life Sciences, Pennsylvania State University, University Park, PA, United States; ^4^Department of Plant Pathology and Weed Research, Institute of Plant Protection, Agricultural Research Organization, Rishon LeZion, Israel; ^5^Institute of Bee Health, Vetsuisse Faculty, University of Bern, Bern, Switzerland

**Keywords:** rhabdovirus, honey bees, bumble bees, *Varroa destructor* mites, pollinators

## Abstract

The viral ecology of bee communities is complex, where viruses are readily shared among co-foraging bee species. Additionally, in honey bees (*Apis mellifera*), many viruses are transmitted – and their impacts exacerbated – by the parasitic *Varroa destructor* mite. Thus far, the viruses found to be shared across bee species and transmitted by *V. destructor* mites are positive-sense single-stranded RNA viruses. Recently, a negative-sense RNA enveloped virus, Apis rhabdovirus-1 (ARV-1), was found in *A. mellifera* honey bees in Africa, Europe, and islands in the Pacific. Here, we describe the identification – using a metagenomics approach – of ARV-1 in two bee species (*A. mellifera* and *Bombus impatiens*) and in *V. destructor* mites from populations collected in the United States and Israel. We confirmed the presence of ARV-1 in pools of *A. mellifera*, *B. impatiens*, and *V. destructor* from Israeli and U.S. populations by RT-PCR and found that it can reach high titers in individual honey bees and mites (10^7^–10^8^ viral genomic copies per individual). To estimate the prevalence of ARV-1 in honey bee populations, we screened 104 honey bee colonies across Israel, with 21 testing ARV-1-positive. Tagged-primer-mediated RT-PCR analysis detected the presence of the positive-sense ARV-1 RNA in *A. mellifera* and *V. destructor*, indicating that ARV-1 replicates in both hosts. This is the first report of the presence of ARV-1 in *B. impatiens* and of the replication of a rhabdovirus in *A. mellifera* and *V. destructor*. Our data suggest that *Varroa* mites could act as an ARV-1 vector; however, the presence of ARV-1 in *B. impatiens* (which are not parasitized by *Varroa*) suggests that it may not require the mite for transmission and ARV-1 may be shared among co-foraging bee species. Given that ARV-1 is found in non-Apis bee species, and because “ARV” is used for the Adelaide River virus, we propose that this virus should be called bee rhabdovirus 1 and abbreviated BRV-1. These results greatly expand our understanding of the diversity of viruses that can infect bee communities, though further analysis is required to determine how infection with this virus impacts these different hosts.

## Introduction

Global populations of pollinator species have been experiencing serious declines (Biesmeijer, 2006; Potts et al., 2010; Goulson et al., 2015). In managed honey bee populations in the United States and Europe, beekeepers typically experience heavy colony losses every year, averaging 40% in the United States (Steinhauer et al., 2017). Pollinators are vital to production of many key agricultural crops, with about 75% of the major crops that are used worldwide for human consumption benefiting from insect – primarily bee – pollination (Klein et al., 2007); therefore, bee decline can have a long-term impact on food production (Klein et al., 2007; Winfree, 2008; Potts et al., 2010; Singh et al., 2010). It was estimated that the contribution of insects to agriculture was €153 billion in 2009 (Gallai et al., 2009), and these values are likely significantly higher if economic impact on downstream industrial sectors is taken into consideration (Chopra et al., 2015).

Viral infections have been implicated as a major factor underpinning colony decline and loss in honey bees (Rosenkranz et al., 2010; Cornman et al., 2012; Dainat et al., 2012a,b; Martin et al., 2012; Nazzi et al., 2012; Gisder and Genersch, 2015; McMenamin and Genersch, 2015). Honey bees are hosts to more than 20 viruses, most of which are positive-sense single-stranded RNA viruses (McMenamin and Genersch, 2015). Moreover, these positive-sense RNA viruses – such as acute bee paralysis virus (ABPV), deformed wing virus (DWV), Israeli acute paralysis virus (IAPV), Kashmir bee virus (KBV), Sacbrood virus (SBV), and Lake Sinai virus (LSV) (Bowen-Walker et al., 1999; Chen et al., 2004; Shen et al., 2005; Chen and Siede, 2007; de Miranda and Genersch, 2010; Di Prisco et al., 2011; Ravoet et al., 2015) – can be vectored by an ectoparasitic mite (*Varroa destructor*), a key parasite of honey bees. *V. destructor* parasitization exacerbates the negative effects of virus infections on a honey bee colony (Nazzi and Le Conte, 2016), it has been shown to enhance ABPV’s prevalence in *Apis mellifera* colonies (reviewed in Genersch and Aubert, 2010), and is associated with significant increases in DWV titers as well as in promoting lower diversity of viral genotypes (Yang and Cox-Foster, 2007; Martin et al., 2012; Nazzi et al., 2012; Mondet et al., 2014; Ryabov et al., 2014; Wilfert et al., 2016). Furthermore, DWV infection leads to increased *V. destructor* reproduction on infected bees (Di Prisco et al., 2016). *V. destructor*’s synergistic interactions with DWV has been strongly correlated with *A. mellifera* colony losses (Dainat et al., 2012a; Nazzi et al., 2012). Additionally, it has been suggested that increasing infections with RNA viruses have led to substantial decrease in mite infestation thresholds leading to honey bee colony loss (Sumpter and Martin, 2004; Le Conte et al., 2010).

Importantly, many viruses first detected in honey bees have been shown to be able to infect other pollinator species (reviewed in Tehel et al., 2016). Again, primarily positive-sense RNA viruses have been shown to infect non-honey bee species, including the same viruses that are transmitted by *V. destructor* to honey bees, namely ABPV, DWV, IAPV, KBV, SBV, and LSV (Tehel et al., 2016). Viral transmission appears to occur via co-foraging on infected flowers (Singh et al., 2010; Fürst et al., 2014). As generalist foragers with large foraging ranges and high population numbers, honey bees could be sharing pathogens with multiple pollinator species in any given area (Geslin et al., 2017). The degree to which these viruses negatively impact other pollinators remains to be determined (Genersch et al., 2006; Dolezal et al., 2016; Tehel et al., 2016; Melathopoulos et al., 2017).

Little information is available on negative-sense RNA viruses in pollinator communities. Recently, Remnant et al. (2017) describe the identification of two negative-sense strand viruses, Apis rhabdovirus-1 (ARV-1) and Apis rhabdovirus-2 (ARV-2) in *A. mellifera* colonies from three locations (Netherlands, South Africa, and South Pacific) as well in Varroa mites associated with the colonies. Rhabdoviruses are negative-sense RNA enveloped viruses, whose particles are 100–430 nm long and 45–100 nm in diameter. The viral genome encodes five structural proteins: RNA-dependent RNA-polymerase (L), a phosphorylated phosphoprotein (P), a nucleoprotein (N), the matrix protein (M), and the envelope glycoprotein (G) (Kuzmin et al., 2009). It was reported that rhabdovirus genomes bear additional putative proteins from alternative or overlapping open-reading frames (ORFs) within the major structural protein genes or from independent ORFs between the structural protein genes (reviewed in Walker et al., 2015). Analysis using the L protein sequences of ARV-1 and ARV-2, that displayed 30% and 23% of identity to that of the Farmington virus of birds (FARV-1; Palacios et al., 2013), showed that they form a monophyletic group with FARV-1 (Remnant et al., 2017).

To gain insight about the presence and distribution of negative-sense RNA viruses in honey bees and other bees, we used high-throughput sequencing to examine the transcriptomes and viromes of honey bee (*Apis mellifera*) populations in the United States and Israel, a bumble bee (*Bombus impatiens*) population in the United States, as well as populations of the ectoparasitic mite of honey bees *V. destructor* in Israel. We identified ARV-1 in these samples, and characterized its distribution, prevalence, and infectivity. We confirmed the presence of viral copies in honey bees (from both continents), *B. impatiens* and *V. destructor*, and estimated the viral prevalence among 104 honey bee colonies as well as its titers in individual *A. mellifera* bees and *V. destructor* mites. Importantly, we examined whether ARV-1 is actively replicating in *A. mellifera* and *V. destructor* mites. Replication of single-stranded sense RNA viruses requires the synthesis of the complementary positive-sense RNA, which can be detected by using strand-specific RT-PCR (Horsington and Zhang, 2007). Using this approach we found evidence that ARV-1 was able to replicate in *A. mellifera* and *V. destructor* mites.

## Materials and Methods

### Sample Collection

#### Israeli Samples

The honey bee colonies (*A.mellifera ligustica*) from the Agricultural Research Organization (ARO) used for transcriptome and virome analysis in Israel were maintained without treatment against *Varroa* for 30 months before sampling and received seasonal sugar feeding and Fumagilin treatment against *Nosema*. For validation, prevalence, and replication studies, nurse honey bees were collected from apiaries located at the North (Galilee, Kibbutz Dan Apiary), the Center (Kfar Ruth), and the South (Kibbutz Yad Mordechai) of the country.

#### U.S. Samples

Honey bees (*A. mellifera*) and bumble bees (*B. impatiens*) were collected in State College, PA, United States (40.821419, -78.14635), while co-foraging on flowering plants. Individuals from the different bee species were collected in separate tubes to ensure that the species were not mixed. At least 20 individuals were collected onto ice-cold 95% ethanol, and ultimately stored at -20°C until the samples were processed. Additionally, honey bees that exhibited symptoms of DWV infection were collected from a single colony from a Pennsylvania State University apiary in State College using the same protocol.

### Preparation of U.S. and IL Samples

#### Israeli Samples

RNA was extracted from honey bees and *V. destructor* mites samples from four ARO colonies and subsequently pooled (30 bees and 310 mites for *A. mellifera* and *V. destructor* cDNA libraries, respectively). RNA was extracted using TRI reagent^®^ (Sigma–Aldrich) according to the manufacturer’s instructions in a Geno/grinder homogenizer (Metuchen, NJ, United States) following further purification by precipitation with 2.5 M lithium chloride as described previously (Levin et al., 2016). The quality and quantity of the extracted RNA was evaluated using an Agilent bio-Bioanalyzer (Agilent Technologies).

#### U.S. Samples

Ten individuals from each sample group (field-collected *B. impatiens*, field-collected *A. mellifera*, and colony-collected *A. mellifera* with deformed wings) were placed individually in 2.0 ml nuclease-free microcentrifuge tubes with molecular grade H_2_O with three to five sterile glass beads and homogenized for 45 s in a FastPrep FP120 Cell Disruptor (Thermo-Fisher Scientific, Waltham, MA, United States). Samples were stored on ice for 10 min, and the previous step was repeated to ensure adequate homogenization. Hundred microliters of the homogenate from each of the 10 samples was pooled in a common 1.5 ml microcentrifuge tube. The resulting homogenate was passed through a 0.2-μm cell filter (Corning, Tewksbury, MA, United States) to purify the virus extracts as in Hunter et al. (2010) and Liu et al. (2010). One hundred and twenty-five microliters of each sample was treated with a nuclease cocktail (including 14 U Turbo DNase I, 25 U Benzonase, 20 U RNase I, and 10× DNase buffer) and incubated at 37°C for 1.5 h to remove all nucleic acid that is not protected by a viral capsid as in He et al. (2013). Nucleic acids (both RNA and DNA) from the purified encapsulated viruses were extracted using a MagMAX Viral Isolation Kit (Thermo-Fisher Scientific, Waltham, MA, United States), according to the manufacturer’s protocol.

### Transcriptome and Virome Analysis

#### Israeli Samples

High-throughput sequencing of the RNA from the honey bees and *V. destructor* mites samples from Israeli colonies was described previously (Levin et al., 2016). Complementary Sanger sequencing was performed at the Weizmann Institute of Science (Rehovot, Israel) with primers indicated in Supplementary Table S1. Note that Levin et al. (2016) focused on the identification of viruses that were unique to *V. destructor* mites, and thus information related to ARV-1 (which was found in both samples) is newly described in the current manuscript.

#### U.S. Samples

A previously established protocol was used to obtain unbiased random amplification of the extracted nucleic acids (Ng et al., 2012). Briefly, the first strand cDNA was synthesized from 30 μl of viral nucleic acid using a random primer design (consisting of a 20 base oligonucleotide sequence followed by a randomized octamer sequence: GACCATCTAGCGACCTCCACNNNNNNNN) in a reverse transcriptase (RT) reaction using a High-Capacity cDNA Reverse Transcription Kit (Thermo-Fisher Scientific, Waltham, MA, United States). To synthesize the second strand, the 19 μl of the initial cDNA was denatured at 95°C for 2 min and cooled to 4°C prior to the addition of Klenow Fragment DNA Polymerase (New England Biolabs, Ipswich, MA, United States) for fragment extension at 37°C for 60 min. Finally, PCR amplification was performed for 40 cycles using the above primer without the randomized octamer sequence (GACCATCTAGCGACCTCCAC) and 2 μl of dsDNA template. The PCR reactions were then purified using a MSB Spin PCRapace Purification Kit (Invitek, Berlin, Germany) using the manufacturer’s standard protocol. Quality and concentration of the samples were assessed using a 2100 Bioanalyzer (Agilent, Santa Clara, CA, United States). The samples were then submitted to the Genome Core Facility at Pennsylvania State University for sequencing on the Illumina MiSeq, resulting in ∼1 × 10^6^ reads of 150 nucleotides each per sample.

### Bioinformatic Identification of ARV-1 Contigs

#### Israeli Samples

The transcriptome and virome data were analyzed as described before (Levin et al., 2016). The transcriptome and virome files of *V. destructor* and *A. mellifera* were uploaded to SRA database under accession numbers PRJNA329427 and PRJNA329428, respectively. The sequences were cleaned from remains of adaptor sequences and low quality reads using Trimmomatic Software (Bolger et al., 2014) and assembled *de novo* using Trinity (Haas et al., 2013). The assembled contigs were subsequently translated and aligned to the GenBank nr database (all nr database without filtering) by BLASTx (Altschul et al., 1997) (with a cut-off value of <1*e*^-5^) (Corpet, 1988).

#### U.S. Samples

The quality of the sequencing run was assessed using FastQC (Andrews, 2010) to ensure only high-quality sequences were used for further analyses. Adaptor sequences and reads with low-quality scores were removed using Trimmomatic (Bolger et al., 2014). Processed reads were assembled into longer contigs using three *de novo* assembly programs; SPAdes (Bankevich et al., 2012), Velvet/Oases (Schulz et al., 2012), and Trinity (Grabherr et al., 2011; Haas et al., 2013). To improve the overall assembly, the results from these three assemblies were consolidated into a single assembly using Mix (Soueidan et al., 2013). To identify homologous sequences, the assembled contigs were compared to the viral sequences in the NCBI nr database (Pruitt et al., 2007) using BLASTx (Altschup et al., 1990) using an *E*-value cutoff of 10^-10^. ORF-finder (Rombel et al., 2002) was used to identify potential ORF within the contigs with significant BLAST hits to known viruses to further characterize the viral contigs from each sample. Contig sequences with significant BLAST hits for ARV-1 and Farmington virus are reported here, the remaining sequences will be reported in a future study (Galbraith et al., in preparation).

### Molecular Analysis

**FIGURE 1:**
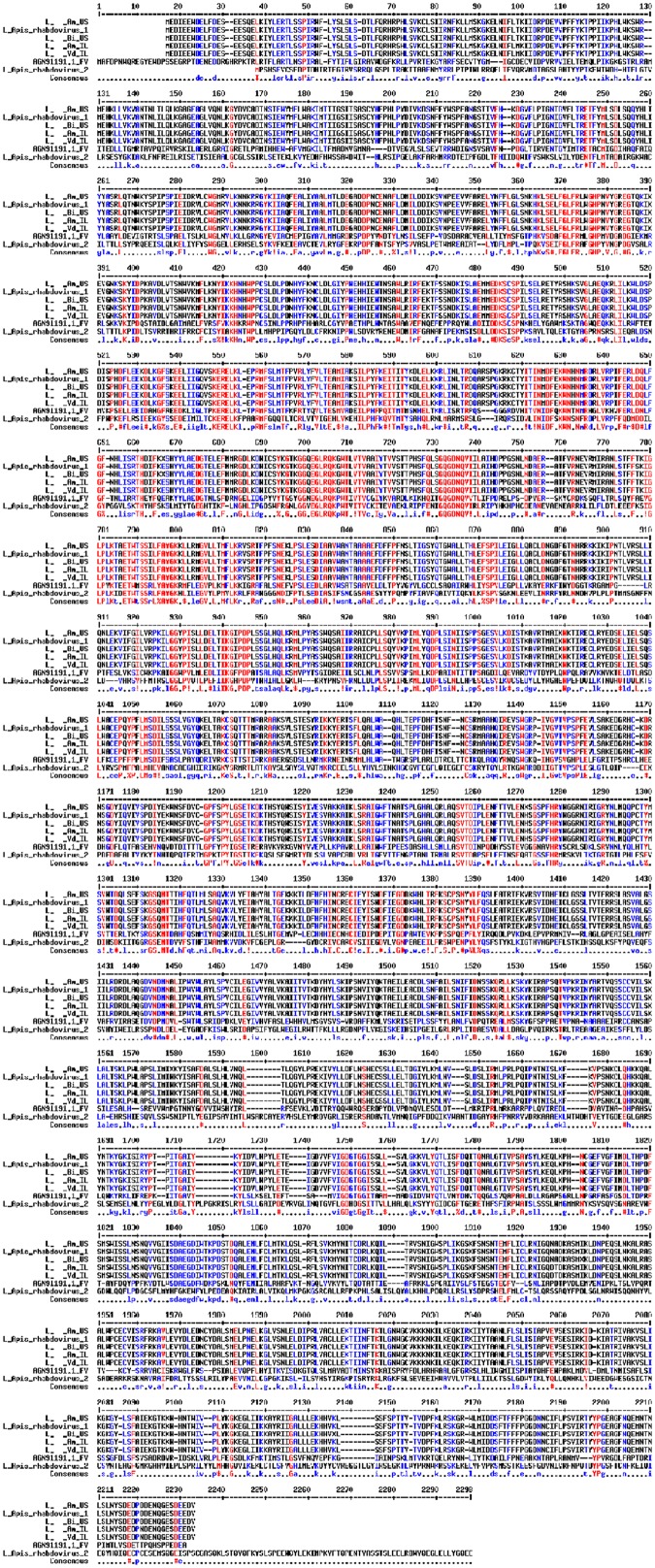
Alignment of the L proteins of the rhabdovirus from *A. mellifera* (Am_US and Am_ IL), *B. impatiens* (Bm_US), and *V. destructor* (Vd_IL) to ARV-1, ARV-2, and Farmington virus of birds (GenBank KY354232.1, KY354234.1, and YP_009091822.1, respectively). Amino acid residues in red, blue, and black are fully, partially, and not identical among all the aligned sequences.

The complete nucleotides and predicted ORF of ARV-1 from the four sample groups (U.S. *A. mellifera*, Israeli *A. mellifera*, U.S. *B. impatiens*, and Israeli *V. destructor*) were used for the analysis (**Figure 1** and Supplementary Figure S1). Protein Alignment was done using the web interface of MultAlin^1^ (Corpet, 1988). The identified rhabdovirus sequences were deposited in NCBI GenBank (accession numbers MF114348–MF114351).

### RT-PCR Samples

#### Israeli Samples

Nurse bees and *V. destructor* mites were collected and RNA was extracted from individuals using TRI reagent as described above. cDNA was prepared using Maxima-RT (Fermentas-Thermo, Fisher Scientific, Burlington, Canada) using oligo-dT and random primers according to the manufacturer’s instructions. Hundred nanograms of template RNA was used for screening and a second re-screening was performed for honey bee samples with 200 ng of the same RNA. RT-conditions: incubation of RNA and primers at 65°C 5 min, followed by addition of buffer containing 50 mM Tris–HCl (pH 8.3), 75 mM KCl, 2 mM MgCl_2_, 5 mM DTT, 4 units of RNase inhibitor Ribolock^®^ (Thermo Scientific), the RT enzyme (200 units) in a 20-μl volume, and further incubation at 55°C for 30 min. The reaction was terminated by heating at 85°C for 5 min.

PCRs were performed using LongAmp Taq Polymerase (New England Biolabs, Ipswich, MA, United States) and specific primers (see Supplementary Table S1) in a BioER GenePro TC-E-96G apparatus (Hangzhou Bori Technology Co., Ltd., P.R. China). For long templates amplification 1 μl cDNA was used with 2.5 units of Taq DNA polymerase with enzyme buffer, 10 mM dNTP, 2 mM MgCl_2_, 2% DMSO, and 0.2 μM of each forward and reverse primer in a final reaction volume of 25 μl. The protocol used was 95°C for 4 min, then 35 cycles of 94°C for 20 s, 55°C for 1 min, 65°C for 8 min, and a final extension step of 65°C for 10 min. PCR validations were performed with GoTaq^®^ (Promega Corporation, United States) with 1.5 mM MgCl_2_, 1 μl cDNA template, and 0.2 μM of each forward and reverse primer in a 20 μl reaction with the following conditions: 95°C for 4 min, 30 cycles at 94°C for 30 s, then 58°C for 50 s, 72°C for 2 min, and a final extension step of 72°C for 10 min. Amplification and validation primers are provided in Supplementary Table S1, and in the corresponding legends of the figures. All the PCR reactions included non-template controls (NTC). PCR products were evaluated by conventional agarose electrophoresis.

Additionally, Sanger sequencing (performed at the Biological Services Unit of the Weizmann Institute of Science, Israel) was used to confirm that the amplified products indeed corresponded to the expected ARV-1 sequences. Also, these sequences allowed us to validate gaps in sequence data obtained from small contigs and to compare it to the nucleotide sequence obtained from the largest contig of 14606 (the primer sequences used to analyze the termini and subsequent gaps are provided in Supplementary Table S1). The sequencing perfectly matched the large nucleotide sequence obtained in the above large contig.

#### U.S. Samples

The RT-PCR was performed on the same individuals originally homogenized for the virome analysis (described above). For each individual bee used in the virome analysis, 50 μl of the homogenate was used for RNA extraction using an RNAeasy Kit (Qiagen, Valencia, CA, United States). cDNA was prepared using a High-Capacity cDNA Reverse Transcription Kit (Applied Biosystems, Foster City, CA, United States) using the standard protocol provided by the manufacturer. Specifically, 200 ng of template RNA was mixed with 2 μl 10× RT Buffer, 3.2 mM dNTP Mix, 2 μl 10× RT Random Primers, 50 units of MultiScribe Reverse Transcriptase^TM^, 20 units of RNase inhibitor, and additional H_2_O to bring the reaction volume to 20 μl. The reaction was performed according to the manufacturer’s protocol: the solution was heated to 25°C for 10 min, 37°C for 120 min, 85°C for 5 min, and then cooled to 4°C.

The cDNA product was then amplified with PCR using primers described in Supplementary Table S1. According to the manufacturer’s protocol, 2 μl of cDNA template was mixed with 0.2 μM of each Forward and Reverse primer, 2.5 μl 10× Taq buffer, 0.2 mM dNTPs, 0.75 units of Taq DNA polymerase (New England Biolabs, Ipswich, MA, United States), and 18.875 μl of H_2_O, in a final reaction volume of 25 μl. The solution was heated to 95°C for 4 min, followed by 30 cycles at 94°C for 30 s, 58°C for 50 s, and 72°C for 2 min, with a final extension step of 72°C for 10 min, and finally cooled to 4°C. All the PCR reactions included NTCs. PCR products were evaluated by conventional agarose electrophoresis to identifying PCR products at the expected sizes.

### qRT-PCR Assay

cDNA was prepared from individual nurse bees and *V. destructor* mites as described in the “RT-PCR – Israeli samples” section above. Quantitative PCR amplifications were performed on a PikoReal96 machine (Thermo-Fisher Scientific, Waltham, MA, United States) using a standard protocol (95°C 2 min; 40 cycles: 95°C 10 s, 60°C 20 s, 72°C 20 s). Each quantitative PCR analysis was performed in triplicate, in a 96-well PCR plate sealed with an optical adhesive cover (Thermo-Fisher Scientific, Waltham, MA, United States). Non-template controls (water) were included in triplicates in each assay. The KAPA SYBR FAST qPCR Master Mix (2×) Universal (Kapa Biosystems) was used in 10 μl final volume. For each analysis, 2 μl of the diluted cDNA was used (dilution factor of 4) and specific primers BRV-qRT-F1 and BRV-qRT-R1 concentration were 0.25 μM (Supplementary Table S1). The efficiency of the PCR reaction was as follows: *E* = 97%, *R*^2^ = 0.9979, and the slope = -3.387. The specificity of the amplicons synthesized during the PCR run was ascertained by performing a dissociation curve protocol from 60 to 95°C.

Amplicons of 182 bp containing the ARV-1 target sequence were obtained by performing PCR with the primers BRV-qRT-F1 and BRV-qRT-R1. A 10-point standard curve was prepared [fourfold serial dilutions of the obtained amplicon with known concentrations from 4 pg (Cq = 8.5) and up to 1.5 × 10^-5^ pg (Cq = 27.5)]. To establish a calibration curve for the quantification of the *V. destructor* mRNA for cytoplasmic actin reference, a 336 bp *V. destructor* DNA fragment was amplified with the primers VcytoactinF and VcytoactinR (Supplementary Table S1). After 34 amplification cycles, the DNA fragments were gel purified and aliquots were used for measuring their DNA concentration using a NanoDrop (Thermo-Fisher Scientific, Waltham, MA, United States). For each quantitative PCR assay, *n*^2^ fold serial dilutions in water were made and each dilution was processed in triplicates on the same 96-well PCR plate where the samples were deposited. For each sample, both ARV-1 and *V. destructor* mRNA for cytoplasmic actin quantitative analysis were performed in separate wells. To set up a calibration curve of *A. mellifera*, the housekeeping primers were RPL8 F and RPL8 R (Evans et al., 2006) as described before (Zioni et al., 2011) (primers in Supplementary Table S1).

Individual ARV-1 loads of 100 ng total RNA extracted from the individual sample were calculated by plotting Ct values against the logarithm of the RNA copy number using the PikoReal^TM^ Software 2.2 (Thermo-Fisher Scientific, Waltham, MA, United States). These values were used to calculate the ARV-1 copy number in the total RNA extracted from the individual sample.

### Replication Assay

Using the Israeli samples from individual nurse honey bees and *V. destructor* mites collected for the RT-PCR analysis, a replication analysis (presence of the positive strand-sense RNA) was performed. To avoid artifactual positive results caused by false priming (Vashist et al., 2012), we synthesized tagged primers and used them in the detection of the positive strand by RT-PCR as described before for analysis of replication of the RNA virus DWV of bees (Yue and Genersch, 2005). The negative-strand cDNA from the RNA samples was produced using the tagged-primer BRV-8711F-TAG and subsequently performing PCR with primers BRV-10356-R and TAG-D_F (Supplementary Table S1). cDNA produced without any primer was used as control followed by PCR with the same primers from above. PCR was performed at 95°C for 4 min, 30 cycles at 94°C for 30 s, then 59°C for 50 s, 72°C for 1.5 min, and a final extension step of 72°C for 10 min. A second PCR reaction was performed using only the primer BRV-10356-R to control for the absence of unspecific priming by presence of residual BRV-8711F-TAG primer from the RT reaction (Supplementary Figure S4). The identity of the amplified fragment was confirmed by Sanger sequencing (performed at the Biological Services Unit of the Weizmann Institute of Science, Israel).

## Results

### Identification of Apis Rhabdovirus-1 (ARV-1) in Honey Bees, Bumble Bees, and *Varroa* Mites

BLASTx analysis revealed the presence of a large contig homologous to a rhabdovirus in the transcriptomes of *A. mellifera* bees sampled from Israeli honey bee colonies. A similar parallel analysis evaluating the viral transcriptomes of honey bees (*A. mellifera*) and bumble bees (*B. impatiens*) sampled from populations in the United States also detected this rhabdovirus. Furthermore, exploring the virome of *V. destructor* mites sampled in Israel, we were able to identify the virus in the mites. The identified sequences were most homologous to the nucleotide sequence of ARV-1 identified in *A. mellifera* [(GenBank KY354232.1, Remnant et al., 2017); see below].

Assembly of the sequences in the individual data sets generated viral contigs from the *A. mellifera* samples of 14,606 nucleotides (in the Israeli samples, the U.S. samples had a slightly smaller contig size of 13,842 nucleotides), a viral contig from the *B. impatiens* sample of 14,590 nucleotides, and a viral contig from the *V. destructor* sample of 14,589 nucleotides.

Alignment among the putative proteins generated from the viral contigs of Israeli honey bees, U.S. honey bees, U.S. bumble bees, and Israeli *V. destructor* mites showed 100% identity for the L, M, and P proteins and only one amino acid change for each of the N proteins (amino acid 255, K in the U.S. samples, and R in the Israeli samples) and G protein (amino acid 141, N in the U.S. samples, and S in the Israeli samples) (**Figure 1** and not shown). The honey bee, bumble bee, and *Varroa* rhabdoviruses L protein exhibited 99.9% identity to the L-protein of ARV-1 (only two changes amino acids 777 T to S and 981 V to I compared to ARV-1, respectively), and 35% identity for the L-protein of FARV (**Figure 1**). The proteins of the rhabdovirus we found were identical to that of ARV-1 (summarized in **Table 1**). BLASTn showed that the rhabdovirus genomes that we found had 99% similarity to that of ARV-1 and very low similarity (2% of the virus sequence with *e*-value of 0.16) to that of ARV-2 (GenBank KY354234.1, Remnant et al., 2017; and not shown). Thus, we concluded that the rhabdovirus we identified in honey bees, bumble bees, and *V. destructor* mites was ARV-1.

**Table 1:** Comparison between the putative proteins of Rhabdoviruses found, ARV-1 and FARV proteins.

Rhabdovirus	Length of	Similarity	FARV	Length of
proteins	putative	to ARV-1		protein
	ORF (aa)			(aa) in FarV
L	2143	99.9% identity	35% identity	2129
G	605	100% identity	21% identity	704
M	166	100% identity	28% identity	148
P	316	100% identity	No significant similarity	316
N	367	100% identity	26% identity	421


### Confirmation of ARV-1 Presence Using RT-PCR

**FIGURE 2:**
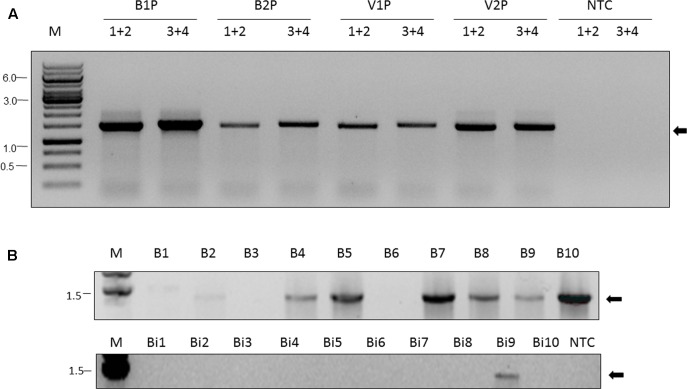
Apis rhabdovirus-1 is detected in *A. mellifera*, *B. impatiens*, and *V. destructor* by RT-PCR. **(A)** Pools of nurse bees (BP) and mites (VP), respectively, from Israeli colonies 1 and 2 (B1P, V1P and B2P, and V2P, respectively, indicated above the figure). 1+2 (primers BRV-1-2683-F and BRV-1-4195-R) and 3+4 (primers BRV-1-10245-F and BRV-1-11804-R) in conserved regions used in the PCR reaction, see the section “Materials and Methods” and **Table 1**. M, GeneRuler Marker 1 kb DNA Ladder (Thermo Scientific Inc.); NTC, non-template control; Arrow, ARV-1. **(B)** Individual honey bees (B1, … , B10) and bumble bees (Bi1, … , Bi10) from U.S. samples. NTC, non-template control. Primers used 1+2 (BRV-1-2683-F and BRV-1-4195-R). Arrow, ARV-1.

We collected two additional pools of nurse honey bees and their associated *V. destructor* mites from two Israeli honey bee colonies and screened these for the presence of ARV-1 using RT-PCR. We utilized two different pairs of primers that anneal at different regions of the viral genome (nucleotides 2683–4195 and 10245–11804, **Figure 2A**, lanes BP1, VP1 and BP2, and VP2, respectively). This analysis clearly identified the presence of ARV-1 sequences in these pools (**Figure 2A**, lanes BP and VP, respectively).

Additionally, we analyzed the collected U.S. bee samples for the presence of ARV-1 using RT-PCR with the same primers. For the virome analysis, described above we evaluated pooled samples (10 bees), and the pools of field-collected bumble bees and colony-collected honey bees were positive for ARV-1. For the RT-PCR analysis, we examined the 10 individuals from each of these pooled samples. ARV-1 amplified in 7 of the 10 honey bees and 1 of 10 bumble bees (**Figure 2B**).

### Prevalence, Quantification, and Replication of ARV-1 in Individuals

To estimate the prevalence of ARV-1 in Israeli honey bee populations, we screened for its presence in colonies from apiaries located at the North, Center, and South of Israel (Dan, Kfar Ruth, and Yad Mordechai, respectively). We found that 21 of the 104 colonies analyzed were positive for the virus (**Table 2**).

**Table 2:** Prevalence of ARV-1 in Israeli apiaries.

Location	Number of	ARV-1	ARV-1
	colonies	positive	positive (%)
Dan	32	9	28.1
Kfar Ruth	34	9	26.5
Yad Mordechai	38	3	7.9


*Pools of 30 nurse bees were analyzed by RT-PCR for the presence or absence of ARV-1 using primers BRV-1-10245-F and BRV-1-11804-R.*

RT-PCR screening of small subsamples of individual nurse bees (*n* = 84) and *V. destructor* mites (*n* = 21) from these Israeli colonies demonstrated that the viruses were not present in all the individuals from the same colony. Thus, in random sampling of individual bees and mites from four ARV-1-positive colonies we found the virus in 4.8% of bees and in 76.2% of the mites evaluated. Moreover, the number of genomic copies of ARV-1 estimated by RT-qPCR was similar among bees and *V. destructor* mites collected from these colonies: 1.2 × 10^5^–7.3 × 10^7^ and 2.0 × 10^5^–4.5 × 10^8^, respectively (**Table 3**).

**Table 3:** Genomic copies of ARV-1 in individual nurse bees and mites.

ARV-1 copies	

Honey bees	Mites
3.0*E* + 07	3.8*E* + 08
7.3*E* + 07	5.9*E* + 07
4.8*E* + 06	2.5*E* + 08
1.9*E* + 07	3.4*E* + 08
2.1*E* + 07	2.4*E* + 07
1.2*E* + 07	1.1*E* + 07
8.7*E* + 06	8.5*E* + 07
3.9*E* + 05	2.0*E* + 05
5.2*E* + 07	2.9*E* + 08
8.5*E* + 05	5.3*E* + 07
2.5*E* + 07	2.0*E* + 07
1.4*E* + 05	1.1*E* + 08
1.5*E* + 07	5.2*E* + 07
7.7*E* + 05	2.2*E* + 07
2.0*E* + 07	1.9*E* + 07
4.0*E* + 07	6.3*E* + 05
3.2*E* + 06	2.1*E* + 07
6.2*E* + 05	2.3*E* + 07
6.1*E* + 05	4.5*E* + 08
1.2*E* + 05	8.3*E* + 05


*Each line indicates a separate individual honey bee or mite.*

**FIGURE 3:**
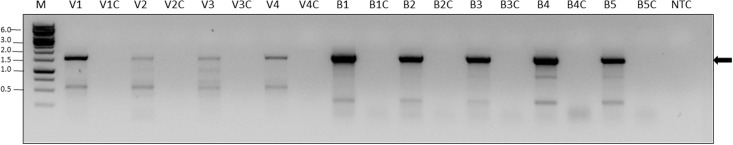
Detection of the ARV-1 positive-sense RNA strand in *V. destructor* (V) and *A. mellifera* (B). The numbers indicate the individual tested. Suffix C indicates PCR control reaction from the same individual RNA performed on cDNA produced without any primer (see the section “Materials and Methods” and **Table 1**). PCR primers: BRV-1F-10356-R and TAG-D_F. M, GeneRuler Marker 1 kb DNA Ladder (Thermo Scientific Inc.); NTC, non-template control; Arrow, ARV-1 amplicon that was confirmed by sequencing (see the section “Materials and Methods”).

To confirm replication of ARV-1 in our samples, we screened for the presence of the positive-sense RNA strand of the virus using RNA-strand sense-specific primer-tagged-RT-PCR (see the section “Materials and Methods”). A predicted size fragment of about 1650 nucleotides corresponding to the ARV-1 positive-sense-strand RNA size comprised between nucleotides 8711 and 10356 was found in all the tested samples from individual Varroa mites or nurse bees that showed ARV-1 copies in the above RT-qPCR assay (**Figure 3**, lanes V1, V2, …, etc. and B1, B2, …, etc.). No amplification was observed in control samples when PCR was performed with cDNA of the same individuals prepared from RNA without any oligonucleotide primer in the RT reaction (**Figure 3**, lanes V1C, V2C, …, etc. and B1C, B2C, …, etc.; and Replication assay in the section “Materials and Methods”).

Individuals nurse honey bees and *V. destructor* mites that were negative in the above RT-qPCR did not show amplification (not shown). Moreover, using Sanger DNA sequencing of the above specific-primer-tagged amplicons, we confirmed that it was identical to the ARV-1 sequence comprised between the nucleotides 8711 and 10356 of the viral genome.

## Discussion

Currently, there have been more than 20 viruses identified from *A. mellifera* honey bees around the world (McMenamin and Genersch, 2015). At least 11 of these viruses have been observed in other bee species (Tehel et al., 2016), and many of these viruses are also found in – and vectored by – *V. destructor* mites, a key parasite of *A. mellifera* honey bees. The vast majority of viruses identified in *A. mellifera* and found, thus far, to be circulating in other bee species and transmitted by *V. destructor* mites are positive-sense single-strand RNA viruses. Here, we found that ARV-1, a negative-sense single-strand RNA virus, that was recently found in honey bees and Varroa mites (Remnant et al., 2017), was present in two communities of bee pollinators.

Members of the *Rhabdoviridae* family of viruses typically infect animals and plants, but are mostly transmitted by arthropods (Kuzmin et al., 2009). Because viruses can be transmitted through pollen (Singh et al., 2010) and bees actively collect pollen, there is concern that viruses identified in bee samples may simply represent contamination from pollen. Indeed, in metagenomics studies of bee viruses, many viruses known to infect plants are often found (Schoonvaere et al., 2016). In our study, we demonstrated that ARV-1 is actively replicating in its *A. mellifera* honey bee and *V. destructor* mite hosts, by identifying the positive-sense RNA strand of the virus using RNA strand sense-specific-primer-tagged-RT-PCR, and confirming the product via Sanger sequencing. Furthermore, the fact that ARV-1 has been identified in *A. mellifera* populations from five distinct regions (Europe, North America, Middle East, Africa, and South Pacific) suggests that it is indeed infecting honey bee populations and is not simply a plant contaminant.

We identified ARV-1 in *A. mellifera* honey bee populations in 21 out of 104 colonies screened from three distinct locations in Israel. Although ARV-1 was found in ∼20% of the colonies screened, it was only present in 4.8% of the sampled bees and 76.2% of the mites collected from ARV-1-positive colonies (**Table 2**). These data suggest that this virus is not highly infectious. However, many factors – including age, physiological state, nutritional status, co-infection with other parasites and pathogens, and pesticide exposure – can influence the ability of a honey bee to tolerate or clear a viral infection (McMenamin et al., 2016). Thus, more detailed monitoring and experimental studies are needed to understand the infection dynamics of ARV-1.

While ARV-1 was detected in honey bees with deformed wings in the U.S. samples, there was no indication of any symptoms in the Israeli samples. It remains to be determined if ARV-1 causes symptoms in honey bees. It is known that bees can carry viruses asymptomatically and virulent infections can be induced by various stresses (Genersch and Aubert, 2010; Nazzi and Pennacchio, 2014). Other bee viruses such as LSV are not associated with any known symptom in individual bees (Daughenbaugh et al., 2015). Additional controlled studies are necessary to determine if ARV-1 infection causes any negative symptoms in its bee or mite hosts, under a wide range of ecologically relevant conditions. Moreover, some viruses are in mutualistic relationships with their hosts, and can improve their hosts’ resilience and fitness under certain conditions (Roossinck, 2011).

Remnant et al. (2017; Roossinck, 2011) named the rhabdoviruses they found ARV-1 and ARV-2, however, they were likely unaware that the ARV abbreviation they applied was previously attributed to another rhabdovirus Adelaide River virus (Wang et al., 1995), a fact that may create confusion while analyzing data. Furthermore, our results indicate that this virus is found in bumble bee species as well. Thus, we propose that these viruses should be called bee rhabdoviruses and abbreviated BRV-1 and BRV-2, instead of ARV-1 and -2, respectively.

In addition to BRV-1 and BRV-2, recent studies have identified several negative-sense RNA viruses: two putative Bunya-like virus of *A. mellifera*, *A. mellifera* bunyavirus-1 and -2 (ABV-1 and ABV-2, respectively; Remnant et al., 2017), Scaldis River bee virus (SRBV), and Ganda bee virus (GABV) in *Osmia cornuta* bees (Schoonvaere et al., 2016). The latter appear to belong the proposed new family *Chuviridae* of the order *Mononegavirales* and the new family *Phasmaviridae* of the order *Bunyavirales*.

With the advent of high-throughout sequencing, improved bioinformatics approaches, and expanded genomic databases, it is now possible to readily identify the microbial communities from field-collected populations of animals and plants. Bee viruses are particularly fascinating, as they can infect diverse species, are transmitted via multiple routes (contaminated flowers, social interactions, and vectors), and their pathogenicity and virulence are influenced by a number of biotic and abiotic factors. While previous studies have identified and focused on positive-sense single-strand RNA viruses in honey bees and the broader bee community, our results, together with those of Schoonvaere et al. (2016), Remnant et al. (2017), and those of Li et al. (2015) – who found numerous negative-sense RNA viruses by metagenomics analysis of 70 species of insects, spiders, centipedes, etc. in China – suggest that negative-sense strand RNA viruses may also be circulating and broadly distributed among honey bee populations and bee communities. It remains to be determined if these negative-sense RNA viruses are functionally distinct from positive-sense RNA viruses in terms of their pathogenicity and virulence.

## Author Contributions

NC, SL, NS, TE, DG, and CG conceived and designed the experiments. SL, TE, and DG performed the experiments. NS, SL, and DG bioinformatic analysis. NC, SL, DG, and CG data analysis. NC, NS, SL, TE, DG, and CG wrote the paper and prepared the figures and tables. NC, DG, and CG edited the manuscript. NC, NS, SL, TE, DC, and CG revised and approved the manuscript.

## Conflict of Interest Statement

The authors declare that the research was conducted in the absence of any commercial or financial relationships that could be construed as a potential conflict of interest. The reviewer PW and handling Editor declared their shared affiliation.

## Footnotes

1 http://multalin.toulouse.inra.fr/multalin/
