# Supplementary material for: Presence of Apis Rhabdovirus-1 in Populations of Pollinators and Their Parasites from Two Continents
Source: Front Microbiol. 2017 Dec 12;8:2482. doi: 10.3389/fmicb.2017.02482 (PMC5732965; doi:10.3389/fmicb.2017.02482)
Supplement: Supplementary file 1 [file Data_Sheet_1.docx]

Supplementary Material

Presence of Apis rhabdovirus-1 in populations of pollinators and their parasites from two continents.

**Sofia Levin**^1,2^**, David Galbraith**^3^**, Noa Sela**^4^**, Tal Erez**^1^**, Christina M. Grozinger**^3^ **and Nor Chejanovsky**^1,5^**^*^**

^1^Entomology Department, Institute of Plant Protection, Agricultural Research Organization, Israel

^2^Faculty of Agricultural, Food and the Environmental Quality Sciences, The Hebrew University of Jerusalem, Rehovot, Israel

^3^Department of Entomology, Center for Pollinator Research, Huck Institutes of the Life Sciences, Pennsylvania State University, University Park, PA

^4^ Department of Plant Pathology and Weed Research, Institute of Plant Protection, Agricultural Research Organization, Israel

^5^Institute of Bee Health, Vetsuisse, University of Bern, Switzerland

*** Correspondence:**Nor Chejanovsky
ninar@volcani.agri.gov.il

**SUPPLEMENTARY TABLE S1.** Primers used for amplification, sequencing and quantitation of Apis rhabdovirus-1 (ARV-1)

| **Name** | **Sequence** | **Application** |  |
| --- | --- | --- | --- |
| BRV-2683-F | GGAACGGTGACTCCTTCGTT | sequencing, diagnostics | |
| BRV-4195-R | TGAGCCATCGTTGCGTTTTG | sequencing, diagnostics | |
| BRV-10245-F | TGCAGTAGAATGGCTGCACA | sequencing, diagnostics | |
| BRV-11804-R | CTGTGGTAACCTGGGCAACA | sequencing, diagnostics | |
| BRV-4148-F | TCCCCGAACGCATACCACA | sequencing | |
| BRV- 5838-R | CCAGGGGTCCCTAGGGTTTA | sequencing | |
| BRV-8711-F | TCAAGCTAGATCGCCAGGTA | Sequencing | |
| BRV-10356-R | GACGACCGTCTTCTTTCGCA | Sequencing, replication | |
| BRV-qRT-F1 (10445-F) | ACCCTTTTCGCCTTACCTCG | qPCR | |
| BRV-qRT-R1 (10627-R)  BRV 8711F-TAG  TAG-D F  RPL8 F (honey bee, housekeeping)  RPL8 R (honey bee, housekeeping) | GTCACTGATTGAGCTAGCCGT  AGCCTGCGCACCGTGGTCAAGCTAGATCGCCAGGTA  AGCCTGCGCACCGTGG  TGGATGTTCAACAGGGTTCATA  CTGGTGGTGGACGTATTGATAA | qPCR  Replication  (cDNA)  Replication  qPCR  qPCR | |
| VcytoactinF (Varroa) | AAGTCGTACGAGCTTCCCGAC | qPCR | |
| VcytoactinR (Varroa) | ACAGGGAGGCAAGGATGGAAC | qPCR | |

Suffix F and R indicate orientations forward and reverse in respect to the viral genome.

1 50

RV_Am_IL GACGCTACAA CAGACCAAAA ATAG...... .......... ......TATC

RV_Bi_US ..CGCTACAA CAGACCAAAA ATAG...... .......... ......TATC

RV_Am_US GACGCTACAA CAGACCAAAA ATAG...... .......... ......TATC

RV_Vd_IL ........AA CAGACCAAAA ATGCATCTTC GTTTCTGTAA AATATTTATA

Consensus ..cgctacAA CAGACCAAAA ATag...... .......... ......TATc

51 100

RV_Am_IL AAACCATCTT TTGTCTGGGG TTCTTACTGG GGGGAACGGT CAAGCCGTTA

RV_Bi_US AAACCATCTT TTGTCTGGGG TTCTTACTGG GGGGAACGGT CAAGCCGTTA

RV_Am_US AAACCATCTT TTGTCTGGGG TTCTTACTGG GGGGAACGGT CAAGCCGTTA

RV_Vd_IL GATGACTGAT GTGACTTGGT ACACTAGTAT CGGGCCATTT TTTGTCATTT

Consensus aAaccaTctT tTGtCTgGGg ttctTAcTgg gGGGaacggT caaGcCgTTa

101 150

RV_Am_IL GGAAATGTTT TTTCGTGGAT TGATGTACCG TCC....... ......GGAC

RV_Bi_US GGAAATGTTT TTTCGTGGAT TGATGTACCG TCC....... ......GGAC

RV_Am_US GGAAATGTTT TTTCGTGGAT TGATGTACCG TCC....... ......GGAC

RV_Vd_IL ATATCTATTG CTAATTGCCG TTTGAATCCG GCTATAACAC CAACGGGCAC

Consensus ggAaaTgTTt tTtcgTGgat TgatgtaCCG tCc....... ......GgAC

151 200

RV_Am_IL GCTTTGCTTC TTCATGCCTT TGGAAGATTG AGCAAGAAAC TTTAGAACCA

RV_Bi_US GCTTTGCTTC TTCATGCCTT TGGAAGATTG AGCAAGAAAC TTTAGAACCA

RV_Am_US GCTTTGCTTC TTCATGCCTT TGGAAGATTG AGCAAGAAAC TTTAGAACCA

RV_Vd_IL ACCATTCGCC TTTGTATCGA TCTAATTTGC CGTCAATCCC TTTTCAAAAA

Consensus gCttTgCttC TTcaTgcCtt TggAAgaTtg aGcaAgaaaC TTTagAAccA

201 250

RV_Am_IL GTATTAACT. .......... .......... ....CTTATT TAAAAAGGTC

RV_Bi_US GTATTAACT. .......... .......... ....CTTATT TAAAAAGGTC

RV_Am_US GTATTAACT. .......... .......... ....CTTATT TAAAAAGGTC

RV_Vd_IL TCATTGACTG CCTTGAGACC GAAACGATCT GGGACCGATT TAGACTTGTC

Consensus gtATTaACT. .......... .......... ....CttATT TAaAaagGTC

251 300

RV_Am_IL TATTCATTGA GATTAATCTG TTTAGTCTTT TTATAACTTT TATACTTAAT

RV_Bi_US TATTCATTGA GATTAATCTG TTTAGTCTTT TTATAACTTT TATACTTAAT

RV_Am_US TATTCATTGA GATTAATCTG TTTAGTCTTT TTATAACTTT TATACTTAAT

RV_Vd_IL TTGTAAGTCC AAAAGATCTG GATTATCCAG CCGGACCTGT GAATCTGGAA

Consensus TatTcAtTga gAttaATCTG ttTagTCttt ttatAaCTtT tAtaCTtaAt

301 350

RV_Am_IL ATTGAGAACA TTTAGCACAT TGACACCAGT CAGTATCTTT AAAGCAATAA

RV_Bi_US ATTGAGAACA TTTAGCACAT TGACACCAGT CAGTATTTTT AAAGCAATAA

RV_Am_US ATTGAGAACA TTTAGCACAT TGACACCAGT CAGTATCTTT AAAGCAATAA

RV_Vd_IL TAAACGATTA GACCGATTGA AATTAACATT TTGTTTTTTT AATAATGAGT

Consensus attgaGAacA tttaGcacat tgacAcCAgT caGTaTcTTT AAagcaataa

351 400

RV_Am_IL TGGCCAGCCT TTCGAACTCG CAAATTCAGG CGCT...... ..........

RV_Bi_US TGGCCAGCCT TTCGAACTCG CAAATTCAGG CGCT...... ..........

RV_Am_US TGGCCAGCCT TTCGAACTCG CAAATTCAGG CGCT...... ..........

RV_Vd_IL GGTCTTCCCT TGTGGTCTGG GCGTTGAGCA TGTTGATTGC TCTCTGTTTG

Consensus tGgCcagCCT TtcGaaCTcG caaaTtcagg cGcT...... ..........

401 450

RV_Am_IL ..CGCAAAAC GTTTAGGGAA GCTCTCCGCA TCTGAAACAC AACCGACCCC

RV_Bi_US ..CGCAAAAC GTTTAGGGAA GCTCTCCGCA TCTGAAACAC AACCGACCCC

RV_Am_US ..CGCAAAAC GTTTAGGGAA GCTCTCCGCA TCTGAAACAC AACCGACCCC

RV_Vd_IL AGCTGTATTC ACTTGGGGAA AATGTGTGAA TG....ATTC TCCTGGACAG

Consensus ..CgcaAaaC gtTTaGGGAA gcTcTccGcA TctgaaAcaC aaCcGacCcc

451 500

RV_Am_IL GAAAAACTTT GAGGTTGACT CGTACCTTTC GATTCCCTTT ATTTGTCATG

RV_Bi_US GAAAAACTTT GAGGTTGACT CGTACCTTTC GATTCCCTTT ATTTGTCATG

RV_Am_US GAAAAACTTT GAGGTTGACT CGTACCTTTC GATTCCCTTT ATTTGTCATG

RV_Vd_IL AAAACTCATT GAAATATTTG GAACTTTTTC GAACATTGTT TTCCGGCTCG

Consensus gAAAaaCtTT GAggTtgact cgtaccTTTC GAttccctTT aTttGtCatG

501 550

RV_Am_IL TTGCACCAAC AATTTCCATC GTACATTTAT .TAACTGCAG TAACTGAAGG

RV_Bi_US TTGCACCAAC AATTTCCATC GTACATTTAT .TAACTGCAG TAACTGAAGG

RV_Am_US TTGCACCAAC AATTTCCATC GTACATTTAT .TAACTGCAG TAACTGAAGG

RV_Vd_IL TTGATCCACA GACCCCTTAC AGACTGCCCT GTCGCTGCAG CCAAGTATTT

Consensus TTGcaCCAac aAtttCcatC gtACatttaT .TaaCTGCAG taActgAagg

551 600

RV_Am_IL TCTAGCATC. .......... ...AACGTAT AGAGTGGCGC AAGCTTTATT

RV_Bi_US TCTAGCATC. .......... ...AACGTAT AGAGTGGCGC AAGCTTTATT

RV_Am_US TCTAGCATC. .......... ...AACGTAT AGAGTGGCGC AAGCTTTATT

RV_Vd_IL TGGAGCTTTC CTGAGGACAC GGTATCTTTG TGAATTGCCC ATATTGAGAT

Consensus TctAGCaTc. .......... ...AaCgTat aGAgTgGCgC AagcTttatT

601 650

RV_Am_IL TGTTGAATCC GTCAAATCCA AAATCGGGAC AATTGAGAAA GGTCCGACAT

RV_Bi_US TGTTGAATCC GTCAAATCCA AAATCGGGAC AATTGAGAAA GGTCCGACAT

RV_Am_US TGTTGAATCC GTCAAATCCA AAATCGGGAC AATTGAGAAA GGTCCGACAT

RV_Vd_IL TATTGGAGCT GTAACAGCCA C..TCAGAGC ACTGCTGTGT CTCCAAGCCT

Consensus TgTTGaAtCc GTcAaAtCCA aaaTCgGgaC AaTtgaGaaa ggtCcgaCaT

651 700

RV_Am_IL CAATGACAAT TGCATCTTTT ACGCCTCCAG TTCCTACTCA GGAAGTAATA

RV_Bi_US CAATGACAAT TGCATCTTTT ACGCCTCCAG TTCCTACTCA GGAAGTAATA

RV_Am_US CAATGACAAT TGCATCTTTT ACGCCTCCAG TTCCTACTCA GGAAGTAATA

RV_Vd_IL CATTAACAAA AAAAACTTAT TATAAGTTTA GGGCTTAACG GTAATGAGGT

Consensus CAaTgACAAt tgcAtCTTtT acgcctccag ttcCTactCa GgAAgtAata

701 750

RV_Am_IL GCATGGGTCC GAGAGAATCC GGAACACTTA ACAGAAGATT GGATACTTGC

RV_Bi_US GCATGGGTCC GAGAGAATCC GGAACACTTA ACAGAAGATT GGATACTTGC

RV_Am_US GCATGGGTCC NAGAGAATCC GGAACACTTA ACAGAAGATT GGATACTTGC

RV_Vd_IL ATAGGGATC. ..GTTAATTG CTTACGCGTG AAAGAAGATC TCAAATGATG

Consensus gcAtGGgTCc gaGagAATcc ggaACaCtTa AcAGAAGATt ggAtActtgc

751 800

RV_Am_IL GTTTGCTGCG TACCTTGTCG GCTGGTCCAT GCGGAAGACG GGTAGAGCTA

RV_Bi_US GTTTGCTGCG TACCTTGTCG GCTGGTCCAT GCGGAAGACG GGTAGAGCTA

RV_Am_US GTTTGCTGCG TACCTTGTCG GCTGGTCCAT GCGGAAGACG GGTAGAGCTA

RV_Vd_IL GATCTCACAG GATAATCAAC TTTGGAAACA GCTATCTGTA TAAAGTGGGA

Consensus GtTtgCtgcG tAcctTgtcg gcTGGtccat GCggaagacg ggtAGaGctA

801 850

RV_Am_IL ACCAAATAGG CAAGTTCTGT GGAAATATAG AA.AAGATAC TAAATATCTC

RV_Bi_US ACCAAATAGG CAAGTTCTGT GGAAATATAG AA.AAGATAC TAAATATCTC

RV_Am_US ACCAAATAGG CAAGTTCTGT GGAAATATAG AA.AAGATAC TAAATATCTC

RV_Vd_IL AAGATATCGA TACAACTTTT TTAGTTAACA AAGAAGAATA TTAAATTGAA

Consensus AccAaATaGg cAagttcTgT ggAaaTAtag AA.AAGAtac TaAAtaTctc

851 900

RV_Am_IL TGGACCTACT TTATCTCCTG AGATTATTAA TGGCATGCTG AGATTACCCC

RV_Bi_US TGGACCTACT TTATCTCCTG AGATTATTAA TGGCATGCTG AGATTACCCC

RV_Am_US TGGACCTACT TTATCTCCTG AGATTATTAA TGGCATGCTG AGATTACCCC

RV_Vd_IL GATTACTCAT TAAACTCTCA AGTCAAGCCA TTGGAGACAC CGACAAAGGT

Consensus tggacCTacT TtAtCTCctg AGattAttaA TgGcAtgCtg aGAttAcccc

901 950

RV_Am_IL AGATTAGCTC TACAGATTAT GATATACATT C.....TACT CTGACACATT

RV_Bi_US AGATTAGCTC TACAGATTAT GATATACATT C.....TACT CTGACACATT

RV_Am_US AGATTAGCTC TACAGATTAT GATATACATT C.....TACT CTGACACATT

RV_Vd_IL GTAGTTGGTT TTGAGATGCT TCAACGTAGT CCCGGTTAAT CTGTCACTTC

Consensus agAtTaGcTc TacAGATtaT gatAtacAtT C.....TAcT CTGaCACaTt

951 1000

RV_Am_IL CGCTTTTACT ACTAGACAAT ACT...GACG TACCTCACTG GGAAGCTATG

RV_Bi_US CGCTTTTACT ACTAGACAAT ACT...GACG TACCTCACTG GGAAGCTATG

RV_Am_US CGCTTTTACT ACTAGACAAT ACT...GACG TACCTCACTG GGAAGCTATG

RV_Vd_IL AGCTTTCTGG AGATGTTAGG ACACGAGAGG AACAGGCCAG GTTGGTAGTT

Consensus cGCTTTtact ActaGacAat ACt...GAcG tACctcaCtG GgaaGctaTg

1001 1050

RV_Am_IL ATGTTTTATG CCTTATATCG CCCGATCTGT TCTCAATCAA CTGTACTTTC

RV_Bi_US ATGTTTTATG CCTTATATCG CCCGATCTGT TCTCAATCAA CTGTACTTTC

RV_Am_US ATGTTTTATG CCTTATATCG CCCGATCTGT TCTCAATCAA CTGTACTTTC

RV_Vd_IL TTGTTGTAAT CTCTAGAAGT GAAGTGAATT TAGGCTGAAG CATTAGTTGG

Consensus aTGTTtTAtg CctTAtAtcg cccGatctgT TctcaatcAa CtgTAcTTtc

1051 1100

RV_Am_IL GAGAAGATAT GCCGAATTTA CAAAATTACT ......TCCT ACAGCTTTTG

RV_Bi_US GAGGAGATAT GCCGAATTTA CAAAATTACT ......TCCT ACAGCTTTTG

RV_Am_US GAGGAGATAT GCCGAATTTA CAAAATTACT ......TCCT ACAGCTTTTG

RV_Vd_IL TTTAAGATGT GTTCTCTCCA AGGAAATGGT AGAATGTTGT CAACCTATTT

Consensus gagaAGATaT GccgaaTttA caaAAtTacT ......TccT acAgCTtTTg

1101 1150

RV_Am_IL AGAGTGAAAA TTATTTGA.. ......CTAA TGGTGATTTT AGGGCAGGGA

RV_Bi_US AGAGTGAAAA TTATTTGA.. ......CTAA TGGTGATTTT AAGGCAGGGA

RV_Am_US AGAGTGAAAA TTATTTGA.. ......CTAA TGGTGATTTT AAGGCAGGGA

RV_Vd_IL AGGGTTAGAT TTTTGGTAAC GAATCTCTAA TGAGATCTCT TCGGTTCTTT

Consensus AGaGTgAaAa TTaTttgA.. ......CTAA TGgtgatTtT aaGGcaggga

1151 1200

RV_Am_IL TGATTGCCCT ...TTTGGAG TACTGGGGAG CGATAGAAAA ATGCAACCTA

RV_Bi_US TGATTGCCCT ...TTTGGAG TACTGGGGAG CGATAGAAAA ATGCAACCTA

RV_Am_US TGATTGCCCT ...TTTGGAG TACTGGGGAG CGATAGAAAA ATGCAACCTA

RV_Vd_IL TGTTTTCCCA ATCTTTTGTG ATTTAAGTTT AGAAAGCATT TTTAAAGTTT

Consensus TGaTTgCCCt ...TTTgGaG tacTggGgag cGAtAGaAaa aTgcAAccTa

1201 1250

RV_Am_IL TCAGGGAAAA CAAAACACCA GCCTATGGTG ACTCGAACCC TGGACACAG.

RV_Bi_US TCAGGGAAAA CAAAACACCA GCCTATGGTG ACTCGAACCC TGGATACAG.

RV_Am_US TCAGGGAAAA CAAAACACCA GCCTATGGTG ACTCGAACCC TGGATACAG.

RV_Vd_IL TTAGAAAAAA TGTTTAAACA TCTTCTTCAT CACTTTCCCC TTGGTTCTCA

Consensus TcAGggAAAA caaaacAcCA gCcTaTggtg actcgaaCCC TgGataCag.

1251 1300

RV_Am_IL .......... .......... ...GTTTCTT CTCCGATCTG TCCAGAATGG

RV_Bi_US .......... .......... ...GTTTCTT CTCCGATCTG TCCAGAATGG

RV_Am_US .......... .......... ...GTTTCTT CTCCGATCTG TCCAGAATGG

RV_Vd_IL TCATTGGGAT CTTCGTCTGA ATAGTTTAAG CTCAGATTTG TATTCATT..

Consensus .......... .......... ...GTTTctt CTCcGATcTG TccagAaTgg

1301 1350

RV_Am_IL AATCGAAAAG AATGTCTGCA GCACTTCTAT ATTGCATCTG CTTAAAAAGC

RV_Bi_US AATCGAAAAG AATGTCTGCA GCACTTCTNT ATTGCATCTG NTTAAAAAGC

RV_Am_US AATCGAAAAG AATGTCTGCA GCACTTCTAT ATTGCATCTG CTTAAAAAGC

RV_Vd_IL ..TCTTGATT AAAACCAGCT TCACCAGGAT AGTATGTACG AATGACAGAA

Consensus aaTCgaaAag AAtgtCtGCa gCACttctaT AtTgcaTctG ctTaAaAagc

1351 1400

RV_Am_IL GATAGAATGG CTGATTTTGA AGCATTT... ......AAAT CAGCGGGATA

RV_Bi_US GATAGAATGG CTGATTTTGA AGCATTT... ......AAAT CAGCGGGATA

RV_Am_US GATAGAATGG CTGATTTTGA AGCATTT... ......AAAT CAGCGGGATA

RV_Vd_IL GGTAAAAATA TACAGTTATT ATCTCCTCCT GGGAAGAAAA AAGTGAAGCT

Consensus GaTAgAAtgg ctgAtTTtga AgCattT... ......AAAt cAGcGggata

1401 1450

RV_Am_IL TTCAGCAACA GTACACCAAG CGGCAGATGA AGTCCATTTA AAGCGGTGGG

RV_Bi_US TTCAGCAACA GTACACCAAG CGGCAGATGA AGTCCATTTA AAGCGGTGGG

RV_Am_US TTCAGCAACA GTACACCAAG CGGCAGATGA AGTCCATTTA AAGCGGTGGG

RV_Vd_IL GTCATCAATC ATCAACCACC TACCCTTGCT TCTAAGTTTA AAAGGATCTA

Consensus tTCAgCAAca gTacACCAag cggCagatga agTccaTTTA AAgcGgTggg

1451 1500

RV_Am_IL TAGAGAAGAT GTCAAGTGAG GACTCTGAAA GAAAGTTTGC CGGATTTTTT

RV_Bi_US TAGAGAAGAT GTCAAGTGAA GACTCTGAAA GAAAGTTTGC CGGATTTTTT

RV_Am_US TAGAGAAGAT GTCAAGTGAA GACTCTGAAA GAAAGTTTGC CGGATTTTTT

RV_Vd_IL CAGTAACTGT AGTCGGTGAA AAGGAGCTTA GCTTAACGTG ATGCTTTTCC

Consensus tAGagAagaT gtcaaGTGAa gActctgaaA Gaaagtttgc cgGaTTTTtt

1501 1550

RV_Am_IL GATGACGAAC AGGAAGCTGA CATTGAGTCC TTGCTGAAAG TTTAACATGA

RV_Bi_US GATGACGAAC AGGAAGCTGA CATTGAGTCC TTGCTGAAAG TTTAACATGA

RV_Am_US GATGACGAAC AGGAAGCTGA CATTGAGTCC TTGCTGAAAG TTTAACATGA

RV_Vd_IL AATAACAAGG CACCAATTAT TCTATATGCC TTCTTAATTA TTAAACCTTC

Consensus gATgACgAac aggaAgcTga caTtgAgtCC TTgcTgAaag TTtAACaTga

1551 1600

RV_Am_IL AACACCAAGC TGAAGTCACC CCAAGACCCC CATGAACACC CCAAATCCAC

RV_Bi_US AACACCAAGC NGAAGTCACC CCAAGACCCC CATGAACACC CCAAATCCAC

RV_Am_US AACACCAAGC NGAAGTCACC CCAAGACCCC CATGAACACC CCAAATCCAC

RV_Vd_IL CTTACCTTTA TATAACGGAA CTATCCAAGT GTTATGATTC TTTTTTGTTC

Consensus aacACCaagc ngaAgtcacc CcAagacccc caTgaacacC ccaaaTccaC

1601 1650

RV_Am_IL CCTGATCTCC GAAACTCCCC AAGTCAGCAG ..CCGGACAT CAAAGCAACC

RV_Bi_US CCTGATCTCC GAAACTCCCC AAGTCAGCAG ..CCGGACAT CAAAGCAACC

RV_Am_US CCTGATCTCC GAAACTCCCC AAGTCAGCAG ..CCGGACAT CAAAGCAACC

RV_Vd_IL CCTTTTCAAT AGCAAAACTC AGGTAACCTT TCCCTTTTAT TAAACTAACT

Consensus CCTgaTCtcc gaaActcCcC AaGTcAgCag ..CCggacAT cAAAgcAACc

1651 1700

RV_Am_IL AGAGCAAC.. .CCCAAACCA CTCAGAACCA AACTCACTAT AAAAAATACA

RV_Bi_US AGAGCAAC.. .CCCAAACCA CTCAGAACCA ACCTCACTAT AAAAAATACA

RV_Am_US AGAGCAAC.. .CCCAAACCA CTCAGAACCA ACCTCACTAT AAAAAATACA

RV_Vd_IL TTAGCAACTA TCCGAGTTGC GATTTTATCT ATTTTCCTTA TAGATTCAGA

Consensus agAGCAAC.. .CCcAaacca ctcagaAcCa AccTcaCTat aAaAaatAcA

1701 1750

RV_Am_IL TATCCCCTCT TTCTCAACA. .........A CAACTTAAGC TCCGCAGCTC

RV_Bi_US TATCTTTTCT TTCTCAACA. .........A TAACTTAAGC TCCGCAGCTC

RV_Am_US TATCTTTTCT TTCTCAACA. .........A TAACTTAAGC TCCGCAGCTC

RV_Vd_IL TACTTCCACT GGAGCAATAG ATATAAGAGA TAGAAAAAGG TTTGCAGCAG

Consensus TAtctcctCT ttctCAAcA. .........A tAacttAAGc TccGCAGCtc

1751 1800

RV_Am_IL TCAATCTCTC AACCAGAAGA AGCAGCTCCA TCTGACCCCA GTC.......

RV_Bi_US TCAATCTCTC AACCAGAAGA AGCAGCTCCA TCTGACCCTA GTC.......

RV_Am_US TCAATCTCTC AACCAGAAGA AGCAGCTCCA TCTGACCCTA GTC.......

RV_Vd_IL TGTATATAAT TTTCCTAATT TTTTGTTCCT TTAATATCTT ATTCTTTTTC

Consensus TcaATcTctc aacCagAAga agcaGcTCCa TctgaccCta gTc.......

1801 1850

RV_Am_IL ..AACACACT CCGATTCAGC AATCAACCAC ACTCAAACTC AAACATCCCG

RV_Bi_US ..AACACACT CCGATTTAGC AATCAACTAC ACTCAAACTC AAACACCCCG

RV_Am_US ..AACACACT CCGATTTAGC AATCAACTAC ACCCAAACTC AAACACCCCG

RV_Vd_IL TTAACACAAC CCCAATTACC TAAAGTTTTG GTGAAGTTGA TAATGGTCTT

Consensus ..AACACAct CCgAtTtAgC aAtcaactac actcAaactc aAAcaccCcg

1851 1900

RV_Am_IL TTCAAA.AGT CCCGCAACCG CTGCACGCAC CACCGGAACA CA.CAGATAG

RV_Bi_US TTCAAACAGT CCTGCAACCG CTGCACGCAC CACCGGAACA CA.TAGATAG

RV_Am_US TTCAAACAGT CCTGCAACCG CTGCACGCAC CACCGGAACA CA.TAGATAG

RV_Vd_IL CTCTAAAAGA CATGCAACTA GTCTAGGTAT GTCCAGCTCT AAGTTGCTGA

Consensus tTCaAAcAGt CctGCAACcg cTgcAcGcAc caCCgGaaCa cA.taGaTag

1901 1950

RV_Am_IL GTGCACACCC CTGCACCCTA GAAAGGTCAA AAACGCTCCA AGTCAAAGAA

RV_Bi_US GTGCATACTT CTGCACTTTA GAAAGGTTAA AAACGCTCCA AGTCAAAGAA

RV_Am_US GTGCATACTT CTGCACTTTA GAAAGGTTAA AAACGCTCCA AGTCAAAGAA

RV_Vd_IL CTAGTCCTTT CAGCTCATTA GGCAGTTCCA TACTTAGAGC ATCATAACAA

Consensus gTgcacactt CtGCaCttTA GaaAGgTcaA aAacgctcca AgtcaAAgAA

1951 2000

RV_Am_IL GTCCCTCAAA ACCCAATAGA AGCCAAAATG AGTGACCTAC TTA....AAA

RV_Bi_US GTCCCTTAAA ACTTAATAGA AGCCAAAATG AGTGACCTAC TTA....AAA

RV_Am_US GTCCCTTAAA ACTTAATAGA AGCCAAAATG AGTGACCTAC TTA....AAA

RV_Vd_IL TTGTCTTCTA AGTCATATAC TTCAAGAACT GCTTTTCTGA ATCTGCTAAT

Consensus gTccCTtaaA ActcAataga agCcAaAAtg agTgacCTac tTa....AAa

2001 2050

RV_Am_IL AATTCAATAA AGCCTCTAAA TCCGGACTGG CATATGAGTT TGAAAACGCA

RV_Bi_US AATTCAATAA AGCCTCTAAA TCCGGACTGG CATATGAGTT TGAAAACGCA

RV_Am_US AATTCAATAA AGCCTCTAAA TCCGGACTGG CATATGAGTT TGAAAACGCA

RV_Vd_IL TACGCATTCA CACGGCCATA ATGCACTGGC CCGAAGTGCC TTGTTAAGTG

Consensus aAttCAaTaA agCctCtAaA tccggactGg CatAtGaGtt TgaaaAcGca

2051 2100

RV_Am_IL GCATCATTAG ACGATGACAA CCTTGACATA AACACCAACG CAGTTGACTG

RV_Bi_US GCATCATTAG ACGATGACAA CCTTGACATA GACACCAACG CAGTTGACTG

RV_Am_US GCATCATTAG ACGATGACAA CCTTGACATA GACACCAACG CAGTTGACTG

RV_Vd_IL ACTGTTCTGG ATTATCT.AA TTTAATCATT GAGGCTTTGT CAGTATCTTG

Consensus gCatcatTaG AcgATgacAA ccTtgaCATa gAcaCcaacg CAGTtgacTG

2101 2150

RV_Am_IL GACTATGGAC CACGAAGATA ACATTACATT GGATAATATG GCCGAAGACA

RV_Bi_US GACTATGGAC CACGAAGATA ACATTACATT GGATAATATG GCCGAAGACA

RV_Am_US GACTATGGAC CACGAAGATA ACATTACATT GGATAATATG GCCGAAGACA

RV_Vd_IL TCCGATATTT CTTAAACATA TTAGAAACAT CTCTGTATTA CTGTTAGAAA

Consensus gaCtATggac CacgAAgATA acAttAcatT ggaTaataTg gccgaAGAcA

2151 2200

RV_Am_IL CCGGGCCAAA GGGTCTTCCG CTACACACTA AAACAAAGGA CGAGTTAACA

RV_Bi_US CCGGGCCAAA GGGTCTTCCG CTACACACTA AAACAAAGGA CGAGTTAACA

RV_Am_US CCGGGCCAAA GGGTCTTCCG CTACACACTA AAACAAAGGA CGAGTTAACA

RV_Vd_IL ACTTGGAGCC TTTTATCAAC GGAGACCAAC CTATATTTGA GACCCTAGTT

Consensus cCggGccaaa gggTcTtccg ctAcACacta aaAcAaagGA cgagtTAaca

2201 2250

RV_Am_IL TTGACTGGTT TTTTAATGTC TCAGTCCAAA GTAAACATAA TTAAAGCAAT

RV_Bi_US TTGACTGGTT TTTTAATGTC TCAGTCCAAA GTAAACATAA TTAAAGCAAT

RV_Am_US TTGACTGGTT TTTTAATGTC TCAGTCCAAA GTAAACATAA TTAAAGCAAT

RV_Vd_IL AAGATCTGTT TTAATCTATC GCAGGTGATA TTATACATTT TAACACTTAA

Consensus ttGActgGTT TTttaaTgTC tCAGtccAaA gTAaACATaa TtAaAgcaAt

2251 2300

RV_Am_IL GGGACAAATG CTTACAGCTA AATCTGTTTT TTATAAGACA AAAAATTGTG

RV_Bi_US GGGACAAATG CTTACAGCTA AATCTGTTTT TTATAAGACA AAAAATTGTG

RV_Am_US GGGACAAATG CTTACAGCTA AATCTGTTTT TTATAAGACA AAAAATTGTG

RV_Vd_IL GAACCTAAGA CTTTGAAGT. ......TTCG TCATTAAACA AAAAAGA...

Consensus GggaCaAAtg CTTacAgcTa aatctgTTtt TtATaAgACA AAAAAttgtg

2301 2350

RV_Am_IL ATTTTATCGA CGGCCTCATA GAAGGTTACA GACTCGCTGA AAGTGTTATA

RV_Bi_US ATTTTATCGA CGGCCTCATA GAAGGTTACA GACTCGCTGA AAGTGTTATA

RV_Am_US ATTTTATCGA CGGCCTCATA GAAGGTTACA GACTCGCTGA AAGTGTTATA

RV_Vd_IL ...TTTTCTA GAGCCTGATC CGTCGAATCT GGTTTCGTCC ATATATCACC

Consensus attTTaTCgA cgGCCTcATa gaagGttaCa GacTcgcTga AagTgTtAta

2351 2400

RV_Am_IL ACTGCAGAAT TATATAATAG TGACCACAGA TTGC...TTT CGGAAATCAA

RV_Bi_US ACTGCAGAAT TATATAATAG TGACCACAGA TTGC...TTT CGGAAATCAA

RV_Am_US ACTGCAGAAT TATATAATAG TGACCACAGA TTGC...TTT CGGAAATCAA

RV_Vd_IL CTCTGCATCT GAGATAATTC CGACCACCTG ATTGGAATTC AGTGAGGAAA

Consensus actgcagaaT tAtATAATag tGACCACaga tTgc...TTt cGgaAatcAA

2401 2450

RV_Am_IL ATCAGAAATG GTTGAAATGA AAAGAGCTCA AGAAAATTTA TCAGATACCT

RV_Bi_US ATCAGAAATG GTTGAAATGA AAAGAGCTCA AGAAAATTTA TCAGATACCT

RV_Am_US ATCAGAAATG GTTGAAATGA AAAGAGCTCA AGAAAATTTA TCAGATACCT

RV_Vd_IL TCCAGGACAT ACTGAAATCC GGATGAGTCA AGTCGTTAAT AAATCCAACA

Consensus atCAGaAatg gtTGAAATga aaAgagcTCA AGaaaaTtta tcAgatAcCt

2451 2500

RV_Am_IL TAGATCAAGT AGCCTCTTCA GTTCAATTGA TCGTTGCTGA ACATGATACC

RV_Bi_US TAGATCAAGT AGCCTCTTCA GTTCAATTGA TTGTTGCTGA ACATGATACC

RV_Am_US TAGATCAAGT AGCCTCTTCA GTTCAATTGA TTGTTGCTGA ACATGATACC

RV_Vd_IL AACTCCCCAC AGTTATGAGG TTTCAGCTGC TCTTTTAAGT ATGAATAAGC

Consensus tAgatCaagt AGcctcttca gTTCAatTGa TcgTTgctGa AcatgatAcC

2501 2550

RV_Am_IL AAAATGACCC AATTAGAATC GGCCTATAAG TCCGTGAAAG CGGAGAAAAC

RV_Bi_US AAAATGACCC AATTAGAATC GGCCTATAAG TCCGTAAAAG CGGAGAAAAC

RV_Am_US AAAATGACCC AATTAGAATC GGCCTATAAG TCCGTAAAAG CGGAGAAAAC

RV_Vd_IL AGAAGGAACT ATTGTTCCTA ACGC....AT TTTGTGTAAT TTGATCAAAA

Consensus AaAAtGAcCc AaTtagaaTc ggcCtataAg TccGTaaAAg cgGAgaAAAc

2551 2600

RV_Am_IL TCCTTTTGCA ATGTTCAAGG AAAATTATTC TGAAGAAGCT GGACCAAGAC

RV_Bi_US TCCTTTTGCA ATGTTCAAGG AAAATTATTC TGAAGAAGCT GGACCAAGAC

RV_Am_US TCCTTTTGCA ATGTTCAAGG AAAATTATTC TGAAGAAGCT GGACCAAGAC

RV_Vd_IL GAAATAAGTG TTTGATAGAG AACCTTCTTC CCCA...... ..........

Consensus tcctTttGca aTgttcAagG AAaaTTaTTC tgaAgaagct ggaccaagac

2601 2650

RV_Am_IL ATAGTGATAT TTGGCAGAAG CTCCATGAAC TTCCATTAGC CGTTCGTGAA

RV_Bi_US ATAGTGATAT TTGGCAGAAG CTCCATGAAC TTCCATTAGC CGTTCGTGAA

RV_Am_US ATAGTGATAT TTGGCAGAAG CTCCATGAAC TTCCATTAGC CGTTCGTGAA

RV_Vd_IL ATACAGATAG TAATGAGCTT ATTCCTCCGG TTCCATCACC TATTACGAAA

Consensus ATAgtGATAt TtggcAGaag cTcCaTgaac TTCCATtAgC cgTTcgtgAA

2651 2700

RV_Am_IL GCTCTATTAG CAGATGCAAC ATTGA..... .......... CGACTACTGA

RV_Bi_US GCTCTATTAG CAGATGCAAC ATTGA..... .......... CGACTACTGA

RV_Am_US GCTCTATTAG CAGATGCAAC ATTGA..... .......... CGACTACTGA

RV_Vd_IL ACAACGTCAC CTATTTCTGT TTCTAAGTAA GGATTTAAAA CGTCTATATA

Consensus gCtctaTtAg CagaTgCaac aTtgA..... .......... CGaCTActgA

2701 2750

RV_Am_IL TTCTTTGTAT AATAGAGTCA TTAAGATACA GGAGGAAACA AAAAATAAGG

RV_Bi_US TTCTTTGTAT AATAGAGTCA TTAAGATACA GGAGGAAACA AAAAATAAGG

RV_Am_US TTCTTTGTAT AATAGAGTCA TTAAGATACA GGAGGAAACA AAAAATAAGG

RV_Vd_IL TTTGTAGATT GCTCCGGTAA TCGGTGTAGG GTAGCGTATT GATATCTTAC

Consensus TTctTtGtaT aaTagaGTcA TtaagaTAca GgAGgaaAca aAaAataagg

2751 2800

RV_Am_IL AAAAAATTAG GAAGGAAGCT GTCGAGATTA TCAAAAATGC TACCTTTGAG

RV_Bi_US AAAAAATTAG GAAGGAAGCT GTCGAGATTA TCAAAAATGC TACCTTTGAG

RV_Am_US AAAAAATTAG GAAGGAAGCT GTCGAGATTA TCAAAAATGC TACCTTTGAG

RV_Vd_IL CATATTTTGT ATTATATAGT GCCTGCTTTT TGTGTTGCAG ACATTTATTA

Consensus aAaAaaTTag gaaggAagcT GtCgagaTTa Tcaaaaatgc taccTTtgag

2801 2850

RV_Am_IL GACAAATCCA TATTTGTTAA AAACTTATCT TTACTAAAAG ATGAGGCTAG

RV_Bi_US GACAAATCCA TATTTGTTAA AAACTTATCT TTACTAAAAG ATGAGGCTAG

RV_Am_US GACAAATCCA TATTTGTTAA AAACTTATCT TTACTAAAAG ATGAGGCTAG

RV_Vd_IL GAAGGAACTT TAAATTTTAG CGATATATTG GTGTTTGGAA TCTGTGGTAA

Consensus GAcaaAtCca TAttTgTTAa aaActTATct tTacTaaaAg atgagGcTAg

2851 2900

RV_Am_IL ACATAAGATC TACAGCGCCA TAGTTGAAAA AGGAACGGTG ACTCCTTCG.

RV_Bi_US ACATAAGATC TATAGCGCCA TAGTCGAAAA AGGAACGGTG ACTCCTTCG.

RV_Am_US ACATAAGATC TATAGCGCCA TAGTCGAAAA AGGAACGGTG ACTCCTTCG.

RV_Vd_IL CCTGGGCAAC ATTCTTATCA AAGAGTCCAG AGAAACATTA AGCATTTTAA

Consensus aCataagAtC tatagcgcCA tAGtcgaaAa AGgAACggTg ActccTTcg.

2901 2950

RV_Am_IL ..TTTATTGC CGCCGTCAGA TCCTTTAAAA AACAACAAGG AAGCCAGAAG

RV_Bi_US ..TTTATAGC CGCCGTTAGA TCTTTTAAAA AACAACAAGG AAGCCAGAAG

RV_Am_US ..TTTATAGC CGCCGTTAGA TCTTTTAAAA AACAACAAGG AAGCCAGAAG

RV_Vd_IL GATAAATACC GTCTGTAAGC TCTAATAAGG AAGAGCATTC ATGCGAGTTC

Consensus ..TttATagC cgCcGTtAGa TCtttTAAaa AAcAaCAagg AaGCcAGaag

2951 3000

RV_Am_IL TAATAAGAGA TTCAATTTTG ACATCCTCAC ACACATAACC AAAATCAGAA

RV_Bi_US TAATAAGAGA TTCAATTTTA ACATCTTCAC ACACATAACC AAAATCAGAA

RV_Am_US TAATAAGAGA TTCAATTTTG ACATCTTCAC ACACATAACC AAAATCAGAA

RV_Vd_IL AAAAAATCCA AAAGATACAC AATTTTTTCT CTAGGTAAGT ATCCTTGTAG

Consensus tAAtAAgagA ttcaATtttg AcaTctTcac acAcaTAAcc AaaaTcagAa

3001 3050

RV_Am_IL CCGAATCACG TTCAACTCGA AAATCCGAAG CTCCTCATTA TTCAAAAAGA

RV_Bi_US CCGAATCACG TTCAACTCGA AAATCCGAAG CTCCTCATTA TTAAAAAAGA

RV_Am_US CCGAATCACG TTCAACTCGA AAATCCGAAG CTCCTCATTA TTAAAAAAGA

RV_Vd_IL TGTTAATTGA TTTACCAGGT GTAAAGATAA CGCATCAAAG GCAGAGATGT

Consensus ccgaAtcacg TTcAaCtcGa aaAtccgaAg CtCcTCAtta ttaaAaAaGa

3051 3100

RV_Am_IL ATTCTTAGCT GTTTGCGTTT TACTAAGCCA ACCATTAAAG AAGCACGCCC

RV_Bi_US ATTCTTAGCT GTTTGCGTTT TACTAAGCCA ACCATTAAAG AAGCACGCCC

RV_Am_US ATTCTTAGCT GTTTGCGTTT TACTAAGCCA ACCATTAAAG AAGCACGCCC

RV_Vd_IL ATTTGTTTAT CATAATAAGG GATGGCGCTA ACCAGGGAAG TTTAGAAGTT

Consensus ATTctTagcT gtTtgcgttt tActaaGCcA ACCAttaAAG aagcacgccc

3101 3150

RV_Am_IL AACCTTACTG TTGATTATAA ACATTTCATC AGCTCCGATA ACACCGAGAT

RV_Bi_US AACCTTACTG TTGATTATAA ACATTTCATC AGCTCCGATA ACACCGAAAT

RV_Am_US AACCTTACTG TTGATTATAA ACATTTCATC AGCTCCGATA ACACCGAAAT

RV_Vd_IL AAGGCTAGCT TTGATAGGAT A......... .......... ..ACACAACA

Consensus AAcctTActg TTGATtatAa Acatttcatc agctccgata acACcgAaat

3151 3200

RV_Am_IL CATCAAACAT GCTGAAGAGA CCAAACCAGT TTTTATCAGA AGCTCCGGCT

RV_Bi_US CATCAAACAT GCTGAAGAGA CCAAACCAGT TTTTATCAGA AGCTCCGGCT

RV_Am_US CATCAAACAT GCTGAAGAGA CCAAACCAGT TTTTATCAGA AGCTCCGGCT

RV_Vd_IL ACTCGATTGT ACAGTTCGTG CATAGTTAAT TCTCTTCGGA A...CAGTTT

Consensus caTCaAacaT gCtGaagaga CcaAaccAgT TtTtaTCaGA AgctCcGgcT

3201 3250

RV_Am_IL CAAAAGGCGC CCCGAATTCA ATGGTCGATT ACCAAAAGGG ATATTGAGTA

RV_Bi_US CAAAAGGCGC CCCGAATTCT ATGGTCGATT ACCAAAAGGG ATATTGAGTA

RV_Am_US CAAAAGGCGC CCCGAATTCT ATGGTCGATT ACCAAAAGGG ATATTGAGTA

RV_Vd_IL GAGAAGGGGC TCTTATCTTG TATTTCGACT TCAACAATCT CTGTTTTGAA

Consensus cAaAAGGcGC cCcgAatTct atggTCGAtT aCcAaAAggg aTaTTgaGtA

3251 3300

RV_Am_IL TTACGAAGGG CGAAGCGC.. ....ACTCGA CCCACAGGGC ACTAGAAATA

RV_Bi_US TTACGAAGGG CGAAGCGC.. ....ACTCGA CCCACAAGGC ACTAGAAATA

RV_Am_US TTACGAAGGG CGAAGCGC.. ....ACTCGA CCCACAAGGC ACTAGAAATA

RV_Vd_IL CTATTATCTA TGAAGATATT AGATAGTATA GCAAAATTGG ATAAATCACA

Consensus tTAcgAaggg cGAAGcgc.. ....AcTcgA cCcAcAagGc ActAgaaAtA

3301 3350

RV_Am_IL TGGTTCAGGA ATTGG..... .......... ..TAACATCC AAACCGAAGA

RV_Bi_US TGGTTCAGGA ATTGG..... .......... ..TAACATCC AAACCGAAGA

RV_Am_US TGGTTCAGGA ATTGG..... .......... ..TAACATCC AAACCGAAGA

RV_Vd_IL TGCTTCTAGA ATTTCAGCTG TTTTCTGATA AATAACATTT GAAGGGATCT

Consensus TGgTTCagGA ATTgg..... .......... ..TAACATcc aAAccGAaga

3351 3400

RV_Am_IL TTCCTTTAAA ATGGGAACTA TCAGTGTCTA TGTATTGTCC ACCCTCATAT

RV_Bi_US TTCCTTTAAA ATGGGAACTA TCAGTGTCTA TGTATTGTCC ACCCTCATAT

RV_Am_US TTCCTTTAAA ATGGGAACTA TCAGTGTCTA TGTATTGTCC ACCCTCATAT

RV_Vd_IL TAGATAGATA ATGATAATCT TTTGTCACAG TAATAATTGC CTTAACCAAC

Consensus TtccTttAaA ATGggAActa TcaGTgtCta TgtattgTcC accctCatAt

3401 3450

RV_Am_IL GATGCAGCAA AGAGAGCACT AGAT...... ...GGTGCCA TTGACGACTT

RV_Bi_US GAAGCAGCAA AGAGAGCACT AGAT...... ...GGTGCCA TTGACGACTT

RV_Am_US GAAGCAGCAA AGAGAGCACT AGAT...... ...GGTGCCA TTGACGACTT

RV_Vd_IL GCATAAACGA CTATACCTTC TAATATACAC ACCGGGCTCA AGTATGCAAG

Consensus GaagcAgCaA agAgAgCact agAT...... ...GGtgcCA ttgAcGactt

3451 3500

RV_Am_IL CGATAAGCAG GTGTGTCAT. ..TTGGCACC CATATTTCCC CCAAAGTACA

RV_Bi_US CGATAAGCAG GTATGTCAT. ..TTGGCACC CATATTTCCC CCAAAGTACA

RV_Am_US CGATAAGCAG GTATGTCAT. ..TTGGCACC CATATTTCCC CCAAAGTACA

RV_Vd_IL CCAGACCCAC GGAATTAAAG CATTATTATC GTTAACATCG CCTTGAGCCA

Consensus CgAtAagCAg GtatgTcAt. ..TTggcAcC caTAtttcCc CCaaagtaCA

3501 3550

RV_Am_IL TAAGAAGGTC ACTCATGCAA TTCGGGCCAA GTAAAACAGG TACCTATTAC

RV_Bi_US TAAGAAGGTC ACTTATGCAA TTTGGGCCAA GTAAAACAGG TACCTATTAC

RV_Am_US TAAGAAGGTC ACTTATGCAA TTTGGGCCAA GTAAAACAGG TACCTATTAC

RV_Vd_IL AATCACGATC TCGTAAGATT ATTGACCCAA GTGCAACAGA AGCTAAAGAT

Consensus tAagAaGgTC aCttAtGcaa tTtGggCCAA GTaaAACAGg taCctAttAc

3551 3600

RV_Am_IL TTTAAAAAGA TAGTCCAGGT TTACACCGAT ATTGCAGTGC CATTACATTT

RV_Bi_US TTTAAAAAGA TAGTCCAGGT TTACACCGAT ATTGCAGCTC CATTACATTT

RV_Am_US TTTAAAAAGA TAGTCCAGGT TTACACCGAT ATTGCAGTTC CATTACATTT

RV_Vd_IL CTTCTCTCTG TCACAGTAAG CGAGGAGCCT AGACATATTT CATGATCGAT

Consensus tTTaaaaaga Tagtccaggt ttAcaccgaT Attgcagttc CATtAcattT

3601 3650

RV_Am_IL TACCTTGCAA AATTCCGTTA CCGGCATAGC TGATGCTCGT CTTAAACTGA

RV_Bi_US TACCTTGCAA AATTCCGTTA CCGGCATAGC CGATGCTCGT CTTAAACTGA

RV_Am_US TACCTTGCAA AATTCCGTTA CCGGCATAGC CGATGCTCGT CTTAAACTGA

RV_Vd_IL TACACTTCTT ACTTTTTCGA TCCTAGTGGC TTCTAAACTT .TGAAATAAA

Consensus TACctTgCaa AaTTccgttA cCggcaTaGC cgaTgctCgT cTtAAActgA

3651 3700

RV_Am_IL TCCCCATGTC CCACTTAGGC ATTCAAGGTG AACCCATGCC ATAAGGCCCC

RV_Bi_US TCCCCATGTC CCACTTAGGC ATTCAAGGTG AACCCATGCC ATAAGGCCCT

RV_Am_US TCCCCATGTC CCACTTAGGC ATTCAAGGTG AACCCATGCC ATAAGGCCCT

RV_Vd_IL TAATAATTGC TGGGGCATGA TTTAAATCTG ATTAAGTGCC ATTTGTCATC

Consensus TccccATgtC ccacttAgGc aTTcAAggTG AacccaTGCC ATaaGgCccc

3701 3750

RV_Am_IL AAAAACATTG ACCCCGAAAA CAATAGACCC ACTCATCCAT CCATATCCTT

RV_Bi_US AAGAACATTG ACCCTGAAAA CAATAGACCT ACTCATCCAT CCATATCCTT

RV_Am_US AAGAACATTG ACCCTGAAAA CAATAGACCT ACTCATCCAT CCATATCCTT

RV_Vd_IL A......... ...CCCTCAA TAAAATCATT GCTAATATAT TCGATACATT

Consensus Aagaacattg accCcgaaAA cAAtAgacct aCTcATccAT cCatatCcTT

3751 3800

RV_Am_IL CTGACCGGTT TTAACCAAAA GGAGCCTTTA AGAACAAATC ATCCAATCCA

RV_Bi_US CTGACCGGTT TTAGCCAAAA GGAGCCTTTA AGAACAAATC ATCCAATCCA

RV_Am_US CTGACCGGTT TTAGCCAAAA GGAGCCTTTA AGAACAAATC ATCCAATCCA

RV_Vd_IL CCCGACAATT TATATGAAAA TGAAAATCCA GTATCTTCTT CTTCTCTCCA

Consensus CtgacCggTT TtaaccAAAA gGAgccTttA agAaCaaaTc aTcCaaTCCA

3801 3850

RV_Am_IL AGCAAAACCT CA..CTCTAG ACCAAAAAAC CCAATTCTTC TCCTCAACCA

RV_Bi_US AGCAAAACCT CA..CTCTAG ACCAAAAAAC CCAATTCTTC TCCTCAACCA

RV_Am_US AGCAAAACCT CA..CTCTAG ACCAAAAAAC CCAATTCTTC TCCTCAACCA

RV_Vd_IL GTTAACGCAT AATGCGCAAT TTCATACAAT ACTTTAACTT GGGCTGACAA

Consensus agcAAaaCcT cA..CtCtAg acCAaAaAAc cCaaTtctTc tcctcaACcA

3851 3900

RV_Am_IL CCTATGCCAT TGTAGTAATC TTCCCTTCTT TCCGCAATAG AGACCAGCAA

RV_Bi_US CCTATGCCAT TGTAGTAATC TTCCCTTCTT TCCGCAATAG AGACCAGCAA

RV_Am_US CCTATGCCAT TGTAGTAATC TTCCCTTCTT TCCGCAATAG AGACCAGCAA

RV_Vd_IL CATCAACGTT TGAAAATGTA TTGTCGTGTT TTGTGAGCC. ...CTTTGAA

Consensus CcTatgCcaT TGtAgtaaTc TTccCtTcTT TccgcAatag agaCcagcAA

3901 3950

RV_Am_IL TAATCTGATA ATGCTTTTCT CTATGATTTC CTTAATATCA CAAAAAACCT

RV_Bi_US TAATCTGATA ATGCTTTTCT CTCTGATTTC CTTAATATCA CGAAAAACCT

RV_Am_US TAATCTGATA ATGCTTTTCT CTCTGATTTC CTTAATATCA CGAAAAACCT

RV_Vd_IL AATTCAGACA AT....TGGT CTGTATTTAC TGACATATAG GTACAAGGTT

Consensus tAaTCtGAtA ATgcttTtcT CTcTgaTTtC cttaATATca cgAaAAaccT

3951 4000

RV_Am_IL CTATGTAACG GAGAAAATTA CAAACTTCTA ACAGCAATAA TTTTTTAGAT

RV_Bi_US CTATGTAACG GAGAAAATTA CAAACTTCTA ACAGCAATAA NTTTTTAGAT

RV_Am_US CTATGTAACG GAGAAAATTA CAAACTTCTA ACAGCAATAA TTTTTTAGAT

RV_Vd_IL GCTGATTGAG GTTATATCTA CCAATTCGTA TATTTCTTCC ACCATTATAT

Consensus ctatgTaacG GagAaAatTA CaAAcTtcTA acagcaaTaa ttttTTAgAT

4001 4050

RV_Am_IL CTCATTTTTC AAAAATTTGT ATCAAATATA AACATTCGAT AGATCTCTTA

RV_Bi_US CTCATTTTTC AAAAATTTGT GTCAAATACA AACATTCGAT AGGTCTCTTA

RV_Am_US CTCATTTTTC AAAAATTTGT GTCAAATACA AACATTCGAT AGGTCTCTTA

RV_Vd_IL CTATGAAAAG GACTTCCTGA GTGATTCTCT AGCACTGTGG TGAAATTTTC

Consensus CTcatttttc aAaaattTGt gTcAaataca AaCAtTcgat aGatcTcTTa

4051 4100

RV_Am_IL CTCAAAATAA AATAACAATG TCTATTAAAG AAATTGATTA ATAAACACTA

RV_Bi_US CTCAAAATAA AATAACAATG TCTATTAAAG AAATTGATTA ATAAACACTA

RV_Am_US CTCAAAATAA AATAACAATG TCTATTAAAG AAATTGATTA ATAAACACTA

RV_Vd_IL TAATGGAATA TCTGTCACTG ATTGAGCTAG CCGTTGTAAG GCGTGCCCCA

Consensus ctcaaaAtaA aaTaaCAaTG tcTattaaAG aaaTTGatta ataaaCaCtA

4101 4150

RV_Am_IL ATTATTAAAA AAGAATGTTT AG..AACCGA TAGTGGTAAA AAACCATTGC

RV_Bi_US ATTATTAAAA AAGAATGTTT AG..AACCGA TAGTGGTAAA AAACCATTGC

RV_Am_US ATTATTAAAA AAGAATGTTT AG..AACCGA TAGTGGTAAA AAACCATTGC

RV_Vd_IL AGGGTGAAGT GGCATTAGTG AACCAACCAA TGGCTCTAGA AAGTTTGATT

Consensus AttaTtAAaa aagAaTgtTt Ag..AACCgA TaGtggTAaA AAaccattgc

4151 4200

RV_Am_IL TCACGAAAGT ATAGAAAATC TGAGAGAGCT GAAAAACTGC ATTAACTTTT

RV_Bi_US TCACGAAAGT ATAGAAAATT TGAGAGAGCT GAAAAACTGC ATTAACTTTT

RV_Am_US TCACGAAAGT ATAGAAAATT TGAGAGAGCT GAAAAACTGC ATTAACTTTT

RV_Vd_IL GCCTTCTTGG CTACTGATTC AACGATATAA GAGATACTG. ......TTTT

Consensus tCacgaaaGt aTAgaaAaTc tgaGAgAgct GAaAaACTGc attaacTTTT

4201 4250

RV_Am_IL CGGGTCCATT CAATACAACA GTCCAAACCA ACCTCAGATA AAGC...AAG

RV_Bi_US CGGGTCTATT CAATACAACA GTCCAAACCA ACCTCAGATA AAGC...AAG

RV_Am_US CGGGTCTATT CAATACAACA GTCCAAACCA ACCTCAGATA AAGC...AAG

RV_Vd_IL GATAACTATG AGTTTTATCC TTTGTCTCTG AGCCGAGGTA AGGCGAAAAG

Consensus cgggtCtATt caaTacAaCa gTccaaaCca AcCtcAGaTA AaGC...AAG

4251 4300

RV_Am_IL CCTTAACAAT CAACATCTGC GTCATAGTGA TTTGAAACCG TAGATCCAGC

RV_Bi_US CCTTAACAAT CAACAACTGC GTCATAGTGA TTCGAAACCG TAGATCCAGC

RV_Am_US CCTTAACAAT CAACAACTGC GTCATAGTGA TTCGAAACCG TAGATCCAGC

RV_Vd_IL GGTCCACAGA CATCAAAAGA ATTCCACTTT TCATATATAT CAGGACTGAC

Consensus ccTtaACAat CAaCAactGc gTcatAgTga TtcgAaAccg tAGatCcagC

4301 4350

RV_Am_IL AATACAGCTC GGCCCGGTAG GAATTTAAAT CCGAGTTACT CTAAAGATCT

RV_Bi_US AATACAGCTC GGCCCGGTAG GAATTTAAAT CCGAGTTACT CTAAAAATCT

RV_Am_US AATACAGCTC GGCCCGGTAG GAATTTAAAT CCGAGTTACT CTAAAAATCT

RV_Vd_IL TATAACTTGA ATGTAATCAC CACTATTTCT GTCTTTGCAG TGACGACCGT

Consensus aATAcagctc ggcccggtAg gAaTtTaaaT ccgagTtact ctAaaaatcT

4351 4400

RV_Am_IL AGTCAACGAC ACATCAAGGA ACCATTTGTC CAAAATCAGA GTCCCCGAAC

RV_Bi_US AGTCAACGAC ACATCAAGGA ACCATTTGTC CAAAATCAGA GTCCCCGAAC

RV_Am_US AGTCAACGAC ACATCAAGGA ACCATTTGTC CAAAATCAGA GTCCCCGAAC

RV_Vd_IL CTTCTTTCGC AGACAAAACT TCAAAAGGAC TTGGTACAGT TACACCGACT

Consensus agTCaacgaC AcAtcAAgga aCcAtttGtC caaaatCAGa gtCcCCGAac

4401 4450

RV_Am_IL GCATACCACT CCTCCCCGAA CGCATACCAC AGATGGCTCT ACTATGCACT

RV_Bi_US GCATACCACA TTCCATTACC AAAACGCAAC GATGGCTCAT ACTATGCACT

RV_Am_US GCATACCACA TTCCATTACC AAAACGCAAC GATGGCTCAT ACTATGCACT

RV_Vd_IL ATTGGTCGTC CCCAACTAAC TTCTCTAATC TGATGTGCAG CCATTCTACT

Consensus gcatacCaca ccccactaac aaaacgcaaC gaagGctcat aCtaTgcACT

4451 4500

RV_Am_IL GCAAGCTGAC CGAAAGGTCA AAAAC..... .......GAG ACAAGCTGAC

RV_Bi_US GCAAGCCGAC CGAAAGGTCA AAAAC..... .......GAG ACAAGCTGAC

RV_Am_US GCAAGCCGAC CGAAAGGTCA AAAAC..... .......GAG ACAAGCTGAC

RV_Vd_IL GCAATTGAAA TTACTAGTAA AATGATCAAA AGGTTCTGTT AGATGTTGAG

Consensus GCAAgccgAc cgAaagGTcA AAaac..... .......Gag AcAaGcTGAc

4501 4550

RV_Am_IL ACCCTGTCTG TGATCAACAG CAAGTTTTTA GCTGCTTTGG GAT....TAA

RV_Bi_US ACCCTGTCTG TGATCAACAG CAAGTTTTTA GCTGCTTTGG GAT....TGA

RV_Am_US ACCCTGTCTG TGATCAACAG CAAGTTTTTA GCTGCTTTGG GAT....TGA

RV_Vd_IL CCCATAATGC TTGTAAAAAC GAGGTTCTTT CGTATTTTTT AATCCGGTAA

Consensus aCCcTgtctg TgaTcAAcAg cAaGTTtTTa gcTgcTTTgg gAT....TaA

4551 4600

RV_Am_IL CTCTATGTTG TTTACCAGCT TTCACTTATC AACTAGCTGA GGAACTGGAT

RV_Bi_US CTCTATGTAG TTTACCAGCT TTCACTTATC AACTAGCTGA GGAACTGGAT

RV_Am_US CTCTATGTAG TTTACCAGCT TTCACTTATC AACTAGCTGA GGAACTGGAT

RV_Vd_IL CTCTCTGTAC TTAAA..... ...ACTGATT TTGCTGCTCT TGCACGCATT

Consensus CTCTaTGTag TTtAccagct ttcACTtATc aactaGCTga gGaACtggaT

4601 4650

RV_Am_IL CTGAGTCTTT TAGATAACTT ACCGGTTCAA GCCTTAAGAT GT...AGCGG

RV_Bi_US CTGAGTCTTT TAGATAATTT ACCGGTTCAA GCCTTAAGAT GT...AACGG

RV_Am_US CTGAGTCTTT TAGATAATTT ACCGGTTCAA GCCTTAAGAT GT...AACGG

RV_Vd_IL GTTGTTGTTT GAGAACACTT GGCAGTTAAT TCCTTTTGGT ATCCCACCAG

Consensus cTgagTcTTT tAGAtaAcTT acCgGTTcAa gCCTTaaGaT gT...AaCgG

4651 4700

RV_Am_IL AGCATCTCGA TTTTTGCCAA TTCCGAAAAA ACCATCATGT TATCTGGATA

RV_Bi_US AGCATCTCGA TTTTTGCCAA TTCCGAAAAA ACCATCATGT TATCTGGATA

RV_Am_US AGCATCTCGA TTTTTGCCAA TTCCGAAAAA ACCATCATGT TATCTGGATA

RV_Vd_IL TGATGAAGAT AATATGTCAG ACATTAAAAA TGGATACTGA GGTTCACATG

Consensus aGcatctcga ttTtTGcCAa ttccgAAAAA accATcaTGt taTctggATa

4701 4750

RV_Am_IL TCAACAAAAA CACATATAAA ACGATTGGCC AGATCGAACT TGAAATATTG

RV_Bi_US TCAACAAAAA CACATATAAG ACGATTGGCC AGATCGAACT TGAAATATTG

RV_Am_US TCAACAAAAA CACATATAAG ACGATTGGCC AGATCGAACT TGAAATATTG

RV_Vd_IL CCCACAATGA .......... .......... .........T TGACTTAGTT

Consensus tCaACAAaaA cacatataag acgattggcc agatcgaacT TGAaaTAtTg

4751 4800

RV_Am_IL AGACAAATCC CATCTCATCA AAGAGTTTCA GCTTGGAGGT GTGAGATGTT

RV_Bi_US AGACAAATCC CATCTCATCA AAGAGTTTCA GCTTGGAGGT GTGAGATGTT

RV_Am_US AGACAAATCC CATCTCATCA AAGAGTTTCA GCTTGGAGGT GTGAGATGTT

RV_Vd_IL CTATAAGCTC ACTATCTTCA TAACGCAAAC ACTC.....T CTAATAGTTT

Consensus agAcAAatcC caTcTCaTCA aAgaGtttca gCTtggaggT gTgAgAtgTT

4801 4850

RV_Am_IL TGAGATTATT AAAGTGTGTA AAGAGACCTT TTTTGGATTT CAACACCAAT

RV_Bi_US TGAGATTATT AAAGTGTGTA AAGAGACCTT TTTTGGATTT CAACACCAAT

RV_Am_US TGAGATTATT AAAGTGTGTA AAGAGACCTT TTTTGGATTT CAACACCAAT

RV_Vd_IL TGTTTTTAAT AGCCATGGTT CTGACAGCTT TGGTCGATAT ATCCTTAAGT

Consensus TGagaTTAtT AaagtgtGTa aaGAgAcCTT TttTgGATtT caaCaccAaT

4851 4900

RV_Am_IL TCGAGTTTAC CCGCAAGTCA AAAAAGATAG TTCCGTTAAA CTTTTTGGAA

RV_Bi_US TCGAGTTTAC CCGCAAGTCA AAAAAGACAG TTCCGTTAAA CTTTTTGGAA

RV_Am_US TCGAGTTTAC CCGCAAGTCA AAAAAGACAG TTCCGTTAAA CTTTTTGGAA

RV_Vd_IL ACCGATTCGC CACTTGGTGG TGAAA..... ......TAAT GTTAATGGAC

Consensus tCgagTTtaC CcgcaaGTca aaAAAgacag ttccgtTAAa cTTttTGGAa

4901 4950

RV_Am_IL ATTGCAGCTT TAAAAAGTGT TAAGACCATC GAGGATACCT TTTCCTTTGG

RV_Bi_US ATTGCAGCTT TAAAAAGTGT TAAGACCATC GAGGATACCT TTTCCTTTGG

RV_Am_US ATTGCAGCTT TAAAAAGTGT TAAGACCATC GAGGATACCT TTTCCTTTGG

RV_Vd_IL AATGGATCTT GATATAACAT .AATAGGTTT GACGTATTGT GATAATAAAG

Consensus AtTGcAgCTT tAaAaAgtgT tAAgAccaTc GAgGataccT ttTccTttgG

4951 5000

RV_Am_IL GACCAAAAGA GATTTTGAGT GCCACTGGAT GGAAGAGAAT GAAGTAACAT

RV_Bi_US GACCAAAAGA GATTTTGAAT GCCACTGGAT GGAAGAGAAT GAAGTAACAT

RV_Am_US GACCAAAAGA GATTTTGAAT GCCACTGGAT GGAAGAGAAT GAAGTAACAT

RV_Vd_IL GACAAATAGC TCGTCTTATA ATTGCCGACT GCCAACT..T GATGCATAAG

Consensus GACcAAaAGa gatTtTgAat gccaCtGgaT GgaAgagaaT GAaGtAacAt

5001 5050

RV_Am_IL ATAGCAAAAG AGTCTGCTAT AGAAATGATC ATTTA..... ..........

RV_Bi_US ATAGCAAAAG AGTCTGCTAT AGAAATGATC ATTTA..... ..........

RV_Am_US ATAGCAAAAG AGTCTGCTAT AGAAATGATC ATTTA..... ..........

RV_Vd_IL GTAACATTCG CTTCAACTGG TGTAATCCTG ATGACAGTGG ATCTGGTATA

Consensus aTAgCAaaaG agTCtgCTat aGaAATgaTc ATtta..... ..........

5051 5100

RV_Am_IL ...TTAAATG TCACCGGATC TAATGTTCAC ATTGAAATCC CG........

RV_Bi_US ...TTAAATG TCACCGGATC TAATGTTCAC ATTGAAATCC CG........

RV_Am_US ...TTAAATG TCACCGGATC TAATGTTCAC ATTGAAATCC CG........

RV_Vd_IL CCTTTAATCG TTAACTCATC TAAAAGAGAA ATAGGATACC CGCCAAGTAT

Consensus ...TTAAatG TcAcCggATC TAAtgttcAc ATtGaAatCC CG........

5101 5150

RV_Am_IL .......... .....AATAC AAAAGGTATC CCCTTGCATC TTGGTTACTT

RV_Bi_US .......... .....AATAC AAAAGGTATC CCCTTGCATC TTGGTTACTT

RV_Am_US .......... .....AATAC AAAAGGTATC CCCTTGCATC TTGGTTACTT

RV_Vd_IL TTTGGGCCTG ACTAGAATTC CAAAAATTAC TTTCTCCAAG TTCTGAATTA

Consensus .......... .....AATaC aAAAggTatC ccctTgCAtc TTggttAcTt

5151 5200

RV_Am_IL AATGACAGCC GACGGGGTTG AATATGTGTG GAATGCAAAC ATTAATATCT

RV_Bi_US AATGACAGCC GACGGNGTTG AATATGTGTG GAATGCAAAC ATTAATATCT

RV_Am_US AATGACAGCC GACGGGGTTG AATATGTGTG GAATGCAAAC ATTAATATCT

RV_Vd_IL ATAGAGATCT AACAAGCGTG TTT....... ....GGAATC CTTATTTTCT

Consensus AatGAcAgCc gACggggtTG aaTatgtgtg gaatGcAAaC aTTAaTaTCT

5201 5250

RV_Am_IL CTTGCCCTTT AGAGCATTTC CAAACTGAGT TATGTACAGT ....TCGAGC

RV_Bi_US CTTGCCCTTT AGAGCATTTC CAAACTGAGT TATGTACAGT ....TCGAGC

RV_Am_US CTTGCCCTTT AGAGCATTTC CAAACTGAGT TATGTACAGT ....TCGAGC

RV_Vd_IL TTCTCCTATG ATTCGTTCCA AAGTCTCCGT TGTCTAAACA GGCCTGAAGA

Consensus cTtgCCctTt AgagcaTttc cAaaCTgaGT TaTgTAcAgt ....TcgAGc

5251 5300

RV_Am_IL TGACTTATCA CCAAATGTTC C...ATCCCG TGATTTGTCC AATCTCATTA

RV_Bi_US TGACTTATCA CCAAATGTTC C...ATCCCG TGATTTGTCC AATCTCATTA

RV_Am_US TGACTTATCA CCAAATGTTC C...ATCCCG TGATTTGTCC AATCTCATTA

RV_Vd_IL AGACCGATCT CCAAAATGGG CGAAAATTCA AGATGAGTCA AAAGTGCCCA

Consensus tGACttATCa CCAAAtgttc C...AtccCg tGATttGTCc AAtcTcattA

5301 5350

RV_Am_IL TTGTCTGTCC AAACAACCAT CATATTTTCC ATTTGAA... ..........

RV_Bi_US TTGTCTGTCC AAACAACCAT CATATTTTCC ATTTGAA... ..........

RV_Am_US TTGTCTGTCC AAACAACCAT CATATTTTCC ATTTGAA... ..........

RV_Vd_IL ACCCGTTTGA TAAGAACCAA TTGTCAAAGA ATTGAATGGG AAAAAATCAA

Consensus ttgtcTgTcc aAAcAACCAt catattttcc ATTtgAa... ..........

5351 5400

RV_Am_IL ..TCAGGAGT ACCGTCCGG. TTGGCCCTTT GGAAGTCTGT GCCACTAACA

RV_Bi_US ..TCAGGAGT ACCGTCCGG. TTGGCCCTTT GGAAGTCTGT GCCACTAACA

RV_Am_US ..TCAGGAGT ACCGTCCGG. TTGGCCCTTT GGAAGTCTGT GCCACTAACA

RV_Vd_IL ACTCAGCAGC AGCGGCAGTA TTTGCCCAGA CAGCTGCTAT GTCACTCTCA

Consensus ..TCAGgAGt AcCGtCcGg. TTgGCCCttt ggaagtCTgT GcCACTaaCA

5401 5450

RV_Am_IL CTTCACGTAT TTTTTACACC TCAGAAGGCC TGATGCTTAG AATTTTCAGG

RV_Bi_US CTTCACGTAT TTTTTACACC TCAGAAGGCC TGATGCTTAG AATTTTCAGG

RV_Am_US CTTCACGTAT TTTTTACACC TCAGAAGGCC TGATGCTTAG AATTTTCAGG

RV_Vd_IL AGAGAAGGTA ATTTTTCATT TGAGAAAGGA AAAGTTCTCG ACACCCTTTT

Consensus cttcAcGtat tTTTTaCAcc TcAGAAgGcc tgAtgctTaG Aattttcagg

5451 5500

RV_Am_IL AAAAACATGA CTGAAAAGGC GT........ ......TTTG GGATCTTTTG

RV_Bi_US AAAAACATGA CTGAAAANGC GT........ ......TTTG GGATCTTTTG

RV_Am_US AAAAACATGA CTGAAAAGGC GT........ ......TTTG GGATCTTTTG

RV_Vd_IL AAGAAACATA GTTAAAAGTA CACCCATTCT CAATAATTTC TTACCATAAG

Consensus AAaAAcatgA cTgAAAAggc gt........ ......TTTg ggAtCtTttG

5501 5550

RV_Am_IL CCGGTTACTG AGACCATGCC CAGTCTT... .......... ..GCATTGAC

RV_Bi_US CCGGTCACTG AGACCATGCC AAGTCTT... .......... ..GCATTGAC

RV_Am_US CCGGTCACTG AGACCATGCC AAGTCTT... .......... ..GCATTGAC

RV_Vd_IL CAAATAAAAT TGAAGATGTC CAGGTCTCTG CGGTTTTTAA GGGTAATCCA

Consensus CcggTcActg aGAccATGcC aAGtctT... .......... ..GcAtTgac

5551 5600

RV_Am_IL GTTAGCGTCC AGTATGTTAG AATTTTTGCA AGATAATTTA CAAATTAGTA

RV_Bi_US GTTAGCGTCC AGTATGTTAG AATTTTTGCA AGATAATTTA CAAATTAGTA

RV_Am_US GTTAGCGTCC AGTATGTTAG AATTTTTGCA AGATAATTTA CAAATTAGTA

RV_Vd_IL ATTTTAGTAA AAAAAGTCGA TAGATTGGCA CGAATCATCC TAACCTCATT

Consensus gTTagcGTcc AgtAtGTtag aAttTTtGCA aGAtaatTta cAAatTagTa

5601 5650

RV_Am_IL TAGAGCGAAT ATGGGACAGT ATCCAAAATC AGATATGCAC TATTAAAGAG

RV_Bi_US TAGAGCGAAT ATGGGACAGT ATCCAAAATC AGATTTGCAC TATTAAAGAG

RV_Am_US TAGAGCGAAT ATGGGACAGT ATCCAAAATC ANATTTGCAC TATTAAAGAG

RV_Vd_IL TCGAACAAAT GTCGCTCTTT CAGCATCATT GAGATTTGAA CCTGGAGGAT

Consensus TaGAgCgAAT aTgGgaCagT atcCAaaATc agattTgcAc taTtaAaGAg

5651 5700

RV_Am_IL AATGAGTGGT .....TTTTA GCTAGGTCAT TATTAGGTGT TACCCCTACT

RV_Bi_US AATGAGTGGT .....TTTTA GCTAGATCAT TATTAGGTGT TACCCCTACT

RV_Am_US AATGAGTGGT .....TTTTA GCTAGATCAT TATTAGGTGT TACCCCTACT

RV_Vd_IL CATGAATTGC CAGAATAATA ACTTGATTAT CACCTTGTCC CGACAGTTGA

Consensus aATGAgTgGt .....TttTA gCTaGaTcAT tAttagGTgt tacCccTact

5701 5750

RV_Am_IL GAATCAGCTA AAATATTCTT TAATGAAAGA GAAATAACCG CCAGCTATCT

RV_Bi_US GAATCAGCTA AAATATTCTT TAATGAAAGA GAAATAACCG CCAGCTATCT

RV_Am_US GAATCAGCTA AAATATTCTT TAATGAAAGA GAAATAACCG CCAGCTATCT

RV_Vd_IL AAACTATGAG GTGTTGTAGA AACAACGGTA TAAATTGCTG CTACTGTGAC

Consensus gAAtcAgcta aaaTatTctt tAatgaaagA gAAATaaCcG CcAgctatct

5751 5800

RV_Am_IL GCGTGGTGGA TTGATTGTTC GAAATTGTAT CAAAGTTCGG GTTTACCTTG

RV_Bi_US GCGTGGTGGA TTGATTGTTC GAAATTGTAT CAAAGTTCGG GTTTACCTTG

RV_Am_US GCGTGGTGGA TTGATTGTTC GAAATTGTAT CAAAGTTCGG GTTTACCTTG

RV_Vd_IL CAGTGTCCAA CCTTTTTGTC TAAGACCTTC CTGACCACCT TTTGTTCCTT

Consensus gcGTGgtggA ttgaTTgtTC gAAattgTat CaaAgttCgg gTTtacCtTg

5801 5850

RV_Am_IL ACTCCAATCC TACTCGATGC GGGAAATTTT GGCCAATTAA AAATAGCCTT

RV_Bi_US ACTCCAATCC TACTCGATGC GGGAAATTTT GGCCAATTAA AAATAGCCTT

RV_Am_US ACTCCAATCC TACTCGATGC GGGAAATTTT GGCCAATTAA AAATAGCCTT

RV_Vd_IL TATACGAGCA AATATTGTCT TTTAGATCTC CTCGCATGTT GAATTCTAGT

Consensus acTcCaAtCc tActcgaTgc gggAaATtTt ggCcaATtaa aAATagcctT

5851 5900

RV_Am_IL GACTTTTTAG AGATTGCTAC AAATACCATC AGTAGAGGTA CCACAGATGA

RV_Bi_US GACTTTTTAG AGATTGCTAC AAATACCATC AGTAGAGGTA CCACAGATGA

RV_Am_US GACTTTTTAG AGATTGCTAC AAATACCATC AGTAGAGGTA CCACAGATGA

RV_Vd_IL TCAGTTCCAT CTTCTGCTAA ATAAT..... AGTGAGATTC TTTGAAAATA

Consensus gactTTttAg agatTGCTAc AaAtaccatc AGTagaggTa ccacAgAtgA

5901 5950

RV_Am_IL TCATTGTTCC CTAAGTGAAG GACATTTAAT CTACATCAAC GAGACCCACT

RV_Bi_US TCATTGTTCC CTAAGTGAAG GACATTTAAT CTACATTAAC GAGACCCACT

RV_Am_US TCATTGTTCC CTAAGTGAAG GACATTTAAT CTACATTAAC GAGACCCACT

RV_Vd_IL TCATGAGTTC GTGATATTAG ATGATTAAAG CCAAACAATT GATCCAATCT

Consensus TCATtgtTcC cTaAgtgaAG gacATTtAAt CtAcAttAac GAgaCccaCT

5951 6000

RV_Am_IL ATTTGGGATT AGACAATCAT AAGCTTTATA TTTTAGATCG ACCAGTGCTG

RV_Bi_US ATTTGGGATT AGACAATCAT AAGCTTTATA TTTTAGATCG ACCAGTGCTG

RV_Am_US ATTTGGGATT AGACAATCAT AAGCTTTATA TTTTAGATCG ACCAGTGCTG

RV_Vd_IL TTCAAATATT GGTC.....T TACCAGTCTA TCACGCATAT TGTGGTTCCA

Consensus aTttgggATT aGaCaatcaT aAgCttTaTA TtttagATcg accaGTgCtg

6001 6050

RV_Am_IL TCTTCTATGT CGCACCTTGT AATGCATCCC TTACAATTTG CGCCTTCTGA

RV_Bi_US TCTTCTATGT CGCACCTTGT AATGCATCCC TTACAATTTG CGCCTTCTGA

RV_Am_US TCTTCTATGT CGCACCTTGT AATGCATCCC TTACAATTTG CGCCTTCTGA

RV_Vd_IL TTTTTCAAAA TCCATATTTA TGGTTATATA TGTACATTTC CTCTTACCTG

Consensus TcTTctAtgt cgCAccTTgt aatgcATccc TtacaATTTg CgCcTtCtga

6051 6100

RV_Am_IL CCTATACAGT CTACATGAGC TTCAAGAAGA CGAATTCAGC AGGTATTCAA

RV_Bi_US CCTATACAGT CTACATGAAC TTCAAGAAGA TGAATTCAGC AGGTATTCAA

RV_Am_US CCTATACAGT CTACATGAAC TTCAAGAAGA TGAATTCAGC AGGTATTCAA

RV_Vd_IL GCGATCTAG. ...CTTGATC CCTTGTTAAA TTAATTAAGC GTTTTTTCAG

Consensus cCtATacAGt ctaCaTGAaC ttcaagaAgA tgAATTcAGC aggTaTTCAa

6101 6150

RV_Am_IL TTAGCAAAAT TAACAAAAGA TTAGAGGCTT TGACAACAAC GTTACTGAAT

RV_Bi_US TTAGCAAAAT TAACAAAAGA TTAGAGGCTT TGACAACAAC GTTACTGAAT

RV_Am_US TTAGCAAAAT TAACAAAAGA TTAGAGGCTT TGACAACAAC GTTACTGAAT

RV_Vd_IL TTCCAGATCC TTATATGTTA TAGTTATTTC TTTAAAGTAC GGTAAT.ATT

Consensus TTagcaAaat TaAcAaaagA TtagaggcTt TgacAAcaAC GtTAcTgAaT

6151 6200

RV_Am_IL TATCAAATGG ATGAAATCCC CGGTGTTATA AACCCTAGGG ACCCCTGGAC

RV_Bi_US TATCAAATGG ATGAAATCCC CGGTGTTATA AACCCTAGGG ACCCCTGGAC

RV_Am_US TATCAAATGG ATGAAATCCC CGGTGTTATA AACCCTAGGG ACCCCTGGAC

RV_Vd_IL GATTTAGCTA TCATTGCTTC TGTTAATACA AAATAAAGCC GCACTGGGAA

Consensus tATcaAatgg atgaaatccC cGgTgtTAtA AAccctAGgg aCcCctGGAc

6201 6250

RV_Am_IL ACCTATATCG AAGTTCTTTG TGGGGATTTG GCATAAAATA ACCTCGCCCT

RV_Bi_US ACCGATATCG AAATTCTTTG TGGGGATTTG GCATAAAATA ACCTCGCCCT

RV_Am_US ACCGATATCG AAATTCTTTG TGGGGATTTG GCATAAAATA ACCTCGCCCT

RV_Vd_IL AGTCATTAAA GAAAACATTC TAGGCTCTAG TTTTAA.... .........T

Consensus AccgATatcg aAattCtTTg TgGGgatTtG gcaTAAaata acctcgcccT

6251 6300

RV_Am_IL TACCTTTGTT TATCATCACT CTCGCTATTT TATTAATTGT GGCAAAAGTT

RV_Bi_US TACCTTTGTT TATCATCACT CTCGCTATTT TATTAATTGT GGCAAAAGTT

RV_Am_US TACCTTTGTT TATCATCACT CTCGCTATTT TATTAATTGT GGCAAAAGTT

RV_Vd_IL TCTCGTTCTT TTGACACTTG ACCAATAATC AATTCTTCTT TCGAGAAACC

Consensus TacCtTTgTT TatcatCact ctCgcTAtTt tATTaaTtgT ggcAaAAgtt

6301 6350

RV_Am_IL ATTCATCTGA TTTTTGTTTG GAAGTTTCGT TCCAGATACA CAAGACCAGG

RV_Bi_US ATTCATCTGA TTTTTGTTTG GAAGTTTCGT TCCAGATACA CAAGACCAGG

RV_Am_US ATTCATCTGA TTTTTGTTTG GAAGTTTCGT TCCAGATACA CAAGACCAGG

RV_Vd_IL CTTAAGGTCC TTCTCCTCTA AAAAATCATG GGGCGAGATA TCGGGTGAAT

Consensus aTTcAtcTga TTtTtgTtTg gAAgtTtcgt tccaGAtAcA caaGaccAgg

6351 6400

RV_Am_IL GCAATGGCGG TATTAAAGTT TCCTTTAACC CTATCTCTCC AACCCGAACA

RV_Bi_US GCAATGGAGG TATTAAAGTT TCCTTTAACC CTATCTCTCC AACCTGAACA

RV_Am_US GCAATGGAGG TATTAAAGTT TCCTTTAACC CTATCTCTCC AACCTGAACA

RV_Vd_IL CAAGCCATTT TAATATTAAT CTTTTTTGTT CTGCAAGTCC CACACTCTTA

Consensus gcAatggagg TAtTAaagtT tccTTTaacc CTatctcTCC aACccgaacA

6401 6450

RV_Am_IL AGCAAATACA CACGCTCCGC CAACATAGAA GACAGAGAGC CAAGACAAGT

RV_Bi_US AGCAAACACA CACGCTCCGC CAACATAGAA GACAGAGAGC CAAGACAAGT

RV_Am_US AGCAAACACA CACGCTCCGC CAACATAGAA GACAGAGAGC CAAGACAAGT

RV_Vd_IL TGACTTCTAT AAGTCTCTCG TAACTCTGAC AAAATTGGTG AACATGATTT

Consensus aGcaaacaca cAcgCTCcgc cAACataGAa gAcAgaGagc cAagacAagT

6451 6500

RV_Am_IL A.....CACC AACGCAACCA GCAACTTCTT CACTTTCAAC ACAAAAGGAA

RV_Bi_US A.....CACC AACGCAACCA GCAACTTCTT CACTTTCAAC ACAAAAGGAA

RV_Am_US A.....CACC AACGCAACCA GCAACTTCTT CACTTTCAAC ACAAAAGGAA

RV_Vd_IL ATCCTCCATC ATTTCTGCTA AAGAGATTTT GTCATTTGAA GAAAAAGTCT

Consensus A.....CAcC AacgCaaCcA gcaActTcTT caCtTTcaAc acAAAAGgaa

6501 6550

RV_Am_IL AATCAACACG GAATGTTCCT GCGAAGCACC ATTCCCCCCC TCCGCACACC

RV_Bi_US AATCAACACG GAATGTTCCT GCGAAGCATC ATTCTCCCCC TCCGCACACC

RV_Am_US AATCAACACG GAATGTTCCT GCGAAGCATC ATTCCCCCCC TCCGCACACC

RV_Vd_IL TTTCAAACCT AA........ .......... .......... TGCGCAACCA

Consensus aaTCAAcaCg gAatgttcct gcgaagcatc attccccccc TcCGCAcaCc

6551 6600

RV_Am_IL CCCAATTTTG GGAAATAAAA TAGCTTTGAT CAATAGATAA TCCCTCACCC

RV_Bi_US CCCAATTTTG GGAAATAAAA TAGCTTTGAT CAATAGATAA TCCCTCACCC

RV_Am_US CCCAATTTTG GGAAATAAAA TAGCTTTGAT CAATAGATAA TCCCTCACCC

RV_Vd_IL AGCACTGTTA GTGTTTTCTA TGTGATGTTC ATTTGGATA. ....TATTCC

Consensus ccCAaTtTTg GgaaaTaaaA TagctTtgat caaTaGATAa tcccTcacCC

6601 6650

RV_Am_IL GAAATCGGAA TACCGCACAG CACCCTCGAT CCCAATTCCC TGCAAGCTCC

RV_Bi_US GAAATCGGAA TACCGCACAA CACCCTCGAT CCCAATTCCC TGCAAGCTCC

RV_Am_US GAAATCGGAA TACCGCACAA CACCCTCGAT CCCAATTCCC TGCAAGCTCC

RV_Vd_IL AAGATCTAAA CAGTTTTTAA AGTAGTGATT ATCAGGTAGA TCTAAAGAAC

Consensus gAaATCggAA tAccgcacAa cacccTcgaT ccCAatTccc TgcAAgctcC

6651 6700

RV_Am_IL AAGCGATCTA GTTTAAGCCA AACCACAATG AAACCCTTCA AGCGACAAAG

RV_Bi_US AAGCGATCCA GTTTAAGCCA AACCACAATA AAACCCTTCA AGCGACAAAG

RV_Am_US AAGCGATCCA GTTTAAGCCA AACCACAATA AAACCCTTCA AGCGACAAAG

RV_Vd_IL AAGGAGGCCA GTG....... .........A TTATGTTTCT TAATATAATT

Consensus AAGcgatCcA GTttaagcca aaccacaata aaAcccTTCa agcgAcAAag

6701 6750

RV_Am_IL ATAAAGGACC GTCAAACATA GACAAAAGTT CACCAGACCC GTCACAAAAC

RV_Bi_US ATAAAGGACC GTCAAACATA GACAAAAGTT CACCAGACCC GTCACAAAAC

RV_Am_US ATAAAGGACC GTCAAACATA GACAAAAGTT CACCAGACCC GTCACAAAAC

RV_Vd_IL TTTAAGAAAC ATTTTAACCC AATTACTGGT TACCAAATCG AC........

Consensus aTaAAGgAcC gTcaaAcata gAcaAaaGtT cACCAgAcCc gtcacaaaac

6751 6800

RV_Am_IL CAAAGATAAT GACACTCCGT ATCCAGCCAT CCAGTTAGAA CTTTATCTAC

RV_Bi_US CAAAGATAAC GACACTCCGT ATCCAGCCAT CCAGTTAGAA CTTTATCTAC

RV_Am_US CAAAGATAAC GACACTCCGT ATCCAGCCAT CCAGTTAGAA CTTTATCTAC

RV_Vd_IL .......... .......... .........T GCTTTAGGAT CTATGTATTT

Consensus caaagataac gacactccgt atccagccaT cCagTtaGAa CTtTaTcTac

6801 6850

RV_Am_IL AAACAGTTGT AATAATTCTT ATGATCTTTA TGGTCTCATT ATCCCCAGAA

RV_Bi_US AAACAGTTGT AATAATTCTT ATGATCTCTA TGGTCTCATT ATCCCCAGAA

RV_Am_US AAACAGTTGT AATAATTCTT ATGATCTCTA TGGTCTCATT ATCCCCAGAA

RV_Vd_IL AGACTTATTT CCAACTTCTT TTATCTTTTG CGTTCCTTCC CTGCCGTACA

Consensus AaACagtTgT aatAaTTCTT aTgatcTcTa tGgTCtcatt aTcCCcagaA

6851 6900

RV_Am_IL TATGTCTCTC ACAAAGCAAT GCCAGAACCA CACAACTATC ACACCGAAAA

RV_Bi_US TATGTCTCTC ACAAAGCAAT GCCAGAACCA CACAACTATC ACACCAAAAA

RV_Am_US TATGTCTCTC ACAAAGCAAT GCCAGAACCA CACAACTATC ACACCAAAAA

RV_Vd_IL CGTTTGGATG ACCCCACAAT CTAAAAAGGC CGAATAGTTC AGAT......

Consensus taTgTctcTc ACaaagCAAT gccAgAAcca CacAactaTC AcAccaaaaa

6901 6950

RV_Am_IL AACCTCGGCA CAATCAGGCT CATTAAACAA AAAATCAAGA ATTTCGCAAA

RV_Bi_US AACCTCGGCA CAATCAGGCT CATTAAACAA AAAACCAAGA ATTTCGCAAA

RV_Am_US AACCTCGGCA CAATCAGGCT CATTAAACAA AAAACCAAGA ATTTCGCAAA

RV_Vd_IL AGCTTATGTT TGTTAGATAG TCCTAAAAAG AAATTATACA ATTCCCGAGC

Consensus AaCcTcgGca caaTcaggct catTAAAcAa AAAaccaAgA ATTtCgcAaa

6951 7000

RV_Am_IL GATCCACCTC CCTCGTCAAA ACTCCAAAAT AAAATTAGCC TGGCAAGTCC

RV_Bi_US GATCCAGCTC CCTCGTCAAA ACTCCAAAAT AAAATTAGCC TGGCAAGTCC

RV_Am_US GATCCAGCTC CCTCGTCAAA ACTCCAAAAT AAAATTAGCC TGGCAAGTCC

RV_Vd_IL AAATACAACT TCTTCTGGAT TGACACTTTT GATATCATCC AGGATATTAT

Consensus gAtccagctc cCTcgTcaAa actCcaaaaT aAaATtAgCC tGGcaAgTcc

7001 7050

RV_Am_IL ACAAAACACC GTCACAAACA CATCCCAGTA TATCACCAAT ATCTGCCCCC

RV_Bi_US ACAAAACACC GTCACAAACA CATCCCAGTA TATCACCAAT ATCTGCCCCC

RV_Am_US ACAAAACACC GTCACAAACA CATCCCAGTA TATCACCAAT ATCTGCCCCC

RV_Vd_IL CTAAAAAAGC ATTTTCACAA TTTGGGTCAT CAGCTCCTTC ATCTAGAGTC

Consensus acAAAAcAcC gTcacaAacA caTcccagta tAtCaCCaat ATCTgccccC

7051 7100

RV_Am_IL ACACACGCCC CTCCCCAAAA AAGACACACC AGATGTGATA AGCAAATCCC

RV_Bi_US ACACACGCCC CTCCCCAAAA AAGACACACC AGATGTGATA AGCAAATCCC

RV_Am_US ACACACGCCC CTCCCCAAAA AAGACACACC AGATGTGATA AGCAAATCCC

RV_Vd_IL ATTAGCGCAG CGTAAATAAG GGCTTCGAAC TGAGCTATTA TTTTGTAACC

Consensus AcacaCGCcc CtccccaAAa aagacacAcC aGAtgTgaTA agcaaatcCC

7101 7150

RV_Am_IL TATCAATTTT CGGCAAACAA GACCCCTTTT ACTTCTTTCA CAACCAAAAG

RV_Bi_US TGTCAATTTT CGGCAAACAA GACCCCTTTT GCTTCTTTCA CAACCAAAAG

RV_Am_US TGTCAATTTT CGGCAAACAA GACCCCTTTT GCTTCTTTCA CAACCAAAAG

RV_Vd_IL TCTCCGTTTG TTGTTCTTAA GAACACGCAT GCCCCAACAT AGAACACGGT

Consensus TgTCaaTTTt cgGcaaacAA GAcCcCtttT gCttCtttca caAcCAaaag

7151 7200

RV_Am_IL CAAAC..AAT CGGAAAACAG .......... .......... .........C

RV_Bi_US CAAAC..AAT CGGAAAACAG .......... .......... .........C

RV_Am_US CAAAC..AAT CGGAAAACAG .......... .......... .........C

RV_Vd_IL CAATCTCAAT CGGAGAAGGG ATAGGACTGT ATTTCCAATT AGTTTGTAAC

Consensus CAAaC..AAT CGGAaAAcaG .......... .......... .........C

7201 7250

RV_Am_IL CCACAAAGCG AACACCCATC AGCATACTCC GGAGTTCAAT AAGGAAATTT

RV_Bi_US CCACAAAGCG AACACCCATC AGCATACTCC GGAGTTCAAT AAGGAAATTT

RV_Am_US CCACAAAGCG AACACCCATC AGCATACTCC GGAGTTCAAT AAGGAAATTT

RV_Vd_IL CTAGATGCAT AATATATCAG ATGATACTGT TGAGACAAAT CAGAAAGCAT

Consensus CcAcAaagcg AAcAcccatc AgcATACTcc gGAGttcAAT aAGgAAattT

7251 7300

RV_Am_IL CTAAAAACAG ACTGTTTCAT ACTGCAACAG T......CCA ATTGAACATT

RV_Bi_US CTAAAA.CAG ACTGTTTCAT ACTGCAACAG T......CCA ATTGAACATT

RV_Am_US CTAAAA.CAG ACTGTTTCAT ACTGCAACAG T......CCA ATTGAACATT

RV_Vd_IL ATAAAAAGTT TCTCGAGTAA GAAAGACCCC TATGTTTCCT ATTGGTAAAA

Consensus cTAAAA.cag aCTgtttcAt actgcAaCag T......CCa ATTGaacAtt

7301 7350

RV_Am_IL ACATTACCAA TTTATTTGAG AGGAAAATAT ATAAATTGCA CAATAGACAT

RV_Bi_US ACATTACCAA TTTATTTGAG AGGAAAATAT ATAAATTGCA CAATAGACAT

RV_Am_US ACATTACCAA TTTATTTGAG AGGAAAATAT ATAAATTGCA CAATAGACAT

RV_Vd_IL AGACGCCGTC TTTATGAAAT ACAATAGTAC TACCATT.CG CGAAAGGTGA

Consensus AcAttaCcaa TTTATttgAg AggAaAaTAt ataaATTgCa CaAtAGacat

7351 7400

RV_Am_IL TTAAGAAAAA AACTATGGAA GATATAGAAG AGTGGGATGA ACTCTTCGAC

RV_Bi_US TTAAGAAAAA AACTATGGAA GATATAGAAG AGTGGGATGA ACTCTTCGAC

RV_Am_US TTAAGAAAAA AACTATGGAA GATATAGAAG AGTGGGATGA ACTCTTCGAC

RV_Vd_IL CCAATAAAAA AAGTTTGAGT CCTTAACTAT ATCGTACGGA AGGTGTGGAA

Consensus ttAAgAAAAA AAcTaTGgaa gaTatAgaAg AgtGggatGA ActctTcGAc

7401 7450

RV_Am_IL GAGTCAGAAG AAAGTCAGGA GTTAAAGA.. .......... .....TATAT

RV_Bi_US GAGTCAGAAG AAAGTCAGGA GTTAAAGA.. .......... .....TATAT

RV_Am_US GAGTCAGAAG AAAGTCAGGA GTTAAAGA.. .......... .....TATAT

RV_Vd_IL AATGATAACA GGAGGCAGAA ATTATAGAAC CTATAATTGT CATAATTTTT

Consensus gAgtcagAag aaAGtCAGgA gTTAaAGA.. .......... .....TaTaT

7451 7500

RV_Am_IL CTAGAAAGAA CTCTGTCGTC TCCTAT.... .......... .......AAG

RV_Bi_US CTAGAAAGAA CTCTGTCGTC TCCTAT.... .......... .......AAG

RV_Am_US CTAGAAAGAA CTCTGTCGTC TCCTAT.... .......... .......AAG

RV_Vd_IL GCCCAAAGAA ACATATACCA TTCTATGGAG TTTATATCAG CACACACATC

Consensus ctagAAAGAA ctcTgTcgtc TcCTAT.... .......... .......Aag

7501 7550

RV_Am_IL ATGGTTTTTA TATTCTTTAT CTCTCTCTGA TACGCTATTT C.........

RV_Bi_US ATGGTTTTTA TATTCTTTAT CTCTCTCTGA TACGCTATTT C.........

RV_Am_US ATGGTTTTTA TATTCTTTAT CTCTCTCTGA TACGCTATTT C.........

RV_Vd_IL ATAGCCTTTT AAGTTTTGAA CCAGCCCTGC TTCACCAGCC CCCTTTAGTT

Consensus ATgGttTTTa tAtTcTTtAt CtctCtCTGa TaCgCtAttt C.........

7551 7600

RV_Am_IL .......AAC GTCATAGGTC GCATCTTTCA GTAAAA..TG CCTCTCTATA

RV_Bi_US .......AAC GTCATAGGCC GCATCTTTCA GTAAAA..TG CCTCTCTATA

RV_Am_US .......AAC GTCATAGGCC GCATCTTTCA GTAAAA..TG CCTCTCTATA

RV_Vd_IL GCAGTATAAG ATTAGTATTT GCGACCTTCA CTAAAAGTTT ATGCTCCATT

Consensus .......AAc gTcAtaggcc GCatCtTTCA gTAAAA..Tg cctCTCtATa

7601 7650

RV_Am_IL ATCCGTAATT TTAAACTCTT AATGTCGAAA GGAAAGGAAC TCAATATCTT

RV_Bi_US ATCCGTAACT TTAAACTCTT AATGTCGAAA GGAAAGGAAC TCAATATCTT

RV_Am_US ATCCGTAACT TTAAACTCTT AATGTCGAAA GGAAAGGAAC TCAATATCTT

RV_Vd_IL CTCCATGATT TCCAAAGGTG TGGTTTGATA ATAGGGGGGG T........T

Consensus aTCCgTaAcT TtaAActcTt aatgTcGAaA ggAaaGGaac TcaatatctT

7651 7700

RV_Am_IL TTTGATGAAA ATCATTGATA GACCTGACGA AGTTGTACCA TTTTTCTACA

RV_Bi_US TTTGACGAAA ATCATTGATA GACCTGACGA AGTTGTACCA TTTTTCTACA

RV_Am_US TTTGACGAAA ATCATTGATA GACCTGACGA AGTTGTACCA TTTTTCTACA

RV_Vd_IL TTGTAGAAAA ATGGTACAAC TTCGTCAGGT CTATCAATGA TTTTCATCAA

Consensus TTtgAcgAAA ATcaTtgAta gaCcTgAcGa agtTgtAccA TTTTtcTacA

7701 7750

RV_Am_IL AAACCC.... ....CCCCTA CTATCAAACC ACACCTTTGG AAATCATGGA

RV_Bi_US AAACCC.... ....CCCCTA TTATCAAACC ACACCTTTGG AAATCATGGA

RV_Am_US AAACCC.... ....CCCCTA TTATCAAACC ACACCTTTGG AAATCATGGA

RV_Vd_IL AAAGATATTG AGTTCCTTTC CTTTCGACAT TAAGAGTTTA AAGTTACGGA

Consensus AAAccc.... ....CCccTa cTaTCaAacc acAcctTTgg AAaTcAtGGA

7751 7800

RV_Am_IL GAATGGAGCA TAAACTTTTA GTGAAGGTCG CAAATACTAA TCTTATACTG

RV_Bi_US GAATGGAGCA TAAACTTTTA GTGAAGGTCG CAAATACTAA TCTTATACTG

RV_Am_US GAATGGAGCA TAAACTTTTA GTGAAGGTCG CAAATACTAA TCTTATACTG

RV_Vd_IL TTATAGAGAG GCA..TTTTA CTGAAAGATG CGACCTATGA CGTTGAAATA

Consensus gaATgGAGca taAacTTTTA gTGAAgGtcG CaAatacTaA tcTTatAcTg

7801 7850

RV_Am_IL CAACTAAAGG GGGCTGGTGA AGCAGGGCTG GTTCAAAACT TAAAAGGCTA

RV_Bi_US CAACTAAAGG GGGCTGGTGA AGCAGGGCTG GTTCAAAACT TAAAAGGCTA

RV_Am_US CAACTAAAGG GGGCTGGTGA AGCAGGGCTG GTTCAAAACT TAAAAGGCTA

RV_Vd_IL .......... ......GCGT ATCAGAGAGA GATAAAGAAT ATAAAAACCA

Consensus caactaaagg gggctgGtGa AgCAGgGctg GtTcAAaAcT taAAAggCtA

7851 7900

RV_Am_IL TGATGTGTGT GCTGATATAA ACTCCATAGA ATGGTATATG TTTCTTTGGG

RV_Bi_US TGATGTGTGT GCTGATATAA ACTCCATAGA ATGGTATATG TTTCTTTGGG

RV_Am_US TGATGTGTGT GCTGATATAA ACTCCATAGA ATGGTATATG TTTCTTTGGG

RV_Vd_IL TCTTATAGGA GACGACAGAG .......... .......... .TTCTTTCTA

Consensus TgaTgTgtGt GctGAtAtAa actccataga atggtatatg tTTCTTTggg

7901 7950

RV_Am_IL CAAAAATTAT GACAATTATA GGTTCTATAA TTTCTGCCTC CTGTTATCAT

RV_Bi_US CAAAAATTAT GACAATTATA GGTTCTATAA TTTCTGCCTC CTGTTATCAT

RV_Am_US CAAAAATTAT GACAATTATA GGTTCTATAA TTTCTGCCTC CTGTTATCAT

RV_Vd_IL GATATAT... .......... ....CTTTAA CTCCTGACTT TCTTCTGACT

Consensus cAaAaATtat gacaattata ggttCTaTAA tTtCTGcCTc ctgTtatcaT

7951 8000

RV_Am_IL TTTCCACACC TTCCGTACGA TATAGTTAAG GACTCAAACT TTTTTTATTG

RV_Bi_US TTTCCACACC TTCCGTACGA TATAGTTAAG GATTCAAACT TTTTTTATTG

RV_Am_US TTTCCACACC TTCCGTACGA TATAGTTAAG GATTCAAACT TTTTTTATTG

RV_Vd_IL CGTCGAAGAG TTCATCCCAC TCTTCTATAT CTTCCATAGT TTTTTTCTAA

Consensus ttTCcAcacc TTCcgtaCga TaTagTtaAg gattCAaAcT TTTTTTaTtg

8001 8050

RV_Am_IL GTCACCTTTC GCGAATGGTA GTACTATTGT TTTTCATAAA GACGGCGTCT

RV_Bi_US GTCACCTTTC GCGAATGGCA GTACTATTGT TTTTCATAAA GACGGCGTCT

RV_Am_US GTCACCTTTC GCGAATGGCA GTACTATTGT TTTTCATAAA GACGGCGTCT

RV_Vd_IL AATGTCTATT GTGCAATTTA TATATTTTCC TCTCAAATAA ATTGGTAATG

Consensus gtcacCTtTc GcGaAtggcA gtacTaTTgt TtTtcAtaAA gacGGcgtct

8051 8100

RV_Am_IL TTTTACCAAT AGGAAACATA GGGGTCTTTC TTACTCGAGA AACTTTTTAT

RV_Bi_US TTTTACCAAT AGGAAACATA GGGGTCTTTC TTACTCGAGA AACTTTTTAT

RV_Am_US TTTTACCAAT AGGAAACATA GGGGTCTTTC TTACTCGAGA AACTTTTTAT

RV_Vd_IL TAATGTTCAA TTGGACTGTT GCAGTATGAA ACAGTCTG.. ....TTTTAG

Consensus TttTaccaAt agGaAacaTa GggGTcTttc ttAcTCgaga aactTTTTAt

8101 8150

RV_Am_IL ATGCTTTCTG ATTTGTCTCA ACAGTATCAT CTGATATATT ATGCATCTAG

RV_Bi_US ATGCTTTCTG ATTTGTCTCA ACAGTATCAT CTGATATATT ATGCATCTAG

RV_Am_US ATGCTTTCTG ATTTGTCTCA ACAGTATCAT CTGATATATT ATGCATCTAG

RV_Vd_IL AAATTTCCTT ATTGAACTCC GGAGTATGCT GATGGGTGTT CGCTTTGTGG

Consensus AtgcTTtCTg ATTtgtCTCa acAGTATcaT ctgataTaTT atgcaTcTaG

8151 8200

RV_Am_IL GTTACAAACT AATTGGAAAT ACAGTCCTAT CCCTTCTCCG ATTGAGATTG

RV_Bi_US GTTACAAACT AATTGGAAAT ACAGTCCTAT CCCTTCTCCG ATTGAGATTG

RV_Am_US GTTACAAACT AATTGGAAAT ACAGTCCTAT CCCTTCTCCG ATTGAGATTG

RV_Vd_IL GCTGTTTTCC GATTGT.... .......... .......... .......TTG

Consensus GtTacaaaCt aATTGgaaat acagtcctat cccttctccg attgagaTTG

8201 8250

RV_Am_IL ACCGGGTTCT ATGTTGGGGC ATGCGTGTTC TTAAGAACAA CAAACGGAGA

RV_Bi_US ACCGTGTTCT ATGTTGGGGC ATGCGTGTTC TTAAGAACAA CAAACGGAGA

RV_Am_US ACCGTGTTCT ATGTTGGGGC ATGCGTGTTC TTAAGAACAA CAAACGGAGA

RV_Vd_IL CTTTTGGTTG TGAAAGAAGT AAAAGGGGTC TTGTTTGCCG AAAATTGATA

Consensus accgtGtTct atgttGggGc AtgcGtGtTC TTaagaaCaa cAAAcgGAgA

8251 8300

RV_Am_IL GGTTACAAAA TAATAGCTCA GTTCGAAGCC CTTATTTACG CTGCGCTAAT

RV_Bi_US GGTTACAAAA TAATAGCTCA GTTCGAAGCC CTTATTTACG CTGCGCTAAT

RV_Am_US GGTTACAAAA TAATAGCTCA GTTCGAAGCC CTTATTTACG CTGCGCTAAT

RV_Vd_IL GGGATTTGCT TATCACATCT GGTGTGTCTT TTTTGGGGAG GGGCGTGTGT

Consensus GGttacaaaa TAatAgcTCa GtTcgaagcc cTTatttacG ctGCGctaaT

8301 8350

RV_Am_IL GACTCTAGAT GAAGGAGCTG ATGACCCAAA TTGTGAAAAT GCTTTTTTAG

RV_Bi_US GACTCTAGAT GAAGGAGCTG ATGACCCAAA TTGTGAAAAT GCTTTTTTAG

RV_Am_US GACTCTAGAT GAAGGAGCTG ATGACCCAAA TTGTGAAAAT GCTTTTTTAG

RV_Vd_IL GGGGGCAGAT ATTGGTGATA TACTGGGATG TGTTTGTGAC GGTGTTTTGT

Consensus GactctAGAT gaaGGaGcTg atgacccAaa TtgTgaaaAt GcTtTTTTag

8351 8400

RV_Am_IL ATAATATCCT GGATGATATC AAAAGTGTCA ATCCAGAAGA AGTTGTATTT

RV_Bi_US ATAATATCCT GGATGATATC AAAAGTGTCA ATCCAGAAGA AGTTGTATTT

RV_Am_US ATAATATCCT GGATGATATC AAAAGTGTCA ATCCAGAAGA AGTTGTATTT

RV_Vd_IL GGACTTGCCA GGCTAATTTT ATTTTGGAGT TTTGACGAGG GAGGTGGATC

Consensus atAaTatCCt GGaTgATaTc AaaagtGtca aTccAgaAGa agttgtatTt

8401 8450

RV_Am_IL GCTCGGGAAT TGTATAATTT CTTTTTAGGA CTATCTAACA AACATAAGCT

RV_Bi_US GCTCGGGAAT TGTATAATTT CTTTTTAGGA CTATCTAACA AACATAAGCT

RV_Am_US GCTCGGGAAT TGTATAATTT CTTTTTAGGA CTATCTAACA AACATAAGCT

RV_Vd_IL TTTGCGAAAT TCTTGATTTT TTGTTTAATG AGCCTGATTG TGCCGAGGTT

Consensus gcTcgGgAAT TgTatAaTTT cTtTTTAgga ctatctAaca aaCatAaGcT

8451 8500

RV_Am_IL ATCT...... GAACTATTCG GCCTTTTTAG ATTGTGGGGT CATCCAAACG

RV_Bi_US ATCT...... GAACTATTCG GCCTTTTTAG ATTGTGGGGT CATCCAAACG

RV_Am_US ATCT...... GAACTATTCG GCCTTTTTAG ATTGTGGGGT CATCCAAACG

RV_Vd_IL TTTTCGGTGT GATAGTTGTG TGGTTCTGGC ATTGCTTTGT GAGAGACATA

Consensus aTcT...... GAactaTtcG gccTTtTtag ATTGtgggGT cAtccAaAcg

8501 8550

RV_Am_IL TGTACGGCAG GGAAGGAACG CAAAAGATAA AAGAAGTTGG AAATAAGTCT

RV_Bi_US TGTACGGCAG GGAAGGAACG CAAAAGATAA AAGAAGTTGG AAATAAGTCT

RV_Am_US TGTACGGCAG GGAAGGAACG CAAAAGATAA AAGAAGTTGG AAATAAGTCT

RV_Vd_IL TTCTGGGGAT AATGAGACCA TAAAGATCAT AAGAATTATT ACAACTGTTT

Consensus TgtacGGcAg ggaagGAaCg cAAAagatAa AAGAAgTtgg AaAtaaGTcT

8551 8600

RV_Am_IL AAATACATAG ATCCTAAAGC AGTCGATTTG GTAACCAGTA ATTGGGTTAA

RV_Bi_US AAATATATAG ATCCTAAAGC AGTCGATTTG GTAACCAGTA ATTGGGTTAA

RV_Am_US AAATATATAG ATCCTAAAGC AGTCGATTTG GTAACCAGTA ATTGGGTTAA

RV_Vd_IL GTAGATAAAG TTCTAACTG. ....GATGGC TGGATACGGA GTGTCATTAT

Consensus aaAtAtAtAG aTCctAaaGc agtcGATttg gtaAccaGtA aTtgggTTAa

8601 8650

RV_Am_IL AATGTTTCTT AAAAATTATA TTAAGAAACA TAATCACTGG CCTCCTTGTT

RV_Bi_US AATGTTTCTT AAAAATTATA TTAAGAAACA TAATCACTGG CCTCCTTGTT

RV_Am_US AATGTTTCTT AAAAATTATA TTAAGAAACA TAATCACTGG CCTCCTTGTT

RV_Vd_IL CTTTGGTTTT GTGACGGGTC TGGTGAACTT TTGTCTATGT TTGACGGTCC

Consensus aaTgttTcTT aaaAattaTa TtaaGAAaca TaaTCacTGg cctcCttgtt

8651 8700

RV_Am_IL CTTTAGATCT ACCTGATAAT CACTACTTTA AAAACTGTTT AGATCTTGGA

RV_Bi_US CTTTAGATCT ACCTGATAAT CACTACTTTA AAAACTGTTT AGATCTTGGA

RV_Am_US CTTTAGATCT ACCTGATAAT CACTACTTTA AAAACTGTTT AGATCTTGGA

RV_Vd_IL TTTATCTTTG TCGCTTGAAG GGTTTCATTG TGGTTTGGCT TAAACTAGAT

Consensus cTTtagaTct aCctgatAAt cacTaCtTTa aaaacTGttT agAtCTtGga

8701 8750

RV_Am_IL ATATATCCAA ATGAACATCA CATAGAAAAC ACTAACAGTG CTTGGTTGCG

RV_Bi_US ATATATCCAA ATGAACATCA CATAGAAAAC ACTAACAGTG CTTGGTTGCG

RV_Am_US ATATATCCAA ATGAACATCA CATAGAAAAC ACTAACAGTG CTTGGTTGCG

RV_Vd_IL CGCTTGGAGC TTGCAGGGAA TTGGGATCGA GGGTGCTGTG CGGTATTCCG

Consensus ataTatccaa aTGaAcatcA cataGAaaac actaaCaGTG CttggTTgCG

8751 8800

RV_Am_IL CATTAGGTTT GAAAAGACTT TTTCTTCAAA TGACAAAATC TCTTTAGCAG

RV_Bi_US CATTAGGTTT GAAAAGACTT TTTCTTCAAA TGACAAAATC TCTTTAGCAG

RV_Am_US CATTAGGTTT GAAAAGACTT TTTCTTCAAA TGACAAAATC TCTTTAGCAG

RV_Vd_IL ATTTCGGGTG ..AGGGATTA CCTATTGATC AAAGCTATTT TATTTCCCAA

Consensus caTTaGGtTt gaAaaGAcTt ttTcTTcAaa tgAcaaAaTc TcTTTagCAg

8801 8850

RV_Am_IL AAATGATGGA GGATAAATCA TGTTCACCAA TTTTGTCAGA GTTACGAGAG

RV_Bi_US AAATGATGGA GGATAAATCA TGTTCACCAA TTTTGTCAGA GTTACGAGAG

RV_Am_US AAATGATGGA GGATAAATCA TGTTCACCAA TTTTGTCAGA GTTACGAGAG

RV_Vd_IL AAGTGGGGGT GTGCGGAGGG GGGGAATGGT GCTTCGCAGG AACATTC...

Consensus AAaTGatGGa GgataaAtca tGttcAccaa ttTTgtCAGa gttAcgagag

8851 8900

RV_Am_IL ACTTATAGAA GTCATAAGAG TGTGGGACTT GCAGAACAAA AAAGATTAAT

RV_Bi_US ACTTATAGAA GTCATAAGAG TGTGGGACTT GCAGAACAAA AAAGATTAAT

RV_Am_US ACTTATAGAA GTCATAAGAG TGTGGGACTT GCAGAACAAA AAAGATTAAT

RV_Vd_IL .CGTGTTGAT TTTCCTTTTG TGTTGAAAGT GAAGAAGTAG CTGGTTGCGT

Consensus aCtTaTaGAa gTcataagaG TGTgGgActT GcAGAAcaAa aaaGaTtaaT

8901 8950

RV_Am_IL A.......TT AAAATGGCTT GATTCACCCG ATATCTCGCC CCATGATTTT

RV_Bi_US A.......TT AAAATGGCTT GATTCACCCG ATATCTCGCC CCATGATTTT

RV_Am_US A.......TT AAAATGGCTT GATTCACCCG ATATCTCGCC CCATGATTTT

RV_Vd_IL TGGTGTACTT GTCTTGGCTC TCTGTCTTCT ATGTTGGCGG AGCGTGTGTA

Consensus a.......TT aaaaTGGCTt gaTtcaccCg ATaTctcgcc ccatgaTtTt

8951 9000

RV_Am_IL TTAGAGGAGA AGGACCTTAA GGGTTTCTCG AAAGAAGAGT TGATTAT...

RV_Bi_US TTAGAGGAGA AGGACCTTAA GGGTTTCTCG AAAGAAGAAT TGATTAT...

RV_Am_US TTAGAGGAGA AGGACCTTAA GGGTTTCTCG AAAGAAGAAT TGATTAT...

RV_Vd_IL TTTGCTTGTT CGGGTTGGAG AGATAGGGTT AAAGGAAACT TTAATACCGC

Consensus TTaGaggaga aGGaccttAa gGgTttctcg AAAGaAgAaT TgAtTAt...

9001 9050

RV_Am_IL ........TG GTCAAGTGTC AAAAGAACGA GAATTAAAAC TAGAGCCTAG

RV_Bi_US ........TG GTCAAGTGTC AAAAGAAAGA GAATTAAAAC TAGAGCCTAG

RV_Am_US ........TG GTCAAGTGTC AAAAGAAAGA GAATTAAAAC TAGAGCCTAG

RV_Vd_IL CATTGCCCTG GTCTTGTGTA TCTGGAACGA AACTTCCAAA CAAAAGTCAG

Consensus ........TG GTCaaGTGTc aaaaGAAaGA gAaTTaaAAc tAgAgcctAG

9051 9100

RV_Am_IL AATGTTTTCT TTAATGACT. TTCCCAGTGC GGCTTTATTT TGTATTAACA

RV_Bi_US AATGTTTTCT CTAATGACT. TTCCCAGTGC GGCTATATTT TGTATTAACA

RV_Am_US AATGTTTTCT CTAATGACT. TTCCCAGTGC GGCTATATTT TGTATTAACA

RV_Vd_IL ATGAATAACT TTTGCCACAA TTAATAAAAT AGCGAGAGTG ATGATAAACA

Consensus AatgtTttCT cTaatgACt. TTcccAgtgc gGCtatAtTt tgtATtAACA

9101 9150

RV_Am_IL GAAGCAATGA TAGCTAAATC AATATTACCG TACTTTAAAG AAATAACTAT

RV_Bi_US GAAGCAATGA TAGCTAAATC AATATTACCG TATTTTAAAG AAATAACTAT

RV_Am_US GAAGCAATGA TAGCTAAATC AATATTACCG TATTTTAAAG AAATAACTAT

RV_Vd_IL AAGGTAAGG. ..GCGAGGTT ATTTTATGCC AAATCCCCAC AAAGAACTTC

Consensus gAaGcAAtGa taGCtAaaTc AaTaTtacCg tAtTttaaAg AAAtAACTat

9151 9200

RV_Am_IL AACATATAAG GATCTGGAAC TAAAAA.... .......... .......AAC

RV_Bi_US AACATATAAG GATCTGGAAC TGAAAA.... .......... .......AAC

RV_Am_US AACATATAAG GATCTGGAAC TGAAAA.... .......... .......AAC

RV_Vd_IL GATATAGGTG TCCAGGGGTC CCTAGGGTTT ATAACACCGG GGATTTCATC

Consensus aAcATAtaaG gatctGGaaC tgaAaa.... .......... .......AaC

9201 9250

RV_Am_IL GCTTAATTAA TTTAACAAGG GATCAAGCTA GATCGCCAGG TAAGAGGAAA

RV_Bi_US GCTTAATTAA TTTAACAAGG GATCAAGCTA GATCGCCAGG TAAGAGGAAA

RV_Am_US GCTTAATTAA TTTAACAAGG GATCAAGCTA GATCGCCAGG TAAGAGGAAA

RV_Vd_IL CATTTGATAA TTCAGTAACG TTGTTGTCAA AGCCTCTAAT CTTTTGTTAA

Consensus gcTTaatTAA TTtAacAAgG gatcaagCtA gatCgCcAgg taagaGgaAA

9251 9300

RV_Am_IL TGTACATATA TAACCATAAA TATGGATTTT GAAAAATGGA ACCACAATAT

RV_Bi_US TGTACATATA TAACCATAAA TATGGATTTT GAAAAATGGA ACCACAATAT

RV_Am_US TGTACATATA TAACCATAAA TATGGATTTT GAAAAATGGA ACCACAATAT

RV_Vd_IL TTTTGCTAAT TGA..ATACC TGCTGAATTC GTCTTCTTGA AGCTCATGTA

Consensus TgTacaTAta TaAccATAaa TatgGAtTTt GaaaaaTgGA AcCaCAatat

9301 9350

RV_Am_IL GCGTGATAGA CTGGTAAGAC CAATATTTGA AAGATTGGAT CAATTGTTTG

RV_Bi_US GCGTGATAGA CTGGTAAGAC CAATATTTGA AAGATTGGAT CAATTGTTTG

RV_Am_US GCGTGATAGA CTGGTAAGAC CAATATTTGA AAGATTGGAT CAATTGTTTG

RV_Vd_IL GACTGTATAG GTCAGAAGGC GCAAATTGTA AGGGATGCAT TACAAGGTGC

Consensus GcgTGataga cTggtAAGaC caAtATTtgA AaGatTGgAT cAattGtTtg

9351 9400

RV_Am_IL GCTTTAATC. .......... ...ATCTAAT ATCACGAACT CATGATATTT

RV_Bi_US GCTTTAATC. .......... ...ATCTAAT ATCACGAACT CATGATATTT

RV_Am_US GCTTTAATC. .......... ...ATCTAAT ATCACGAACT CATGATATTT

RV_Vd_IL GACATAGAAG ACAGCACTGG TCGATCTAAA ATATAAAGCT TATGATTGTC

Consensus GcttTAatc. .......... ...ATCTAAt ATcacgAaCT cATGATatTt

9401 9450

RV_Am_IL TCAAAGAATC TCACTATTAT TTAGCAGAAG ATGGAACTGA ACTAGAATTC

RV_Bi_US TCAAAGANTC TCACTATTAT TTAGCAGAAG ATGGAACTGA ACTAGAATTC

RV_Am_US TCAAAGANTC TCACTATTAT TTAGCAGAAG ATGGAACTGA ACTAGAATTC

RV_Vd_IL TAATCCCAAA TAGTGGGTCT CGTTGATGTA GATTAAATGT CCTTCACTTA

Consensus TcAaagaatc TcactatTaT ttagcAgaag atggAAcTGa aCTagAaTTc

9451 9500

RV_Am_IL AACATGCGAG GAGATCTAAA AGACAATATT TGCTCGTAT. .......AAA

RV_Bi_US AACATGCGAG GAGATCTAAA AGACAATATT TGCTCGTAT. .......AAA

RV_Am_US AACATGCGAG GAGATCTAAA AGACAATATT TGCTCGTAT. .......AAA

RV_Vd_IL GGGAACAATG ATCATCTGTG GTACCTCTAC TGATGGTATT TGTAGCAATC

Consensus aacAtgcgaG gagATCTaaa agACaatatt TGcTcGTAT. .......Aaa

9501 9550

RV_Am_IL GGAACAAAAG GTGGTCAGGA AGGTCTTCGA CAAAAAGGTT GGACACTGGT

RV_Bi_US GGAACAAAAG GTGGTCAGGA AGGTCTTCGA CAAAAAGGTT GGACACTGGT

RV_Am_US GGAACAAAAG GTGGTCAGGA AGGTCTTCGA CAAAAAGGTT GGACACTGGT

RV_Vd_IL TCTAAAAAGT CAAGGCTATT TTTAATTGGC CAAAATTTCC CGCATCGAGT

Consensus ggaAcAAAag gtgGtCagga aggtcTTcGa CAAAAaggtt gGacaCtgGT

9551 9600

RV_Am_IL CACAGTAGCA GCAATTTATA CCGTTGTTTC TACAACACCT CATAGTTTTC

RV_Bi_US CACAGTAGCA GCAATTTATA CCGTTGTTTC TACAACACCT CATAGTTTTC

RV_Am_US CACAGTAGCA GCAATTTATA CCGTTGTTTC TACAACACCT CATAGTTTTC

RV_Vd_IL AGGATTGGAG TCAAGGTAAA CCCGAACTTT GATACAATTT CGAACAATCA

Consensus cacAgTaGca gCAAttTAtA CCgttgtTTc tAcAacAccT CatAgttTtc

9601 9650

RV_Am_IL AACTGTCAGG ACAAGGTGAT AATCAAGTTA TTATTCTGGC AATTCATGAT

RV_Bi_US AACTGTCGGG ACAAGGTGAT AATCAAGTTA TTATTCTGGC AATTCATGAT

RV_Am_US AACTGTCGGG ACAAGGTGAT AATCAAGTTA TTATTCTGGC AATTCATGAT

RV_Vd_IL ATC...CACC ACGCAGATAG CTGGCGGTTA TTTCTCTTTC ATTAAAGAAT

Consensus AaCtgtCagg ACaagGtgAt aatcaaGTTA TTatTCTggC AaTtcAtgAT

9651 9700

RV_Am_IL CCTCCAGGTT .......... ........CA AATCTCAATG ATGCTGAAAG

RV_Bi_US CCTCCAGGTT .......... ........CA AATCTCAATG ATGCTGAAAG

RV_Am_US CCTCCAGGTT .......... ........CA AATCTCAATG ATGCTGAAAG

RV_Vd_IL ATTTTAGCTG ATTCAGTAGG GGTAACACCT AATAATGACC TAGCTAAAAA

Consensus ccTccAGgTt .......... ........Ca AATctcaAtg atGCTgAAAg

9701 9750

RV_Am_IL AGCGACATTT GTTCGAAATG AGGTTAG..G ATGATTCGTG CCAATCTATC

RV_Bi_US AGCGACATTT GTTCGAAATG AGGTTAG..G ATGATTCGTG CCAATCTATC

RV_Am_US AGCGACATTT GTTCGAAATG AGGTTAG..G ATGATTCGTG CCAATCTATC

RV_Vd_IL CCACTCATTC TCTTTAATAG TGCATATCTG ATTTTGGATA CTGTCCCATA

Consensus agcgaCATTt gtTcgAAatG aGgtTAg..G ATgaTtcgTg CcaatCtATc

9751 9800

RV_Am_IL GACTTTTTTT ACTAAAATTG GATTACCCTT AAAAACCGCA GAGACCTGGA

RV_Bi_US GACTTTTTTT ACTAAAATTG GATTACCCTT AAAAACCGCA GAGACCTGGA

RV_Am_US GACTTTTTTT ACTAAAATTG GATTACCCTT AAAAACCGCA GAGACCTGGA

RV_Vd_IL TTCGCTCTAT ACTAATTTGT AAATTATCTT GCAAA..... .........A

Consensus gaCttTtTtT ACTAAaaTtg gAtTaccCTT aaAAAccgca gagacctggA

9801 9850

RV_Am_IL CATCTTCAAT TTTATTTGCT TATGGTAAGA AATTATTGAG AATGGGTGTA

RV_Bi_US CATCTTCAAT TTTATTTGCT TATGGTAAGA AATTATTGAG AATGGGTGTA

RV_Am_US CATCTTCAAT TTTATTTGCT TATGGTAAGA AATTATTGAG AATGGGTGTA

RV_Vd_IL ATTCTAACAT ACTGGACGCT AACGTCAATG CAAGACTGGG CATGGTCTCA

Consensus caTCTtcaAT ttTatttGCT tAtGgtAAga aAttAtTGaG aATGGgtgtA

9851 9900

RV_Am_IL CTTTTAA.CT ATGTTTCTTA AAAGGGTGTC GAGAACTTTT CCTTTCTCAA

RV_Bi_US CTTTTAA.CT ATGTTTCTTA AAAGGGTGTC GAGAACTTTT CCTTTCTCAA

RV_Am_US CTTTTAA.CT ATGTTTCTTA AAAGGGTGTC GAGAACTTTT CCTTTCTCAA

RV_Vd_IL GTAACCGGCA AAAGATCCCA AAACGCCTTT TCAGTCATGT TTTTCCTGAA

Consensus cTtttaa.Ct AtgttTCttA AAAgGgtgTc gagaaCtTtT ccTTtCTcAA

9901 9950

RV_Am_IL ATGAAAAATT ACCTTCTCTT GAGAGTGACA TA........ GCAGCTGTCT

RV_Bi_US ATGAAAAATT ACCTTCTCTT GAGAGTGACA TA........ GCAGCTGTCT

RV_Am_US ATGAAAAATT ACCTTCTCTT GAGAGTGACA TA........ GCAGCTGTCT

RV_Vd_IL AATTCTAAGC ATCAGGCCTT CTGAGGTGTA AAAAATACGT GAAGTGTTAG

Consensus AtgaaaAAtt AcCttctCTT gaGAGtgacA tA........ GcAGctgTct

9951 10000

RV_Am_IL GGGCAAATAC TGCCGCTGCT GCTGAGTTTG ATTTTTTCCC ATTCAATTCT

RV_Bi_US GGGCAAATAC TGCCGCTGCT GCTGAGTTTG ATTTTTTCCC ATTCAATTCT

RV_Am_US GGGCAAATAC TGCCGCTGCT GCTGAGTTTG ATTTTTTCCC ATTCAATTCT

RV_Vd_IL TGGCACAGAC TTCCAAAGGG CCAACCGGAC GGTACTCCTG ATTCAAATGG

Consensus gGGCAaAtAC TgCCgctGct gCtgagtttg atTttTtCcc ATTCAAtTct

10001 10050

RV_Am_IL TTGACAAT.T GGTTCTTATC AAACGGGTTG GGCACTTTTG ACTCATCTTG

RV_Bi_US TTGACAAT.T GGTTCTTATC AAACGGGTTG GGCACTTTTG ACTCATCTTG

RV_Am_US TTGACAAT.T GGTTCTTATC AAACGGGTTG GGCACTTTTG ACTCATCTTG

RV_Vd_IL AAAATATGAT GGTTGTTTGG ACAGACAATA ATGAGATTGG ACAAATCACG

Consensus ttgAcAat.T GGTTcTTatc AaAcgggtTg ggcActTTtG ACtcATCttG

10051 10100

RV_Am_IL AATTTTCGCC CATTTTGGAG ATCGGTCTTC TTCAGGCCTG TTTAGACAAC

RV_Bi_US AATTTTCGCC CATTTTGGAG ATCGGTCTTC TTCAGGCCTG TTTAGACAAC

RV_Am_US AATTTTCGCC CATTTTGGAG ATCGGTCTTC TTCAGGCCTG TTTAGACAAC

RV_Vd_IL GGATGGAACA ...TTTGGTG ATAAGTCAGC TCGAAC.... TGTACATAAC

Consensus aatTttcgCc catTTTGGaG ATcgGTCttC TtcAggcctg TtTAgAcAAC

10101 10150

RV_Am_IL GGAGACTTTG GAACGAATCA TAGGAGAAAG AAAATAAGGA TTCCAAAC..

RV_Bi_US GGAGACTTTG GCACGAATCA TAGGAGAAAG AAAATAAAGA TTCCAAAT..

RV_Am_US GGAGACTTTG GCACGAATCA TAGGAGAAAG AAAATAAAGA TTCCAAAT..

RV_Vd_IL TCAGTTTGAA AATGCTCTAA AGGGCAAGAG ATATTAATGT TTGCATTCCA

Consensus ggAGacTttg gaacgaaTcA taGGagAaAG AaAaTAAaGa TTcCAaac..

10151 10200

RV_Am_IL .........A CGCTTGTTAG ATCTCTATTA ATTCAGAACT TGGAGAAAGT

RV_Bi_US .........A CGCTTGTTAG ATCTCTATTA ATTCAGAACT TGGAGAAAGT

RV_Am_US .........A CGCTTGTTAG ATCTCTATTA ATTCAGAACT TGGAGAAAGT

RV_Vd_IL CACATATTCA ACCCCGTCGG CTGTCATTAA GTAACCAAGA TGCAAGGGGA

Consensus .........A cgCttGTtaG aTcTCtaTtA aTtcagAAct TGgAgaaaGt

10201 10250

RV_Am_IL AATTTTTGGA ATTCTAGTCA GGCCCAAAAT ACTTGGCGGG TATCCTATTT

RV_Bi_US AATTTTTGGA ATTCTAGTCA GGCCCAAAAT ACTTGGAGGG TATCCTATTT

RV_Am_US AATTTTTGGA ATTCTAGTCA GGCCCAAAAT ACTTGGAGGG TATCCTATTT

RV_Vd_IL TACCTTTTGT ATTCGGGATT .......... ..TCAATGTG AACATTAGAT

Consensus aAttTTTgGa ATTCtaGtca ggcccaaaat acTtggaGgG tAtccTAttT

10251 10300

RV_Am_IL CTCTTTTAGA TGAGTTAACG ATTAAAGGTA TACCAGATCC ACTG......

RV_Bi_US CTCTTTTAGA TGAGTTAACG ATTAAAGGTA TACCAGATCC ACTG......

RV_Am_US CTCTTTTAGA TGAGTTAACG ATTAAAGGTA TACCAGATCC ACTG......

RV_Vd_IL CCGGTGACAT TTAATAAATG ATCATTTCTA TAGCAGACTC TTTTGCTATA

Consensus CtctTttaga TgAgTtAAcG ATtAaaggTA TAcCAGAtcC acTg......

10301 10350

RV_Am_IL .......TCA TCAGGATTAC ACCAGTTGAA GCGAATGTTA CCTTATGCAT

RV_Bi_US .......TCA TCAGGATTAC ACCAGTTGAA GCGAATGTTA CCTTATGCAT

RV_Am_US .......TCA TCAGGATTAC ACCAGTTGAA GCGAATGTTA CCTTATGCAT

RV_Vd_IL TGTTACTTCA TTCTCTTCCA TCCAGTGGCA CTCAAAATCT CTTTTGGTCC

Consensus .......TCA TcaggaTtac aCCAGTtGaA gcgAAtgTta CcTTatGcat

10351 10400

RV_Am_IL CAAGTTGGCA G...TCGGCA ATTATAAGAC GAGCTATTTG TCCTTTATTA

RV_Bi_US CAAGTTGGCA G...TCGGCA ATTATAAGAC GAGCTATTTG TCCTTTATTA

RV_Am_US CAAGTTGGCA G...TCGGCA ATTATAAGAC GAGCTATTTG TCCTTTATTA

RV_Vd_IL CAAAGGAGAA GGTATCCTCG ATGGTCTTAA CACTTTTTAA AGCTGCAATT

Consensus CAAgttgGcA G...TCggCa ATtaTaagAc gAgcTaTTtg tcCTttAtTa

10401 10450

RV_Am_IL TCACAATACG TCAAACCTAT TATGTTATAT CAAGATCCAT TGTCCATTAA

RV_Bi_US TCACAATACG TCAAACCTAT TATGTTATAT CAAGATCCAT TGTCCATTAA

RV_Am_US TCACAATACG TCAAACCTAT TATGTTATAT CAAGATCCAT TGTCCATTAA

RV_Vd_IL TCCAAAAAGT TTAACGGAAC TATCTTTTTT GACTTGCGGG TAAACTCGAA

Consensus TCacAAtAcg TcAAacctAt TATgTTaTaT cAagatCcat TgtcCattAA

10451 10500

RV_Am_IL CATTATTTCA CCACCAAGTG GTGAATCGGT ACTTAAGGAT ATATCGACCA

RV_Bi_US CATTATTTCA CCACCAAGTG GCGAATCAGT ACTCAAGGAT ATATCGACCA

RV_Am_US CATTATTTCA CCACCAAGTG GCGAATCAGT ACTCAAGGAT ATATCGACCA

RV_Vd_IL TTGGTGTTGA AATCCAAAAA AGGTCTCTTT ACACACTTTA ATAATCTCAA

Consensus cattatTTcA ccaCCAAgtg gcGaaTCagT ACtcAaggat ATAtcgaCcA

10501 10550

RV_Am_IL AAGCTGTCAG AACCATGGCT ATTAAAAACA AAACTATTAG AGAGTGTTTG

RV_Bi_US AAGCTGTCAG AACCATGGCT ATTAAAAACA AAACTATTCG AGAGTGTTTG

RV_Am_US AAGCTGTCAG AACCATGGCT ATTAAAAACA AAACTATTCG AGAGTGTTTG

RV_Vd_IL AC........ .ATCTCACAC CTCCAAGCTG AAACTCTTTG ATGAGATGGG

Consensus Aagctgtcag aAcCatggct aTtaAAaaca AAACTaTTcG AgagtgTttG

10551 10600

RV_Am_IL CGTTATGAAG ATAGTGA... .........G CTTATAGAAC TAAGTCAATC

RV_Bi_US CGTTATGAAG ATAGTGA... .........G CTTATAGAAC TAAGTCAATC

RV_Am_US CGTTATGAAG ATAGTGA... .........G CTTATAGAAC TAAGTCAATC

RV_Vd_IL ATTTGTCTCA ATATTTCAAG TTCGATCTGG CCAATCGTTT TATATGTGTT

Consensus cgTTaTgaag ATAgTga... .........G CttATaGaac TAagTcaaTc

10601 10650

RV_Am_IL ATTGTGGGCA TGTGAACCTC AGTATCCATT TTTAATGTCT GACATATTAT

RV_Bi_US ATTGTGGGCA TGTGAACCTC AGTATCCATT TTTAATGTCT GACATATTAT

RV_Am_US ATTGTGGGCA TGTGAACCTC AGTATCCATT TTTAATGTCT GACATATTAT

RV_Vd_IL TTTGTTGATA TCCAGATAAC ATGATGGTTT TTTCGGAATT GGCAAAAATC

Consensus aTTGTgGgcA TgtgaAcctC AgtATccaTT TTTaatgtcT GaCAtAttat

10651 10700

RV_Am_IL CTTCATCACT GGTGGGATAC CAAAAGGAAT TAACTGCCAA GTGTTCTCAA

RV_Bi_US CTTCATCACT GGTGGGATAC CAAAAGGAAT TAACCGCCAA GTGTTCTCAA

RV_Am_US CTTCATCACT GGTGGGATAC CAAAAGGAAT TAACCGCCAA GTGTTCTCAA

RV_Vd_IL GAGATGCTCC GCT...ACAT CTTAAGGCTT GAACCGGTAA ATTATCTAAA

Consensus cttcatCaCt GgTgggAtAc CaaAAGGaaT tAACcGccAA gTgtTCTcAA

10701 10750

RV_Am_IL ACAACAACAA TGCGTGCAAG AGCAGCAAAA TCAGTTTTAA GTACAGAGAG

RV_Bi_US ACAACAACAA TGCGTGCAAG GGCAGCAAAA TCAGTTTTAA GTACAGAGAG

RV_Am_US ACAACAACAA TGCGTGCAAG GGCAGCAAAA TCAGTTTTAA GTACAGAGAG

RV_Vd_IL AGACTCAGAT CCAGTTCCTC AGCTAGTTGA TAAGTGAAAG CTGGTAAACA

Consensus AcAacaAcAa tgcGTgCaag aGCagcaaaA TcAGTtttAa gTacagAgag

10751 10800

RV_Am_IL TTACCGGATT AA....AAAA TACGAAAGAA CCTCGTTTTT ACAAGCATTA

RV_Bi_US TTACCGGATT AA....AAAA TACGAAAGAA CCTCGTTTTT ACAAGCATTA

RV_Am_US TTACCGGATT AA....AAAA TACGAAAGAA CCTCGTTTTT ACAAGCATTA

RV_Vd_IL ACATAGGGTT AATCCCAAAG CAGCTAAAAA CTTGCTGTTG ATCACAGACA

Consensus ttAccGGaTT AA....AAAa tAcgaAAgAA CcTcgTtTTt AcaAgcattA

10801 10850

RV_Am_IL TGGGCTCAAC ATCTAACAGA ACCTTTTGAT CATTTTACTA GTAATTTCAA

RV_Bi_US TGGGCTCAAC ATCTAACAGA ACCTTTTGAT CATTTTACTA GTAATTTCAA

RV_Am_US TGGGCTCAAC ATCTAACAGA ACCTTTTGAT CATTTTACTA GTAATTTCAA

RV_Vd_IL GGGTGTCAGC TTGTCTCGTT .......... ..TTTGACCT TTCGGTCAGC

Consensus tGGgcTCAaC aTcTaaCaga accttttgat caTTTtACta gTaatTtcaa

10851 10900

RV_Am_IL TTGCAGTAGA ATGGCTGCAC ATCAGATTAG AGAAGTTAGT TGGGGACGAC

RV_Bi_US TTGCAGTAGA ATGGCTGCAC ATCAGATTAG AGAAGTTAGT TGGGGACGAC

RV_Am_US TTGCAGTAGA ATGGCTGCAC ATCAGATTAG AGAAGTTAGT TGGGGACGAC

RV_Vd_IL TTGCAGTGCA TAGTATGAGC CATCGTTGCG TTTTGGTAAT GGAGTGTGGT

Consensus TTGCAGTagA atGgcTGcaC atcaGaTtaG agaaGtTAgT tGgGgacGac

10901 10950

RV_Am_IL CAATAGTCGG TGTAACTGTA CCAAGTCCTT TTGAAGTTTT GTCTGCGAAA

RV_Bi_US CAATAGTCGG TGTAACTGTA CCAAGTCCTT TTGAAGTTTT GTCTGCGAAA

RV_Am_US CAATAGTCGG TGTAACTGTA CCNAGTCCTT TTGAAGTTTT GTCTGCGAAA

RV_Vd_IL ATGCGTTCGG GGACTCTGAT TTTGGACAAA TGGTTCCTTG AT........

Consensus caatagTCGG tGtaaCTGta ccaaGtCctt TtGaagtTTt gTctgcgaaa

10951 11000

RV_Am_IL GAAGACGGTC GTCACTGCAA AGACAGAAAT AGTGGTGATT ACATTCAAGT

RV_Bi_US GAAGACGGTC GTCACTGCAA AGACAGAAAT AGTGGTGATT ATATTCAAGT

RV_Am_US GAAGACGGTC GTCACTGCAA AGACAGAAAT AGTGGTGATT ATATTCAAGT

RV_Vd_IL .......... .......... .......... .GTGTCGTTG ACTAGATCTT

Consensus gaagacggtc gtcactgcaa agacagaaat aGTGgtGaTt AcattcaagT

11001 11050

RV_Am_IL TATAGTCAGT CCTGATATAT ATGAAAAGTG GAATTCTTTT GATGTCTGTG

RV_Bi_US TATAGTCAGT CCTGATATAT ATGAAAAGTG GAATTCTTTT GATGTCTGTG

RV_Am_US TATAGTCAGT CCTGATATAT ATGAAAAGTG GAATTCTTTT GATGTCTGTG

RV_Vd_IL TAGAGTAACT CGGATTTAAA TTACTACCGG GCCGAGCTGT ATTGCTGGAT

Consensus TAtAGTcAgT CctgaTatAt aTgaaAagtG GaattctTtT gaTGtctGtg

11051 11100

RV_Am_IL GACCCTTTTC GCCTTACCTC GGCTCAGAGA CAAAGGATAA AACTCATAGT

RV_Bi_US GACCCTTTTC GCCTTACCTC GGCTCAGAGA CAAAGGATAA AACTCATAGT

RV_Am_US GACCCTTTTC GCCTTACCTC GGCTCAGAGA CAAAGGATAA AACTCATAGT

RV_Vd_IL CTACGGTTTC GAATCACTAT GACGCAGATG TTGATTGTTA AGGCTTGCTT

Consensus gacCctTTTC GccTtACctc GgCtCAGAga caaAggaTaA AactcatagT

11101 11150

RV_Am_IL TATCAAAACA GTATCTCTTA TATCGTTGAA TCAGTAGCCA AGAAGGCAAT

RV_Bi_US TATCAAAACA GTATCTCTTA TATCGTTGAA TCAGTAGCCA AGAAGGCAAT

RV_Am_US TATCAAAACA GTATCTCTTA TATCGTTGAA TCAGTAGCCA AGAAGGCAAT

RV_Vd_IL TATCTGAGGT TGGTTTGGAC TGTTGTATTG AATGGACCCG AAAAGTTAAT

Consensus TATCaaAaca gtaTcTctta TaTcGTtgaa tcaGtAgCCa AgAAGgcAAT

11151 11200

RV_Am_IL CAAACTTTCT AGAGCCAT.. .........T GGTTGGTTCA CTAATGCCAC

RV_Bi_US CAAACTTTCT AGAGCCAT.. .........T GGTTGGTTCA CTAATGCCAC

RV_Am_US CAAACTTTCT AGAGCCAT.. .........T GGTTGGTTCA CTAATGCCAC

RV_Vd_IL GCAGTTTTTC AGCTCTCTCA AATTTTCTAT GCTTTCGTGA GCAATGGTTT

Consensus caAacTTTct AGagCcaT.. .........T GgTTggtTcA ctAATGccac

11201 11250

RV_Am_IL TTCACCCTTG GGGCACGCCT TACAACGGCT AGCTCAATCA GTGACAGATA

RV_Bi_US TTCACCCTTG GGGCATGCCT TACAACGGCT AGCTCAATCA GTGACAGATA

RV_Am_US TTCACCCTTG GGGCATGCCT TACAACGGCT AGCTCAATCA GTGACAGATA

RV_Vd_IL TTTACCACTA TCGGTTCTAA ACATTCTTTT TTAATAATTA GTGTTTATTA

Consensus TTcACCctTg ggGcatgcct tacaaCggcT agctcAATcA GTGacagaTA

11251 11300

RV_Am_IL TTCCATT... .....AGAAA ATTTCACCAC AGTGCTAGAG AATCACTCAG

RV_Bi_US TTCCATT... .....AGAAA ATTTCACCAC AGTGCTAGAG AATCACTCAG

RV_Am_US TTCCATT... .....AGAAA ATTTCACCAC AGTGCTAGAG AATCANTCAG

RV_Vd_IL ATCAATTTCT TTAATAGACA TTGTTATTTT ATTTTGAGTA AGAGATCTAT

Consensus tTCcATT... .....AGAaA aTtTcAccac AgTgctAGag AatcActcAg

11301 11350

RV_Am_IL GAAGTCCTTT TCATAGATAT AATGGTGGAA GAAATA.... ..........

RV_Bi_US GAAGTCCTTT TCATAGATAT AATGGTGGAA GAAATA.... ..........

RV_Am_US GAAGTCCTTT TCATAGATAT AATGGTGGAA GAAATA.... ..........

RV_Vd_IL CGAATGTTTA TATTTGATAC AAATTTTTGA AAAATGAGAT CTAAAAAATT

Consensus gaAgTccTTt TcaTaGATAt AAtggTggaA gAAATa.... ..........

11351 11400

RV_Am_IL ........TA CGAATTGGTA GATATAACCT CAATCAGCAA CCTTGTACCT

RV_Bi_US ........TA CGAATTGGTA GATATAACCT CAATCAGCAA CCTTGTACCT

RV_Am_US ........TA CGAATTGGTA GATATAACCT CAATCAGCAA CCTTGTACCT

RV_Vd_IL ATTGCTGTTA GAAGTTTGTA ATTTTCTCCG TTACATAGAG GTTTTTCGTG

Consensus ........TA cgAaTTgGTA gaTaTaaCCt caAtcagcAa ccTTgTacct

11401 11450

RV_Am_IL ATATGTCAGT AAATACAGAC CAA....TTG TCTGAATTTT CAAAGGGCTC

RV_Bi_US ATATGTCAGT AAATACAGAC CAA....TTG TCTGAATTTT CAAAGGGCTC

RV_Am_US ATATGTCAGT AAATACAGAC CAA....TTG TCTGAATTTT CAAAGGGCTC

RV_Vd_IL ATATTAAGGA AATCATAGAG AAAAGCATTA TCAGATTATT GCTGGTCTCT

Consensus ATATgtcaGt AAatAcAGAc cAA....TTg TCtGAaTtTT caaaGggctc

11451 11500

RV_Am_IL ACA....AAA CACGACAATA CATTTTCAAA CGTTGATGTT GTCAGCCCAA

RV_Bi_US ACA....AAA CACGACAATA CATTTTCAAA CGTTGATGTT GTCAGCCCAA

RV_Am_US ACA....AAA CACGACAATA CATTTTCAAA CGTTGATGTT GTCAGCCCAA

RV_Vd_IL ATTGCGGAAA GAAGGGAAGA TTACTACAAT GGCATAGGTG GTTGAGGAGA

Consensus Aca....AAA cAcGacAAtA cattTtCAAa cGttgAtGTt GTcagcccaA

11501 11550

RV_Am_IL GTTAAAGTAT TGTATGAAAT TGCGCATTAT GCGTTAACTG GAGAGAAGAA

RV_Bi_US GTTAAAGTAT TGTATGAGAT TGCGCATTAT GCGTTAACTG GAGAGAAGAA

RV_Am_US GTTAAAGTAT TGTATGAGAT TGCGCATTAT GCGTTAACTG GAGAGAAGAA

RV_Vd_IL AGAATTGGGT TTTTTGGTCT AGAG..TGAG GTTTTGCTTG GATTGGATGA

Consensus gttAaaGtaT TgTaTGagaT tGcGcaTtAt GcgTTaacTG GAgaGaAgaA

11551 11600

RV_Am_IL GAAGATACTG GATTTTCATT TTCATATAAA CTGTCGGGAA TGTATCGAAT

RV_Bi_US GAAGATACTG GATTTTCATT TTCATATAAA CTGTCGGGAA TGTATCGAAT

RV_Am_US GAAGATACTG GATTTTCATT TTCATATAAA CTGTCGGGAA TGTATCGAAT

RV_Vd_IL TTTGTTCTTA AAGGCTCCTT TTGGCTAAAA CCGGTCAGAA GGATATGGAT

Consensus gaaGaTacTg gAtttTCaTT TTcatatAAA CtGtcggGAA tGtatcGaAT

11601 11650

RV_Am_IL ATATTAGCAA TGATTTTATT GAGGG..... .......TGA TGACAAATGG

RV_Bi_US ATATTAGCAA TGATTTTATT GAGGG..... .......TGA TGACAAATGG

RV_Am_US ATATTAGCAA TGATTTTATT GAGGG..... .......TGA TGACAAATGG

RV_Vd_IL GGATGAGTAG GTCTATTGTT TTCGGGGTCA ATGTTTTTGG GGCCTTATGG

Consensus atATtAGcAa tgaTtTTaTT gagGG..... .......TGa tGaCaaATGG

11651 11700

RV_Am_IL CACTTAATCA GATTTAAATC ATGCCCCAGC AATTATTATT TATTTCAAAG

RV_Bi_US CACTTAATCA GATTTAAATC ATGCCCCAGC AATTATTATT TATTTCAAAG

RV_Am_US CACTTAATCA GATTTAAATC ATGCCCCAGC AATTATTATT TATTTCAAAG

RV_Vd_IL CATGGGTTCA CCTTGAATGC CTAAGTGGGA CATGGGGATC AGTTT..AAG

Consensus CActtaaTCA gaTTtAAatC aTgccccaGc aATtattATt taTTTcaAAG

11701 11750

RV_Am_IL TTTAGAAGCC ACTAGGATCG AAAAAGTAAG .......... AAGTGTAATC

RV_Bi_US TTTAGAAGCC ACTAGGATCG AAAAAGTAAG .......... AAGTGTAATC

RV_Am_US TTTAGAAGCC ACTAGGATCG AAAAAGTAAG .......... AAGTGTAATC

RV_Vd_IL ACGAGCATCA GCTATGCCGG TAACGGAATT TTGCAAGGTA AAATGTAATG

Consensus tttAGaAgCc aCTAgGatcG aAAaaGtAag .......... AAgTGTAATc

11751 11800

RV_Am_IL GATCATGAAA TATGTCTGGG CTCCTCGCTT ACTGTGACAG AGAGAAGATC

RV_Bi_US GATCATGAAA TATGTCTGGG CTCCTCGCTT ACTGTGACAG AGAGAAGATC

RV_Am_US GATCATGAAA TATGTCTGGG CTCCTCGCTT ACTGTGACAG AGAGAAGATC

RV_Vd_IL GAAC.TGCAA TATCGGTGTA AACCTGGACT ATCTTTTTAA AGTAA.....

Consensus GAtCaTGaAA TATgtcTGgg ctCCTcGctT ActgTgacAg AGagAagatc

11801 11850

RV_Am_IL TTTAGCTTCT GTTGCACTTG GGTCAATAAT CTTACGAGAT CGTGATTTGG

RV_Bi_US TTTAGCTTCT GTTGCACTTG GGTCAATAAT CTTACGAGAT CGTGATTTGG

RV_Am_US TTTAGCTTCT GTTGCACTTG GGTCAATAAT CTTACGAGAT CGTGATTTGG

RV_Vd_IL .TAGGTACCT GTTTTACTTG GCCCGAATTG CATGAGTGAC CTTCTTATGT

Consensus tTtaGcttCT GTTgcACTTG GgtCaAtaat CtTacGaGAt CgTgaTtTGg

11851 11900

RV_Am_IL CTCAAGGCGA TGTTAACGAT AATAATGCTT TAATTCCGTG GGTCTGGCTT

RV_Bi_US CTCAAGGCGA TGTTAACGAT AATAATGCTT TAATTCCGTG GGTCTGGCTT

RV_Am_US CTCAAGGCGA TGTTAACGAT AATAATGCTT TAATTCCGTG GGTCTGGCTT

RV_Vd_IL ACTTTGGGGG AAATATGGGT GCCAAAT... .......... .GACACACCT

Consensus ctcaaGGcGa tgtTAacGaT aatAAtgctt taattccgtg gGtCtggCtT

11901 11950

RV_Am_IL GCATACTTGA GCC..CGGTG TGTATATTAG AAGGTATAGT CGTTTATGCG

RV_Bi_US GCATACTTGA GCC..CGGTG TGTATATTAG AAGGTATAGT AGTTTATGCG

RV_Am_US GCATACTTGA GCC..CGGTG TGTATATTAG AAGGTATAGT AGTTTATGCG

RV_Vd_IL GCTTATCGAA GTCGTCAATG GCACCATCTA GTGCTCTCTT TGCTGCGTCA

Consensus GCaTActtgA GcC..CggTG tgtatATtag aaGgTaTagT aGtTtatgCg

11951 12000

RV_Am_IL TTGGTTAAGG CAATTATTAC TGTGACAAAA GATTATCATT ATCTATCTAA

RV_Bi_US TTGGTTAAGG CAATTATTAC TGTGACAAAA GATTATCATT ATCTATCTAA

RV_Am_US TTGGTTAAGG CAATTATTAC TGTGACAAAA GATTATCATT ATCTATCTAA

RV_Vd_IL TATGAGGGTG GACAATACAT AGACACTGAT AGTTCCCATT TTAAAGGAAT

Consensus TtgGttaagG cAattattAc tGtgACaaAa gaTTatCATT aTctAtctAa

12001 12050

RV_Am_IL GATCCCTTCA AATGTTATTT ATCAGAAAAC AGCTGAAATT CTAGAAGCAT

RV_Bi_US GATCCCTTCA AATGTTATTT ATCAGAAAAC AGCTGAAATT CTAGAAGCAT

RV_Am_US GATCCCTTCA AATGTTATTT ATCAGAAAAC AGCTGAAATT CTAGAAGCAT

RV_Vd_IL CTTCGGTTTG GATGTTACCA ATTCCTGAAC CAT...ATTT CTAGTGCCCT

Consensus gaTCccTTca aATGTTAttt ATcagaaAAC agctgaAaTT CTAGaagCaT

12051 12100

RV_Am_IL GTGATTTATC CAATTTTGCT A.....TACT ATCTAATATC TTCATAGATA

RV_Bi_US GTGATTTATC CAATTTTGCT A.....TACT ATCCAATATC TTCATAGATA

RV_Am_US GTGATTTATC CAATTTTGCT A.....TACT ATCCAATATC TTCATAGATA

RV_Vd_IL GTGGGTCGAG TGCGCTTCGC CCTTCGTAAT ACTCAATATC CCTTTTGGTA

Consensus GTGatTtatc caattTTgct a.....TAcT AtccAATATC ttcaTaGaTA

12101 12150

RV_Am_IL ATAGTTCAAA ACAGAGATTG .....TTGAA GTCGAAATAC AAGATAAGAG

RV_Bi_US ATAGTTCAAA ACAGAGATTG .....TTGAA GTCGAAATAC AAGATAAGAG

RV_Am_US ATAGTTCAAA ACAGAGATTG .....TTGAA GTCGAAATAC AAGATAAGAG

RV_Vd_IL ATCGACCATA GAATTCGGGG CGCCTTTTGA GCCGGAGCTT CTGATAAAAA

Consensus ATaGttCAaA acAgagattG .....TTgaA GtCGaAatac aaGATAAgAg

12151 12200

RV_Am_IL CCCCTTCTCA AACTGTTCCG AAGAGAATTA ACTATGCACG AACTGTACAA

RV_Bi_US CCCCTTCTCA AACTGTTCCG AAGAGAATTA ACTATGCACG AACTGTACAA

RV_Am_US CCCCTTCTCA AACTGTTCCG AAGAGAATTA ACTATGCACG AACTGTACAA

RV_Vd_IL CTGGTTTGGT CTCTTCAGCA TGTTTGATGA TCTCGGTGTT ATCGGAGCTG

Consensus CcccTTctca aaCTgttcCg aagagaATtA aCTatGcacg AaCtGtaCaa

12201 12250

RV_Am_IL TCGAGTTGTT GTGTTATCCT ATCAAAGCTA GCCTTAACTT CTAAACTTCC

RV_Bi_US TCGAGTTGTT GTGTTATCCT ATCAAAGCTA GCCTTAACTT CTAAACTTCC

RV_Am_US TCGAGTTGTT GTGTTATCCT ATCAAAGCTA GCCTTAACTT CTAAACTTCC

RV_Vd_IL ATGAAATGTT ......TATA ATCAACAGTA AGGTTGGGCG TGCTTCTTTA

Consensus tcGAgtTGTT gtgttaTcct ATCAAagcTA gccTTaactt ctaaaCTTcc

12251 12300

RV_Am_IL CTGGTTAGCG CCATCCCTTA TTATGATAAA CAAATACATC TCTGCCTTTG

RV_Bi_US CTGGTTAGCG CCATCCCTTA TTATGATAAA CAAATACATC TCTGCCTTTG

RV_Am_US CTGGTTAGCG CCATCCCTTA TTATGATAAA NAAATACATC TCTGCCTTTG

RV_Vd_IL ATGGTTGGCT TAGTAAAACG CAAACAGCTA AGAATTCTTT TTGAATAATG

Consensus cTGGTTaGCg ccaTccctta ttAtgAtaaA caAATaCaTc TctgccttTG

12301 12350

RV_Am_IL ATGCGTTATC TTTACACCTG GTAAATCAAT TAACACTACA AGGATACTTA

RV_Bi_US ATGCGTTATC TTTACACCTG GTAAATCAAT TAACACTACA AGGATACTTA

RV_Am_US ATGCGTTATC TTTACACCTG GTAAATCAAT TAACACTACA AGGATACTTA

RV_Vd_IL AGGAGCTTCG GATTTTCGAG TTGAACGTGA TTCGGTTCTG ATTTTGGTTA

Consensus AtGcGtTatc ttTacaCctG gTaAAtcaat TaacacTaca AggaTacTTA

12351 12400

RV_Am_IL CCTAGAGAAA AAATTGTGTA TCTTTTGGAT TTTTTGAACT CGCATGAATG

RV_Bi_US CCTAGAGAAA AAATTGTGTA TCTTTTGGAT TTTTTGAACT CGCATGAATG

RV_Am_US CCTAGAGAAA AAATTGTGTA TCTTTTGGAT TTTTTGAACT CGCATGAATG

RV_Vd_IL TGTGTGTGAA GATGTCAAAA TTGAATCTCT TATTACTTCT GGCTTCCTTG

Consensus ccTagagaAA aAatTgtgtA TctttTggaT TtTTtgaaCT cGCaTgaaTG

12401 12450

RV_Am_IL CTCTTCCTTA TTAGAGCTTA CAGACGGTAT TTATCTTAAA ATGCTTAATG

RV_Bi_US CTCTTCCTTA TTAGAGCTTA CAGACGGTAT TTATCTTAAA ATGCTTAATG

RV_Am_US CTCTTCCTTA TTAGAGCTTA CAGACGGTAT TTATCTTAAA ATGCTTAATG

RV_Vd_IL TTGTTTTTTA AAGGATCTGA CGGCGGCAAT AAACG...AA GGAGTCACCG

Consensus cTcTTccTTA ttaGAgCTtA CaGacGgtAT ttAtcttaAA atgcTtAatG

12451 12500

RV_Am_IL TTTCTCTGGA CTCTTTGATA AGAATGTTGC CCAGGTTACC ACAGATTCCA

RV_Bi_US TTTCTCTGGA CTCTTTGATA AGAATGTTGC CCAGGTTACC ACAGATTCCA

RV_Am_US TTTCTCTGGA CTCTTTGATA AGAATGTTGC CCAGGTTACC ACAGATTCCA

RV_Vd_IL TTCCTTTTTC AACTATGGCG CTGTAGATCT TATGTCTAGC CTCATCTTTT

Consensus TTtCTcTgga ctCTtTGata agaatGtTgc ccaGgtTAcC acagatTcca

12501 12550

RV_Am_IL AACACCAATA TATCGCTAAA ATTTAAAGTT CCTTCTAATA AATGTCTGCA

RV_Bi_US AACACCAATA TATCGCTAAA ATTTAAAGTT CCTTCTAATA AATGTCTGCA

RV_Am_US AACACCAATA TATCGCTAAA ATTTAAAGTT CCTTCTAATA AATGTCTGCA

RV_Vd_IL AGTAAAGATA AGTTTTTAAC AAATATGGAT TTGTCCTCAA AGGTAGCATT

Consensus AacAccaATA taTcgcTAAa AttTAaaGtT cctTCtaatA Aatgtctgca

12551 12600

RV_Am_IL ACACAAAAAG CAGGCACTAT ATAATACAAA ATATGGTAAG ATATCAATAC

RV_Bi_US ACACAAAAAG CAGGCACTAT ATAATACAAA ATATGGTAAG ATATCAATAC

RV_Am_US ACACAAAAAG CAGGCACTAT ATAATACAAA ATATGGTAAG ATATCAATAC

RV_Vd_IL TTTGATAATC TCGACAGCTT CCTTCCTAAT TTTTTCCTTA TTTTTTGTTT

Consensus acacAaAAag caGgCActaT ataatacAAa aTaTggtaag aTaTcaaTac

12601 12650

RV_Am_IL GCTACCCTAC ACCGATTACC GGAGCAATCT ACAAATATAT AGACGTTTTA

RV_Bi_US GCTACCCTAC ACCGATTACC GGAGCAATCT ACAAATATAT AGACGTTTTA

RV_Am_US GCTACCCTAC ACCGATTACC GGAGCAATCT ACAAATATAT AGACGTTTTA

RV_Vd_IL CCTCCTGTAT CTTAATGACT CTATTATACA AAGAATCAGT AGTCGTCAAT

Consensus gCTaCccTAc accgATtACc ggAgcAatCt AcaAATataT AGaCGTttta

12651 12700

RV_Am_IL AATCCTTACT TAGAAACAGA AATAGGTGAC GTTGTTTTCG TAATAGGTGA

RV_Bi_US AATCCTTACT TAGAAACAGA AATAGGTGAC GTTGTTTTCG TAATAGGTGA

RV_Am_US AATCCTTACT TAGAAACAGA AATAGGTGAC GTTGTTTTCG TAATAGGTGA

RV_Vd_IL GTTGCATCTG CTAATAGAGC .......... .....TTCTC GAACGGCTAA

Consensus aaTcCtTact tagAaAcAGa aataggtgac gttgtTTtcg tAAtaGgTgA

12701 12750

RV_Am_IL TGGAACCGGA GGAATAAGCT CATTACTATC TGTAT..... ..........

RV_Bi_US TGGAACCGGA GGAATAAGCT CACTACTATC TGTAT..... ..........

RV_Am_US TGGAACCGGA GGAATAAGCT CACTACTATC TGTAT..... ..........

RV_Vd_IL TGGAAGTTCA TGGAGCTTCT GCCAAATATC ACTATGTCTT GGTCCAGCTT

Consensus TGGAAccggA gGaAtaagCT cactAcTATC tgTAT..... ..........

12751 12800

RV_Am_IL .TGGGGAAGA AGGTTCTCTA TCAAACACTT ATTTCTTTTG ATCAAATTAC

RV_Bi_US .TGGGGAAGA AGGTTCTCTA TCAAACACTT ATTTCTTTTG ATCAAATTAC

RV_Am_US .TGGGGAAGA AGGTTCTCTA TCAAACACTT ATTTCTTTTG ATCAAATTAC

RV_Vd_IL CTTCAGAATA ATTTTCCTTG AACATTGCAA AAGGAGTTTT CTCCGCTTTC

Consensus .TgggGAAgA AggTTCtcTa tcaAacaCtt AtttctTTTg aTCaaaTTaC

12801 12850

RV_Am_IL ACAAAAT... .GCGTTAGGA ACAATAGTTC CTTCTGCTTA TTCATACTTA

RV_Bi_US ACAAAAT... .GCGTTAGGA ACAATAGTTC CTTCTGCTTA TTCATACTTA

RV_Am_US ACAAAAT... .GCGTTAGGA ACAATAGTTC CTTCTGCTTA TTCATACTTA

RV_Vd_IL ACGGACTTAT AGGCCGATTC TAATTGGGTC ATTTTGGTAT CATGTTCAGC

Consensus ACaaAaT... .GcgttAgga acAaTaGtTC cTTcTGcTta ttcaTaCtta

12851 12900

RV_Am_IL AAAGAGCAGC TGAAACCTCA TAACTGTGGG GAGTTTGTTG GATTTATTAA

RV_Bi_US AAAGAGCAGC TGAAACCTCA TAACTGTGGG GAGTTTGTTG GATTTATTAA

RV_Am_US AAAGAGCAGC TGAAACCTCA TAACTGTGGG GAGTTTGTTG GATTTATTAA

RV_Vd_IL AACGATCAAT TGAACTGAAG AGGCTACTTG ATCTAAGGTA TCTGATAAAT

Consensus AAaGAgCAgc TGAAacctca taaCTgtggG gagTttGtTg gaTttattAa

12901 12950

RV_Am_IL CGACTTGACT CATCCGGATT TCAGTATGTC CTGGATTTCC TCACTGAATT

RV_Bi_US CGACTTGACT CATCCGGATT TCAGTATGTC CTGGATTTCT TCACTGAATT

RV_Am_US CGACTTGACT CATCCNGATT TCAGTATGTC CTGGATTTCT TCACTGAATT

RV_Vd_IL TTTCTTGAGC TCTTTTCATT TCAACCATTT CTGATTTGAT TTCCGAAAGC

Consensus cgaCTTGAct caTccggATT TCAgtatgTc CTGgaTTtct TcaCtgAAtt

12951 13000

RV_Am_IL CCAATCAGGT GGTCGGAATT ATCTCAGATG CAGAGGGTGA TATATGGACG

RV_Bi_US CCAATCAGGT GGTCGGAATT ATCTCAGATG CAGAGGGTGA TATATGGACG

RV_Am_US CCAATCAGGT GGTCGGAATT ATCTCAGATG CAGAGGGTGA TATATGGACG

RV_Vd_IL AATCT...GT GGTCACTATT ATATAATTCT GCAGTTATAA CACTTTCAGC

Consensus ccaaTcagGT GGTCggaATT ATcTcAgatg cagagggTgA tAtaTggAcg

13001 13050

RV_Am_IL AAACCAGATT CGACGGATCA GGCTCTAGAA AATCTT.... ..TTTTGTTT

RV_Bi_US AAACCAGATT CGACGGATCA GGCTCTAGAA AATCTC.... ..TTTTGTTT

RV_Am_US AAACCAGATT CGACGGATCA GGCTCTAGAA AATCTC.... ..TTTTGTTT

RV_Vd_IL GAGTCTGTAA CCTTCTATGA GGCCGTCGAT AAAATCACAA TTTTTTGTCT

Consensus aAacCaGatt CgacggATcA GGCtcTaGAa AAtcTc.... ..TTTTGTtT

13051 13100

RV_Am_IL AATGACGAA. ......ACTT CAAAGTCTTA GGTTCTTAAG TGTTAAAATG

RV_Bi_US AATGACGAA. ......ACTT CAAAGTCTTA GGTTCTTAAG TGTTAAAATG

RV_Am_US AATGACGAA. ......ACTT CAAAGTCTTA GGTTCTTAAG TGTTAAAATG

RV_Vd_IL TATAAAAAAC AGATTTAGCT GTAAGCATTT GTCCCATTGC TTTAATTATG

Consensus aATgAcgAA. ......ActT caAAGtcTTa GgttCtTaag TgTtAaaATG

13101 13150

RV_Am_IL TATAATATCA CCTGCGATAG ATTAAAACAG ATCTTAACTA GGGTCTCAAA

RV_Bi_US TATAATATCA CCTGCGATAG ATTAAAACAG ATCTTAACTA GGGTCTCAAA

RV_Am_US TATAATATCA CCTGCGATAG ATTAAAACAG ATCTTAACTA GGGTCTCAAA

RV_Vd_IL TTTACTTTGG ACTGAGACAT TAAAAAACCA GTCAATGTTA ACTCGTCCTT

Consensus TaTAaTaTca cCTGcGAtAg attAAAACag aTCttaacTA gggtcTCaaa

13151 13200

RV_Am_IL TATAGGTTGG TCTCCGTTGA TAAAAGGCTC CAAGTTTTCT AACAGTAATA

RV_Bi_US TATAGGTTGG TCTCCGTTGA TAAAAGGCTC CAAGTTTTCT AACAGTAATA

RV_Am_US TATAGGTTGG TCTCCGTTGA TAAAAGGCTC CAAGTTTTCT AACAGTAATA

RV_Vd_IL TGTTTTAGTG TGTAGCGGAA GACCCTTTGG CCCGGTGTCT TCGGCCATAT

Consensus TaTaggttgG TcTccgttgA tAaaaggctc CaaGtTtTCT aacagtAata

13201 13250

RV_Am_IL CAGAGATGTT TCTAATATGT TTAAGAAATA TCGGACAAGA TACTGACAAA

RV_Bi_US CAGAGATGTT TCTAATATGT TTAAGAAATA TCGGACAAAA TGCTGACAAA

RV_Am_US CAGAGATGTT TCTAATATGT TTAAGAAATA TCGGACAAAA TGCTGACAAA

RV_Vd_IL TATCCAATGT AATGTTATCT TCGTGGTCCA TAGTCCAGTC AACTGCGTTG

Consensus cAgagAtgtT tcTaaTATgT TtaaGaaatA TcGgaCAaaa taCTGacaaa

13251 13300

RV_Am_IL GCCTCAATGA TTAAATTAG. ATAATCCAGA ACAGTCACTT AACAAGGCAC

RV_Bi_US GCCTCAATGA NNAAATTAG. ATAATCCAGA ACAGTCACTT AACAAGGCAC

RV_Am_US GCCTCAATGA TTAAATTAG. ATAATCCAGA ACAGTCACTT AACAAGGCAC

RV_Vd_IL GTGTTTATGT CAAGGTTGTC ATCGTCTAAT GATGCTGCGT TTTCAAACTC

Consensus GccTcaATGa ttAaaTTag. ATaaTCcAga acaGtcaCtT aacaAggCaC

13301 13350

RV_Am_IL TTCGGGCCAG TGCATTATGG CCGTGTGAAT GCGTAATTAG CAGATTCAGA

RV_Bi_US TTCGGGCCAG TGCATTATGG CCGTGTGAAT GCGTAATTAG CAGATTCAGA

RV_Am_US TTCGGGCCAG TGCATTATGG CCGTGTGAAT GCGTAATTAG CAGATTCAGA

RV_Vd_IL ATATGCCAGT CCGGATTTAG AGGCTTTATT GAATTTTTT. ...AAGTAGG

Consensus tTcgGgCcag tgcatTaTgG ccGtgTgAaT GcgTaaTTag cagAttcAGa

13351 13400

RV_Am_IL AAAGCAGTTC TTGAAGTATA TGACTTAGAA GACAATTGTT ATGATGCTCT

RV_Bi_US AAAGCAGTTC TTGAAGTATA TGACTTAGAA GACAATTGTT ATGATGCTCT

RV_Am_US AAAGCAGTTC TTGAAGTATA TGACTTAGAA GACAATTGTT ATGATGCTCT

RV_Vd_IL TCACTCATTT TGGCTTCTAT TGGGTTTTGA GGGACTTCTT TGACTTGGAG

Consensus aaAgcagTTc TtGaagtata TGacTTagaA GacAaTTgTT atgaTgctct

13401 13450

RV_Am_IL AAGTATGGAA CTGCCTAATG AGCTGAAAGG ACTAGTCAGC AACTTAGAGC

RV_Bi_US AAGTATGGAA CTGCCTAATG AGCTGAAAGG ACTAGTCAGC AACTTAGAGC

RV_Am_US AAGTATGGAA CTGCCTAATG AGCTGAAAGG ACTAGTCAGC AACTTAGAGC

RV_Vd_IL CGTTTTTGAC CTTTCTAGGG TGCAGGGGTG TGCACCTATC TGTGT.GTTC

Consensus aagTaTgGAa CTgcCTAatG aGCtGaaagG actAgtcAgC aactTaGagC

13451 13500

RV_Am_IL TGGACATACC TAGACTAGTT GCATGTCTTT TAGAGAAGAC CATTATCAAC

RV_Bi_US TGGACATACC TAGACTAGTT GCATGTCTTT TAGAGAAGAC AATTATCAAC

RV_Am_US TGGACATACC TAGACTAGTT GCATGTCTTT TAGAGAAGAC AATTATCAAC

RV_Vd_IL CGGTGGTGCG TGCAGCGGTT GCGGGACTGT TTGAACGGGA TGTT......

Consensus tGGacaTaCc TagActaGTT GCatGtCTtT TaGAgaaGac aaTTatcaac

13501 13550

RV_Am_IL TTCACCAAAA CTTTAGGTAA TTGGGGTTGT GTTAAGAAAA AGAATAAGAT

RV_Bi_US TTCACCAAAA CTTTGGGTAA TTGGGGTTGT GTAAAGAAAA AGAATAAGAT

RV_Am_US TTCACCAAAA CTTTGGGTAA TTGGGGTTGT GTAAAGAAAA AGAATAAGAT

RV_Vd_IL .......... .TGAGTTTGA GTGTGGTTGA TTGCTGAATC GGAGTGTGTT

Consensus ttcaccaaaa cTttgggTaA tTGgGGTTGt gTaaaGAAaa aGAaTaaGaT

13551 13600

RV_Am_IL ATTAAAGGAA CAAAAAATTA GGAAAATTAT ATACACTGCT GCAAACCTTT

RV_Bi_US ATTAAAGGAA CAAAAAATTA GGAAAATTAT ATACACTGCC GCAAACCTTT

RV_Am_US ATTAAAGGAA CAAAAAATTA GGAAAATTAT ATACACTGCC GCAAACCTTT

RV_Vd_IL GACTGGGGTC AGATGGAGCT GCTTCTTCTG GTTGAGAGAT TGAGAGCTGC

Consensus attaaaGGaa caAaaaAtta GgaaaaTtat aTacActGcc gcAaAcCTtt

13601 13650

RV_Am_IL TTCTATCTCT TATATCTATT GCTCCAGTGG AAGTATCTGA ATCTATAAGG

RV_Bi_US TCCTATCTCT TATATCTATT GCTCCAGTGG AAGTATCTGA ATCTATAAGG

RV_Am_US TCCTATCTCT TATATCTATT GCTCCAGTGG AAGTATCTGA ATCTATAAGG

RV_Vd_IL TG..AGCTTA AGTTGTTGTT GAGAAAGAGG GGATATGTAT TTTTTATAGT

Consensus TcctAtCTct taTatcTaTT GctccAGtGG aagTATcTga aTcTataAGg

13651 13700

RV_Am_IL AAAATAGATA AAATCGCAAC TCGGATAGTT GCTAAAGTTA GTTTAATAAA

RV_Bi_US AAAATAGATA AAATCGCAAC TCGGATAGTT GCTAAAGTTA GTTTAATAAA

RV_Am_US AAAATAGATA AAATCGCAAC TCGGATAGTT GCTAAAGTTA GTTTAATAAA

RV_Vd_IL GAGTTTGGTT CTGAGTGGTT TGGG...GTT GCTCTGGTTG CTTTGATGTC

Consensus aAaaTaGaTa aaatcgcaac TcGGataGTT GCTaaaGTTa gTTTaATaaa

13701 13750

RV_Am_IL AGGGAAAGGT TACCTGAGTT TTGCTATTGA AAAGGGAACA AAAAAGAATC

RV_Bi_US AGGGAAAGGC TACCTGAGCT TTGCTATTGA AAAGGGAACA AAAAAGAATC

RV_Am_US AGGGAAAGGC TACCTGAGTT TTGCTATTGA AAAGGGAACA AAAAAGAATC

RV_Vd_IL CGGCTGCTGA CTTGGGGAGT TTCGGAGATC AGGGTGGATT TGGGGTGTTC

Consensus aGGgaaagGc tacctGagtT TTgctAttga AaaGgGaAca aaaaagaaTC

13751 13800

RV_Am_IL ATAACACTTG GATAGTTCCG TTATATAAAG GTAAGGAAGG TTTAATAATT

RV_Bi_US ATAACACTTG GATAGTTCCG TTATATAAAG GNAAGGAAGG TTTAATAATT

RV_Am_US ATAACACTTG GATAGTTCCG TTATATAAAG GCAAGGAAGG TTTAATAATT

RV_Vd_IL ATGGGGGTCT TGGGGTGACT TCAGCTT..G GTGTTTCATG TTAAACTTTC

Consensus ATaacacTtg gataGTtcCg TtAtaTaaaG GtaaggaAgG TTtAAtaaTt

13801 13850

RV_Am_IL AAGAAGGCAT ATAGAATAAT TGGTGCCTTG TTATTGGAAA AGCATCACGT

RV_Bi_US AAGAAGGCAT ATAGAATAAT TGGTGCCTTG TTATTGGAAA AGCATCACGT

RV_Am_US AAGAAGGCAT ATAGAATAAT TGGTGCCTTG TTATTGGAAA AGCATCACGT

RV_Vd_IL AGCAAGGACT CAATGTCAGC TTCCTGTTCG TCATCAAAAA ATCCGGCAAA

Consensus AagAAGGcaT atAgaatAat TggtgccTtG TtATtggAAA AgCatcacgt

13851 13900

RV_Am_IL TAAGCTAAGC TCCTTTTCAC CGACTACAGT TACTGTAGAT CCTTTTAAAC

RV_Bi_US TANGCTAAGC TCCTTTTCAC CGACTACAGT TACTGTAGAT CCTTTTAAAC

RV_Am_US TAAGCTAAGC TCCTTTTCAC CGACTACAGT TACTGTAGAT CCTTTTAAAC

RV_Vd_IL CTTTCTTTCA GAGTCCTCAC TTGACATCTT CTCTACCCAC CGCTTTAAAT

Consensus taagCTaagc tccTttTCAC cgactAcagT taCTgtagAt CctTTTAAAc

13901 13950

RV_Am_IL TTAGAAGCAA GGGTAGGTGG TTGATGATTG ATGACAGCTT CACTTTTTTC

RV_Bi_US TTAGAAGCAA GGGTAGGTGG TTGATGATTG ATGACAGCTT CACTTTTTTC

RV_Am_US TTAGAAGCAA GGGTAGGTGG TTGATGATTG ATGACAGCTT CACTTTTTTC

RV_Vd_IL GGACTTCATC TGCCGCTTGG TGTACTGTTG CTGAATATCC CGCTGATTT.

Consensus ttAgaagcaa gGgtaggTGG TtgAtgaTTG aTGAcagctt CaCTttTTTc

13951 14000

RV_Am_IL TTCCCAGGAG GAGATAATAA CTGTATATTT TTACCTTCTG TCATTCGTAC

RV_Bi_US TTCCCAGGAG GAGATAATAA CTGTATATTT TTACCTTCTG TCATTCGTAC

RV_Am_US TTCCCAGGAG GAGATAATAA CTGTATATTT TTACCTTCTG TCATTCGTAC

RV_Vd_IL ........AA ATGCTTCAAA ATCAGCCATT CTATCGCTTT TTAAGCAGAT

Consensus ttcccaggAg gaGaTaatAA cTgtatatTT tTAcCttcTg TcAttCgtAc

14001 14050

RV_Am_IL ATACTATCCT GGTGAAGCTG GTTTTAATCA AGAAATGAAT ....ACAAAT

RV_Bi_US ATANTATCCT GGTGAAGCTG GTTTTAATCA AGAAATGAAT ....ACAAAT

RV_Am_US ATATTATCCT GGTGAAGCTG GTTTTAATCA AGAAATGAAT ....ACAAAT

RV_Vd_IL GCAATATAGA AGTGCTGCAG ACATTCTTTT CGATTCCATT CTGGACAGAT

Consensus atA.TATcct gGTGaaGCtG gttTTaaTca aGAaatgAaT ....ACAaAT

14051 14100

RV_Am_IL CTGAGCTTAA ACTATTCAGA CGAAGATCCC AATGATGAGA ACCAAGGGGA

RV_Bi_US CTGAGCTTAA ACTATTCAGA CGAAGATCCC GATGATGAAA ACCAAGGGGA

RV_Am_US CTGAGCTTAA ACTATTCAGA CGAAGATCCC GATGATGAAA ACCAAGGGGA

RV_Vd_IL CGGAGAAGAA ACCTGTGT.. .......... .......... ..CCAGGGTT

Consensus CtGAGcttAA ACtatTcaga cgaagatccc gatgatgaaa acCaAGGGga

14101 14150

RV_Am_IL AAGTGATGAA GAAGATGTTT AAACATTTTT TCTAAAAACT TTAAAAATGC

RV_Bi_US AAGTGATGAA GAAGATGTTT AAACATNTTT TCTAAAAACT TTAAAAATGC

RV_Am_US AAGTGATGAA GAAGATGTTT AAACATTTTT TCTAAAAACT TTAAAAATGC

RV_Vd_IL CGAGTCACCA TAGGCTGGTG TTTTGTTTTC CCTGATAGGT TGCATTTTTC

Consensus aagtgatgaA gAaGaTGtTt aaacaTtTTt tCTaAaAacT TtaAaaaTgC

14151 14200

RV_Am_IL TTTCTAAACT TAAATCACAA AAGATTGGGA AAACAAAAGA ACCGAAGAGA

RV_Bi_US TTTCTAAACT TAAATCACAA AAGATTGGGA AAACAAAAGA ACCGAAGAGA

RV_Am_US TTTCTAAACT TAAATCACAA AAGATTGGGA AAACAAAAGA ACCGAAGAGA

RV_Vd_IL TATCGCTCCC CAGTACTCCA AAA...GGGC AATCATCCCT GCCCTAAAAT

Consensus TtTCtaaaCt tAaatCaCaA AAgattGGGa AAaCAaaaga aCCgaAgAga

14201 14250

RV_Am_IL TCTCATTAGA GATTCGTTAC CAAAAATCTA ACCCTAAATA GGTTGACAAC

RV_Bi_US TCTCATTAGA GATTCGTTAC CAAAAATCTA ACCCTAAATA GGTTGACAAC

RV_Am_US TCTCATTAGA GATTCGTTAC CAAAAATCTA ACCCTAAATA GGTTGACAAC

RV_Vd_IL CACCATTAGT CAA....... .ATAATTTTC ACTCTCAAAA GCTGTAGGAA

Consensus tctCATTAGa gAttcgttac cAaAAaTcTa ACcCTaAAtA GgTtgAcaAc

14251 14300

RV_Am_IL ATTCTACCAT TTCCTTGGAG AGAACACATC TTAAACCAAC TAATGCTTCA

RV_Bi_US ATTCTACCAT TTTCTTGGTG AGAACACATC TTAAACCAAC TAATGCTTCA

RV_Am_US ATTCTACCAT TTTCTTGGTG AGAACACATC TTAAACCAAC TAATGCTTCA

RV_Vd_IL GTAATTTTGT AAATTCGG.. ....CATATC TCCTCGAAAG TACAGTTGAT

Consensus aTtcTaccaT tttcTtGGtg agaaCAcATC TtaaaccAAc TAatGcTtca

14301 14350

RV_Am_IL GCCTAAATTC ACTTCACTTC TAGAGATTAC AACAAAACTA CCAACCTGGC

RV_Bi_US GCCTAAATTC ACTTCACTTC TAGAGATTAC AACAAAACTA CCAACCTGGC

RV_Am_US GCCTAAATTC ACTTCACTTC TAGAGATTAC AACAAAACTA CCAACCTGGC

RV_Vd_IL TGAGAACAGA TCGGGCGATA TAAGGCATAA AACATCATAG CTTCCCAGTG

Consensus gcctAAattc aCttcactTc TAgaGatTAc AACAaaActa CcaaCCtGgc

14351 14400

RV_Am_IL CTGTTCCTCT CGTGTCCTAA CATCTCCAGA AAGCTGAAGT GACAGATTAA

RV_Bi_US CTGTTCCTCT CGTGTCCTAA CATCTCCAGA AAGCTGAAGT GACAGATTAA

RV_Am_US CTGTTCCTCT CGTGTCCTAA CATCTCCAGA AAGCTGAAGT GACAGATTAA

RV_Vd_IL AGGTACGTCA ...GTATTGT CTAGTAGTAA AAGCGAATGT GTCAGAGTT.

Consensus ctGTtCcTCt cgtGTccTaa CatcTccagA AAGCtgAaGT GaCAGAtTaa

14401 14450

RV_Am_IL CCGGGACTAC GTTGAAGCAT CTCAAAACCA ACTACACCTT TGTCGGTGTC

RV_Bi_US CCGGGACTAC GTTGAAGCAT CTCAAAACCA ACCACACCTT TGTCGGTGTC

RV_Am_US CCGGGACTAC GTTGAAGCAT CTCAAAACCA ACCACACCTT TGTCGGTGTC

RV_Vd_IL ....GAATGT ATATCATAAT CTGTAGAGCT AATCTGGGGT AATCTCAGCA

Consensus ccggGAcTac gTtgaAgcAT CTcaAaAcCa AccacacctT tgTCggtGtc

14451 14500

RV_Am_IL TCCAATGGCT TGACTTGAGA GTTTAATGAG TAATCTTCAA TTTAATATTC

RV_Bi_US TCCAATGGCT TGACTTGAAA GTTTAATGAG TAATCTTCAA TTTAATATTC

RV_Am_US TCCAATGGCT TGACTTGAAA GTTTAATGAG TAATCTTCAA TTTAATATTC

RV_Vd_IL TGCCATTAAT AATCTCAGGA GATAAAGTAG GTCCAGAGAT ATTTAGTATC

Consensus TcCaATggcT tgaCTtgaaA GtTtAAtgAG taatcttcAa tTTaAtatTC

14501 14550

RV_Am_IL TTCTTTGTTA ACTAAAAAAG TTGTATCGAT ATCTTTCCCA CTTTATACAG

RV_Bi_US TTCTTTGTTA ACTAAAAAAG TTGTATCAAT ATCTTTCCCA CTTTATACAG

RV_Am_US TTCTTTGTTA ACTAAAAAAG TTGTATCAAT ATCTTTCCCA CTTTATACAG

RV_Vd_IL TTTTCTATAT TTCCACAGAA CT.TGCCTAT TTGGTTAGCT CTACCCGTCT

Consensus TTcTtTgTta actaAaAaAg tTgTatCaAT aTctTTccCa CTttatacag

14551 14600

RV_Am_IL ATAGCTGTTT CCAAAGTTGA TTATCCTGTG AGATCCATCA TTTGAGATCT

RV_Bi_US ATAGCTGTTT CCAAAGTTGA TTATCCTGTG AGATCCATCA TTTGAGATCT

RV_Am_US ATAGCTGTTT CCAAAGTTGA TT........ .......... ..........

RV_Vd_IL TCCGCATGGA CCAGCCGACA AGGTACGCAG CAAACGCAAG TATCCAATCT

Consensus ataGCtgttt CCAaagttgA ttatcctgtg agatccatca tttgagatct

14601 14650

RV_Am_IL TCTTTCACGC GCAAGCAATT A......ACG ATCCCTATAC CTCATTACCG

RV_Bi_US TCTTTCACGC GTAAGCAATT A......ACG ATCCCTATAC CTCATTACCG

RV_Am_US .......... .......... .......... .......... ..........

RV_Vd_IL TCTGTTAAGT GTTCCGGATT CTCTCGGACC CATGCTATTA CTTCCTGAGT

Consensus tctttcacgc gtaagcaatt a......acg atccctatac ctcattaccg

14651 14700

RV_Am_IL TTAAGCCCTA AACTTATAAT AAGTTTTTTT ATTAATGAGG CTTGGAGACA

RV_Bi_US TTAAGCCCTA AACTTATAAT AAGTTTTTTG .TTAATGAGG CNTGGAGACA

RV_Am_US .......... .......... .......... .......... ..........

RV_Vd_IL AGGAACTGGA GGCGTAAAAG ATGCAATTGT CATTGATGTC GGACCTTTCT

Consensus ttaagcccta aacttataat aagttttttt .ttaatgagg c.tggagaca

14701 14750

RV_Am_IL CAGCAGTGCT CTGAGTGGCT GTTACAGCTC CAATAATCTC AATATGGGCA

RV_Bi_US CAGCAGTGCT CTGAGTGGCT GTTACAGCTC CAATAATCTC AATATGGGCA

RV_Am_US .......... .......... .......... .......... ..........

RV_Vd_IL CAATTGTCCC GATTTTGGAT TTGACGGATT CAACAAATAA AGCTTGCGCC

Consensus cagcagtgct ctgagtggct gttacagctc caataatctc aatatgggca

14751 14800

RV_Am_IL ATTCACAAAG ATACCGTGTC CTCAGGAAAG CTCCAAAATA CTTGGCTGCA

RV_Bi_US ATTCACAAAG ATACCGTGTC CTCAGGAAAG CTCCAAAATA CTTGGCTGCA

RV_Am_US .......... .......... .......... .......... ..........

RV_Vd_IL ACTCTATACG TTG....... .......ATG CTAGACCTTC AGTTACTGCA

Consensus attcacaaag ata....... .......aag ctccaaaata cttggctgca

14801 14850

RV_Am_IL GCGACAGGGC AGTCTGTAAG GGGTCTGTGG ATCAACGAGC CGGAAAACAA

RV_Bi_US GCGACAGNGC AGTCTGTAAG GGGTCTGTGG ATCAACGAGC CGGAAAACAA

RV_Am_US .......... .......... .......... .......... ..........

RV_Vd_IL GTTA.ATAAA TGTACGATGG AAATTGTTGG TGCAACATGA CAAATAAAGG

Consensus gcga.ag.gc agtctgtaag gggtctgtgg atcaacgagc cggaaaacaa

14851 14900

RV_Am_IL TGTTCGAAAA AGTTCCAAAT ATTTCAATGA GTTTTCTGTC CAGGAGAATC

RV_Bi_US TGTTCGAAAA AGTACCAAAT ATNTCAATGA GTTTTCTGTC CAGGAGAATC

RV_Am_US .......... .......... .......... .......... ..........

RV_Vd_IL GAATCGAAAG GTACGAGTCA ACCTCAAAGT TTTTCGGGGT CGGTTGTGTT

Consensus tgttcgaaaa agt.ccaaat at.tcaatga gttttctgtc caggagaatc

14901 14950

RV_Am_IL ATTCACACAT TTTCCCCAAG TGAATACAGC TCAAACAGAG AGCAATCAAC

RV_Bi_US ATTCACACAT TTTCCCCAAG TGAATACAGC TCAAACAGAG AGCAACCAAC

RV_Am_US .......... .......... .......... .......... ..........

RV_Vd_IL T......... ......CAGA TGCGGAGAGC TTCCCTAAAC GTTTTGCGAG

Consensus a......... ......caag tgaatacagc tcaaacagag agcaa.caac

14951 15000

RV_Am_IL ATGCTCAACG CCCAGACCAC AAGGGAAGAC CACTCATTAT TAAAAAA.AC

RV_Bi_US ATGCTCAACG CCCAGACCAC AAGGGAAGAC CACTCATTAT TAAAAAA.AC

RV_Am_US .......... .......... .......... .......... ..........

RV_Vd_IL CGCCTGAATT TGCGAGTTCG AAAGGCTGGC CATTATTGCT TTAAAGATAC

Consensus atgctcaacg cccagaccac aagggaagac cactcattat taaaaaa.ac

15001 15050

RV_Am_IL AAAATGTTAA TTTCAATCGG TCTAATCGTT TATTCCAGAT TCACAGGTCC

RV_Bi_US AAAATGTTAA TTTCAATCGG TCTAATCATT TATTCCAGAT TCACAGGTCC

RV_Am_US .......... .......... .......... .......... ..........

RV_Vd_IL TGACTGGTGT CAATGTGCTA AATGTTCTCA ATATTAAGTA TAAAAGTTAT

Consensus aaaatgttaa tttcaatcgg tctaatc.tt tattccagat tcacaggtcc

15051 15100

RV_Am_IL GGCTGGATAA TCCAGATCTT TTGGACTTAC AAGACAAGTC TAAATCGGTC

RV_Bi_US GGCTGGATAA TCCAGATCTT TTGGACTTAC AAGACAAGTC TAAATCGGTC

RV_Am_US .......... .......... .......... .......... ..........

RV_Vd_IL AAAAAGACTA AACAGATTAA TCTCAATGAA TAGACCTTTT TAAATAAG..

Consensus ggctggataa tccagatctt ttggacttac aagacaagtc taaatcgg..

15101 15150

RV_Am_IL CCAGATCGTT TCGGTCTCAA AGCAGTCAAT GATTTTTGAA AAGGGATTGA

RV_Bi_US CCAGATCGTT TCGGTCTCAA AGCAGTCAAT GATTTTTGAA AAGGGATTGA

RV_Am_US .......... .......... .......... .......... ..........

RV_Vd_IL .......... .......... ...AGTTAAT ACTGGTTCTA AAGTTTCTTG

Consensus .......... .......... ...agtcaat gatttttgaa aagggattga

15151 15200

RV_Am_IL CGGCAAATTA GATCGATACA AAGGCGAATG GTGTGCCCGT TGGTGTTATA

RV_Bi_US CGGCAAATTA GATCGATACA AAGGCGAATG GTGTGCCCGT TGGTGTTATA

RV_Am_US .......... .......... .......... .......... ..........

RV_Vd_IL CTCAATCTTC CAAAGGCATG AAGAAGCAAA GCGTCCG... ..........

Consensus cggcaaatta gatcgataca aaggcgaatg gtgtgcc... ..........

15201 15250

RV_Am_IL GCCGGATTCA AACGGCAATT AGCAATAGAT ATAAATGACA AAAAATGGCC

RV_Bi_US GCCGGATTCA AACGGCAATT AGCAATAGAT ATAAATGACA AAAAATGGCC

RV_Am_US .......... .......... .......... .......... ..........

RV_Vd_IL GACGGTACAT CAGTCCACGA AAAAACATTT CCTAACGGCT TGACCGTTCC

Consensus gccggattca aacggcaatt agcaatagat ataaatgaca aaaaatggcc

15251 15300

RV_Am_IL CGATACTAGT GTACCAAGTC ACATCAGTCA TCTATAAATA TTTTACAGAA

RV_Bi_US CGATACTAGT GTACCAAGTC ACATCAGTCA TCTATAAATA TTTTACAGAA

RV_Am_US .......... .......... .......... .......... ..........

RV_Vd_IL CCCCAGTAAG AACCCCAGAC AAAAGATGGT TTGATACT.. ..........

Consensus cgatactagt gtaccaagtc acatcagtca tctataaa.. ..........

15301 15341

RV_Am_IL ACGAAGATGC ATTTTTGGTC TGTTTGAGCT GTATTCACTT G

RV_Bi_US AAAAAGATGC ATTTTTGGTC TGTTTGTGCG .......... .

RV_Am_US .......... .......... .......... .......... .

RV_Vd_IL .......... ATTTTTGGTC TGTTGTAGCG TCA....... .

Consensus .......... atttttggtc tgtttgagcg .......... .

**Supplementary Figure 1. Alignment of Apis rhabdovirus-1 (RV) nucleotides**

Am_IL and Am_US, *Apis mellifera* sequences from Israel and US, respectively. Bi_US,

*Bombus impatiens* _US sequence. Vd_IL, *Varroa destructor* Israel sequences.

**Supplementary images 2A and B, 3 and 4A and 4B** (those are the original captions of Figures 2 A and B; Figure 3 of the manuscript and Supplementary Figure 4 - embedded below.

# Legend for Original Image of Figure S2A (named Supplementary Image 2). Order identical to Figure 3 in the manuscript. Compare labels: 1kb=M, marker. The same order of lanes was kept than those in the text, namely: lanes 3,4 = lanes B1P 1+2 and 3+4, respectively; lanes 5,6 = B1P 1+2 and 3+4, respectively; lanes 7,8= lanes V1P 1+2 and 3+4, respectively; lanes 9,10= lanes V2P 1+2 and 3+4, respectively; lanes 11,12= NTC lanes 1+2 and 3+4, respectively; P= size of positive controls was cut out and correspond to the arrow in the text.

**Legend for Original Image of Figure S2B** (**named Supplementary Image 5**)**.** Annotated in the figure under *A. mellifera* (U.S.) and *B. impatiens* (U.S.). Numbers reflect individual bee samples. On the first and last lanes of the gel the Molecular marker were loaded (please note that the right one lane of this marker was deleted in Figure 2B for uniformity with figure 2A). NEG= NTC.

**Legend for Original image of Figure S3.** Labels from M to V4c of the upper gel identical to those of Figure 4 in the manuscript. NTC, non-template control. P=Positive control.

Labels of the upper gel: M= Marker; from B1 to NTC identical to those of Figure 4 that follow the lane V4c in the manuscript. These lanes were mounted in the manuscript to make a continuous figure to save space following the v4c lane. NTC, non-template control. P=Positive control replaced by an arrow in the manuscript.

**Legend for Original image of Figure S4A (named Supplementary Image 6),** exactly as in the embedded Supplementary Figure 4A, see below.

**Legend for Original image of Figure S4B, (named Supplementary Image 7),** exactly as in the embedded Supplementary Figure 4A, see below.

**Figure S4.** Detection of the ARV-1 positive-sense RNA strand in *V. destructor* (A) and *A. mellifera* (B). V1...V4, the numbers indicate the individual Varroa tested. B1…B5, the numbers indicate the individual bees tested. PCR primers: p1= BRV-1F-10356-R and TAG-D_F. Suffix p1 indicates PCR control reaction from the same individual performed on cDNA with the primer 1 only. Suffix C indicates PCR control reaction from the same individual RNA performed on cDNA produced without any primer (see Materials and Methods and Table 1). NTC, non-template control. NTCp1, non-template control with primer 1 only. M = 1 kb DNA Marker Ladder (Thermo Scientific Inc.). Arrow: ARV-1 amplicon (see Materials and Methods).
