# Supplementary figures and images for: Presence of Apis Rhabdovirus-1 in Populations of Pollinators and Their Parasites from Two Continents
Source: Front Microbiol. 2017 Dec 12;8:2482. doi: 10.3389/fmicb.2017.02482 (PMC5732965; doi:10.3389/fmicb.2017.02482)

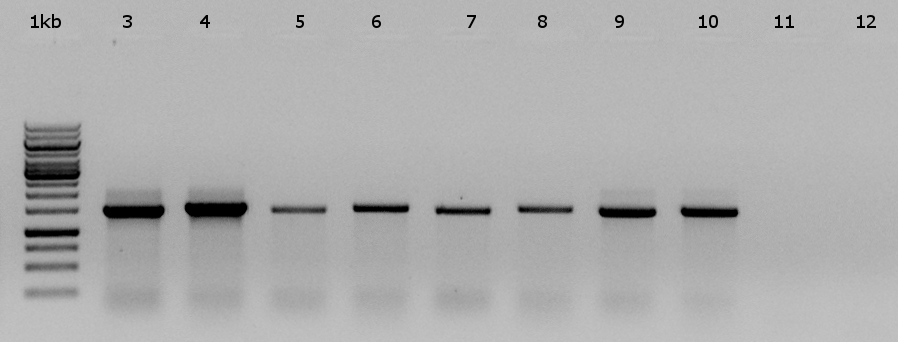

Supplement: Supplementary file 2 [file Image_1.tif]

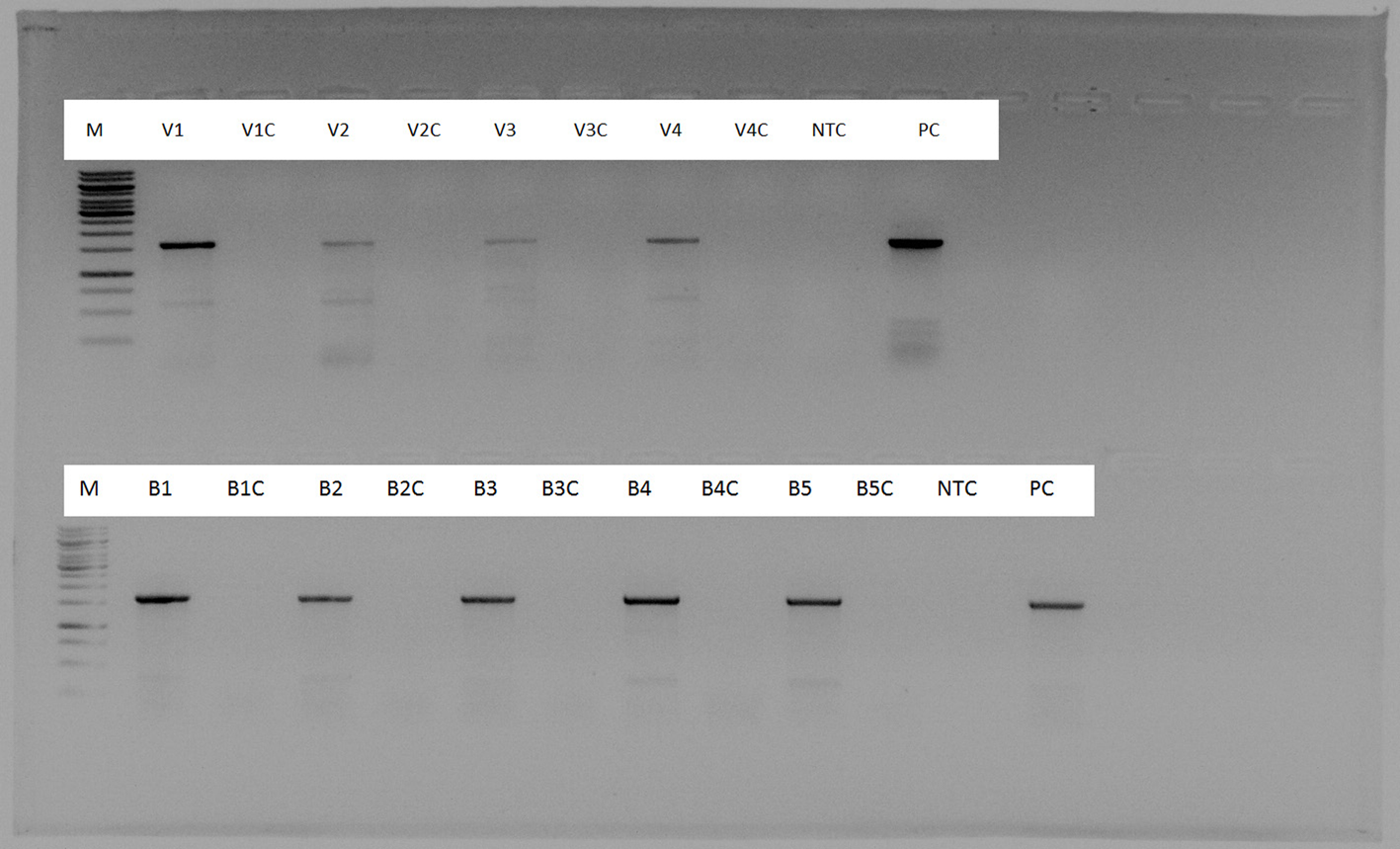

Supplement: Supplementary file 3 [file Image_2.tif]

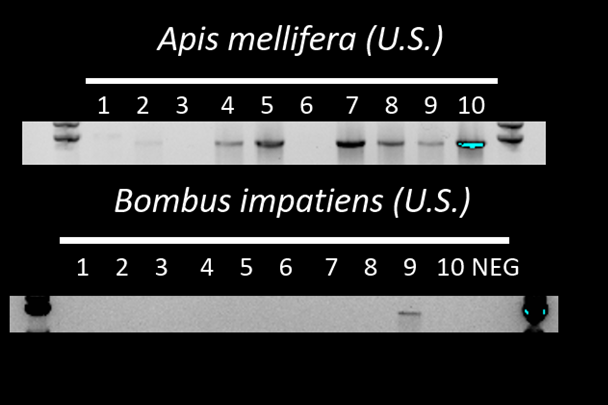

Supplement: Supplementary file 4 [file Image_3.tif]

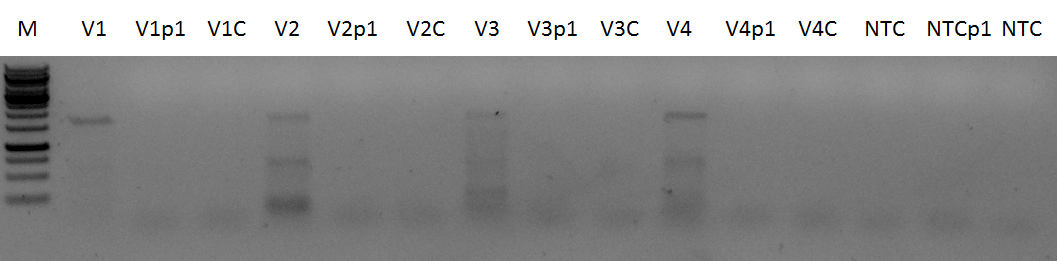

Supplement: Supplementary file 5 [file Image_4.tif]
